# The discovery and enhanced properties of trichain lipids in lipopolyplex gene delivery systems†
†Electronic supplementary information (ESI) available: This includes detailed experimental procedures, characterisation data, and supplementary Fig. S1–S6. See DOI: 10.1039/c8ob02374c


**DOI:** 10.1039/c8ob02374c

**Published:** 2018-12-19

**Authors:** Atefeh Mohammadi, Laila Kudsiova, M. Firouz Mohd Mustapa, Frederick Campbell, Danielle Vlaho, Katharina Welser, Harriet Story, Aristides D. Tagalakis, Stephen L. Hart, David J. Barlow, Alethea B. Tabor, M. Jayne Lawrence, Helen C. Hailes

**Affiliations:** a Department of Chemistry , University College London , Christopher Ingold Laboratories , 20 Gordon Street , London WC1H 0AJ , UK . Email: h.c.hailes@ucl.ac.uk; b Faculty of Life Sciences and Medicine , King's College London , Franklin-Wilkins Building , Stamford Street , London SE1 9NH , UK; c Wolfson Centre for Gene Therapy of Childhood Disease , Institute of Child Health , University College London , 30 Guilford Street , London WC1N 1EH , UK; d Division of Pharmacy and Optometry , School of Health Sciences , Faculty of Biology , Medicine and Health , Stopford Building , Manchester University , Oxford Road , Manchester , M13 9PT , UK . Email: jayne.lawrence@manchester.ac.uk

## Abstract

The formation of a novel trichain (TC) lipid was discovered when a cationic lipid possessing a terminal hydroxyl group and the helper lipid dioleoyl l-α-phosphatidylethanolamine (DOPE) were formulated as vesicles and stored. Importantly, the transfection efficacies of lipopolyplexes comprised of the TC lipid, a targeting peptide and DNA (LPDs) were found to be higher than when the corresponding dichain (DC) lipid was used. To explore this interesting discovery and determine if this concept can be more generally applied to improve gene delivery efficiencies, the design and synthesis of a series of novel TC cationic lipids and the corresponding DC lipids was undertaken. Transfection efficacies of the LPDs were found to be higher when using the TC lipids compared to the DC analogues, so experiments were carried out to investigate the reasons for this enhancement. Sizing experiments and transmission electron microscopy indicated that there were no major differences in the size and shape of the LPDs prepared using the TC and DC lipids, while circular dichroism spectroscopy showed that the presence of the third acyl chain did not influence the conformation of the DNA within the LPD. In contrast, small angle neutron scattering studies showed a considerable re-arrangement of lipid conformation upon formulation as LPDs, particularly of the TC lipids, while gel electrophoresis studies revealed that the use of a TC lipid in the LPD formulation resulted in enhanced DNA protection properties. Thus, the major enhancement in transfection performance of these novel TC lipids can be attributed to their ability to protect and subsequently release DNA. Importantly, the TC lipids described here highlight a valuable structural template for the generation of gene delivery vectors, based on the use of lipids with three hydrophobic chains.

## Abstract 2

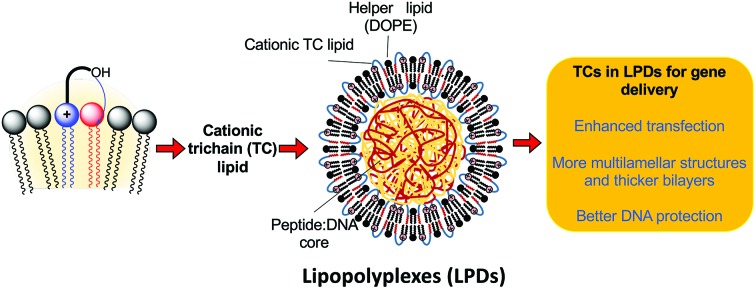
Novel trichain lipids have been identified with enhanced transfection properties in lipopolyplexes.

## Introduction

**Fig. 1:**
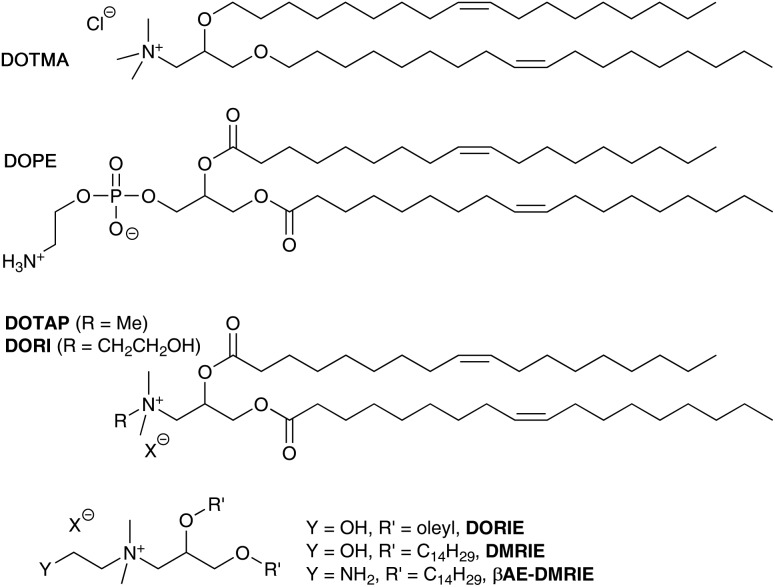
Structures of a selection of cationic lipids: DOTMA, DOTAP, DORI, DORIE, DMRIE, βAE-DMRIE and the helper lipid DOPE.

Non-viral vectors continue to have significant potential to facilitate DNA or RNA delivery into target cells.^1^ Synthetic cationic lipids (cytofectins) have been used in many lipid-DNA formulations, and since the report of the cytofectin DOTMA^2^ which is often used with the neutral lipid dioleoyl l-α-phosphatidylethanolamine (DOPE) (Fig. 1), a range of related lipids have been described.1e,^3^ The aim in developing new cytofectins has been to optimise the delivery properties of the resulting vector for a wide variety of cell types, and for *in vivo* applications.^2^,^3^ Cytofectins are typically used in lipoplex (lipid-DNA; LD) binary formulations or lipopolyplex (lipid-polycation/peptide-DNA; LPD) ternary formulations where the polycation/peptide has DNA-binding functionality and peptides can also have cell-targeting capability.1e,^4^–^6^

Cationic lipids are made up of a positively charged head group with a linker to a hydrophobic component. Since DOTMA was first described, analogues with ether or ester linkages to the glycerol skeleton, alternative alkyl chain groups and functionalized head groups have been reported. Examples include the diester DOTAP,^7^ diester DORI and corresponding diether DORIE, and DMRIE (Fig. 1).^3^,^8^ Such hydroxyalkyl cytofectins were reported to be more active than DOTMA.^8^ It was postulated that they were particularly effective, either because the hydroxyl group was able to increase the liposome interaction with DNA or cellular membranes, or because it was able to stabilize the cationic lipid : DOPE bilayer structure, through charge neutralization and/or hydrogen bonding.^8^ Others have also incorporated hydroxyalkyl chains onto quaternary amine species with varying results.^9^,^10^

Alterations to the alkyl chain, including factors such as chain saturation, double bond geometry, and chain length have been shown to be important in lipoplex and lipopolyplex formulations.^11^–^17^ In addition, other head groups such as aminopropyl groups, and short polyethylene glycol (PEG) shielded glycerol-based lipids with non-cleavable and cleavable functionalities have been reported.^14^,^16^–^20^ Studies established that shielding nanoparticles with long PEG-lipid conjugates, while enhancing circulation times and reducing vesicle aggregation, resulted in lower transfections efficiencies.^16^ However, the use of short *n*-ethylene glycol (*n*-EG) moieties attached to lipid headgroups still gave improved LPD particle stabilities but enabled nanoparticle targeting and good *in vitro* and *in vivo* transfection efficiencies.^16^–^20^

Addition of the neutral co-lipid DOPE is often found to enhance transfection levels, and is attributed to improved endosomal membrane fusion, due to the presence of DOPE changing the bilayer L_α_ phase into the inverted hexagonal H_II_ phase, promoting endosomal escape.^3^,^8^,^9^ An enhanced transfection however is not always observed, for example in formulations of DOPE with βAE-DMRIE.^21^

**Scheme 1:**
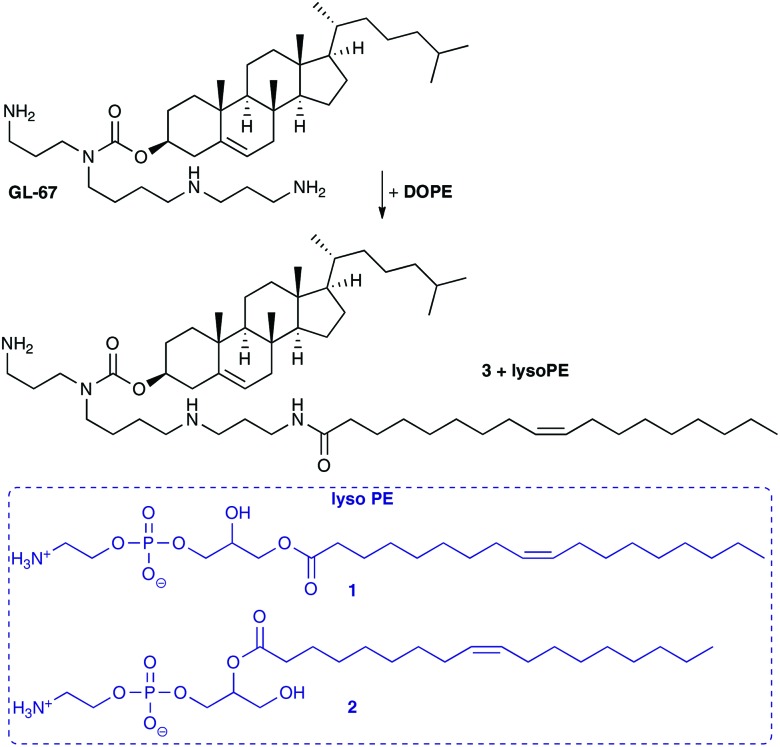
Acyl transfer from DOPE to give lyso-PE (**1** and **2**) and amidation products of GL-67 such as **3**.^23^

Interestingly, some groups have reported that on storage, an acyl chain transfer reaction can occur from an acyl chain on the DOPE lipid to the terminal primary amine of the co-formulated lipid.^22^,^23^ It was noted in experiments with the cholesterol lipid GL-series, such as GL-67, and DOPE (Scheme 1) co-formulated in aqueous media, that transfection levels diminished by 10–20 fold.^22^ When the GL-lipids were formulated with other co-lipids this was not the case and transfections levels were enhanced. HPLC analysis of mixtures of GL-67 and DOPE revealed the formation of lyso-PE (**1** and **2**) and amidated GL-67 such as **3**. Analysis of the reaction revealed a 6% conversion after 5 h in hydrated mixtures, but very low levels when stored as dried lipid films.^22^ Lyso-PE (**1** and **2**) and **3** were tested in transfection experiments which confirmed they were ineffective transfection reagents, and when lyso-PE was added to GL-67/DOPE formulations, lower levels of transfection were also observed.^22^ The acyl chain transfer effect was also highlighted with an analogue of GL-67.^22^ A similar acyl chain transfer reaction has been noted with diester lipids possessing a primary amine head group, where some fluorinated analogues were less prone to acyl transfer.^9^ It is unclear whether the acyl transfer reaction from DOPE to cationic lipids possessing a terminal primary amine group is a general phenomenon, but reduced levels of transfection were observed in the compounds studied, presumably due to reduced capacity of the lipids to be protonated and induce endosomal breakdown as well as changes to the molecular architecture of the nanoparticles formed.

**Scheme 2:**
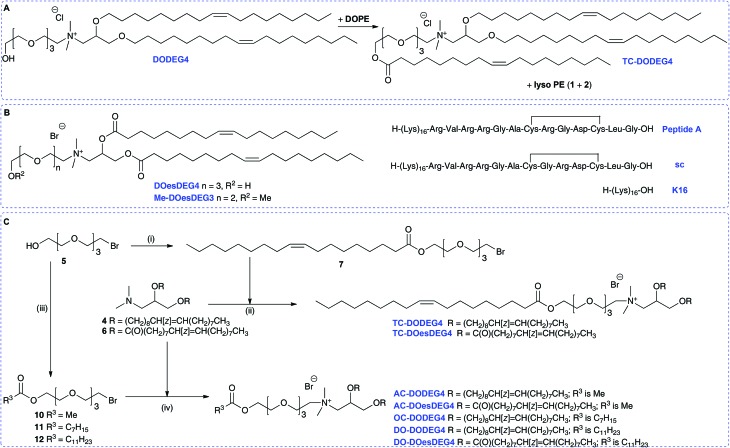
(A) Formation of TC-DODEG4 from DODEG4 and DOPE. (B) Structures of lipids DOesDEG4 and Me-DOesDEG3, Peptide **A**, **sc** and **K16**. (C) Synthesis of TC-lipids. Reagents and conditions: (i) oleic acid, DMAP, DIC, CH_2_Cl_2_, 54%; (ii) for TC-DODEG4 acetone, 90 °C, 36%; for TC-DOesDEG4 acetone, 80 °C, 11%; (iii) **5**, DMAP, py, Ac_2_O, for **10** 36%; **5**, H_15_C_7_COCl, DMAP, Et_3_N, for **11** 52%; **5**, H_23_C_11_COCl, DMAP, Et_3_N, for **12** 54%; (iv) acetone, 90 °C, AC-DODEG4 30%, AC-DOesDEG4 55%, OC-DODEG4 60%, DO-DODEG4 19%, DO-DOesDEG4 52%.

Here we report that in the course of our studies on gene delivery using cytofectins possessing hydroxyl terminated short *n*-EG head groups, it was noted that when lipid DODEG4 was co-formulated as vesicles with DOPE and stored for periods of up to several months before peptide was added to prepare an LPD, a trichain (TC) lipid TC-DODEG4 was formed that gave improved levels of LPD transfection (Scheme 2A). This transacylation of an acyl chain from DOPE to the terminal hydroxyl group occurred in an analogous fashion to that observed with GL-67 (Scheme 1).^22^ Therefore, several hydroxyl terminated short *n*-EG lipids were prepared and the corresponding TC-lipids, then LPD transfection experiments and biophysical characterisations performed to understand this change in efficacy. The transacylation reaction is of importance because there are many hydroxyl, *n*-EG and PEG terminated lipids in use that when formulated with DOPE may be affected by this process. In addition, this may lead to the identification of new improved compounds for delivery applications.

## Results and discussion

### Synthesis and lipid transacylation

**Fig. 2:**
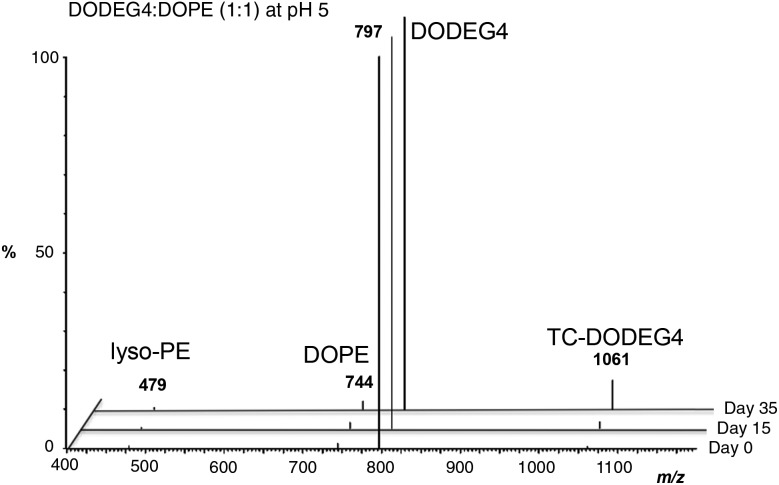
Formation of TC-DODEG4 and lyso-PE (**1** and **2**) from DODEG4 and DOPE, at pH 5 monitored by LC-MS.

We have previously reported the use of the lipid, DODEG4, a glycerol-based diether lipid possessing a short *n*-EG headgroup for applications in LPD targeted delivery.^17^ The lipid has been used with the co-lipid DOPE, to provide a short *n*-EG layer to shield nanoparticles for enhanced stabilities, while still enabling peptide targeting to be achieved.^17^,^20^ Interestingly when liposomal formulations of DODEG4 : DOPE were stored at 4 °C for several months, then used in the preparation of LPDs containing peptide **A** (Scheme 2B) and transfections examined in a range of cell types including HAE and NIH3T3 cells, improved transfection efficiencies were noted when compared to LPDs prepared from freshly made vesicles. MS-analysis revealed that a transacylated trichain lipid, TC-DODEG4, had formed (Scheme 2A). Consequently, the chemical stability of the lipid, DODEG4 (in the presence or absence of DOPE) was investigated, over a period of one month, using TLC analysis and LC-MS. As expected, when the lipids were stored in an organic solvent (CHCl_3_) no changes in lipid structure were observed by LC-MS. When DODEG4 was formulated, in the absence of DOPE, as liposomes dispersed in water at pH 5 and pH 7.5, no new compounds were again formed. For liposomal formulations made using a 1 : 1 molar ratio of DODEG4 : DOPE dispersed in water at pH 7 however, the slow formation of TC-DODEG4 (*m*/*z* 1061) together with lyso-PE (**1** and/or **2**) (*m*/*z* 479) was observed (Fig. S1 ESI†) as a consequence of transfer of an oleoyl chain from DOPE. When the liposomes containing DODEG4 : DOPE (1 : 1) were dispersed in water at pH 5, the transacylation to give TC-DODEG4 and lyso-PE (**1** and **2**) was more pronounced (Fig. 2).

To probe the behaviour of these dichain (DC) and trichain (TC) lipids in detail, several structurally related terminal *n*-EG DC and the corresponding TC lipids were prepared for investigation and application in LPD gene transfections (Scheme 2 and Table 1, referred to as Group 1 lipids). DODEG4 (Scheme 2A) was synthesized as previously reported, from the diether **4** and 4-EG bromide **5**. A diester analogue DOesDEG4 (structure in Scheme 2B) was similarly prepared *via* the quaternisation of **6** 8 with **5**. The corresponding TC lipids TC-DODEG4 and TC-DOesDEG4 were synthesized by the quaternisation of **4** and **6** with the 4-EG oleoyl ester **7**, prepared from oleic acid and bromide **5** (Scheme 2C).

**Table 1:** Summary of the dichain and trichain lipid prepared (Group 1 lipids) and dichain lipids capped with different ester chain lengths (Group 2 lipids)

DOTMA-based ether series	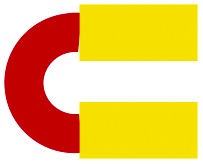	DOTAP-based ester series	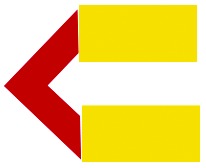
**Group 1 lipids**			
DODEG4	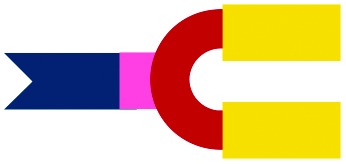	DOesDEG4	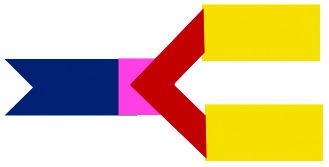
TC-DODEG4	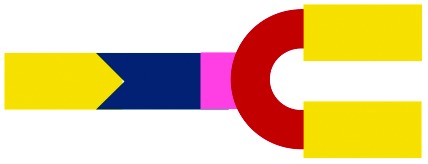	TC-DOesDEG4	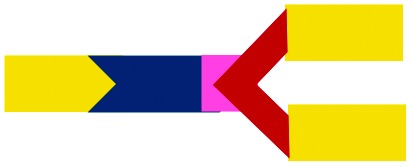
		Me-DOesDEG3	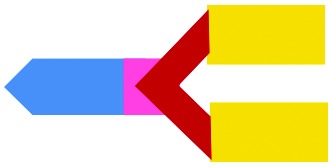
DOSEG3	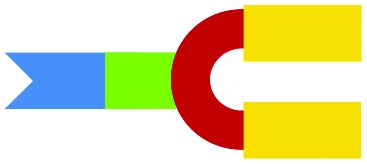	DOesSEG3	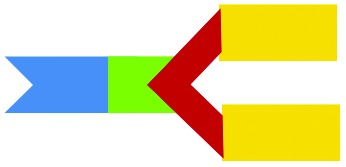
TC-DOSEG3	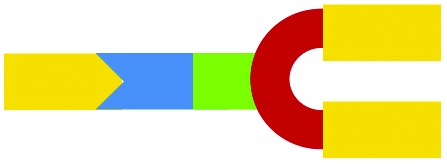	TC-DOesSEG3	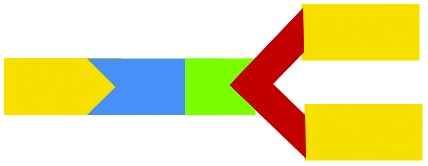
		Me-DOesSEG3	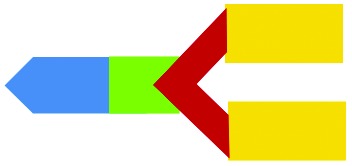

**Group 2 lipids**			
AC-DODEG4	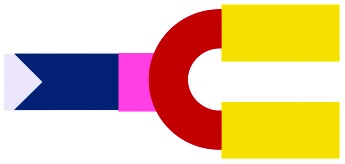	AC-DOesDEG4	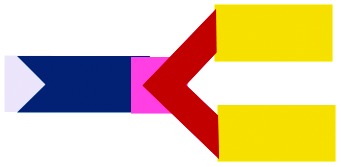
OC-DODEG4	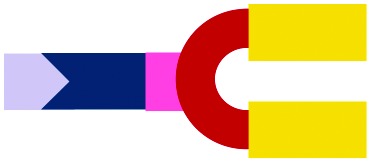		
DO-DODEG4	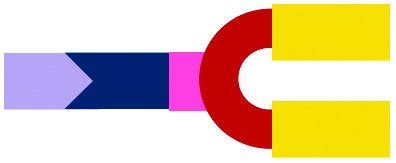	DO-DOesDEG4	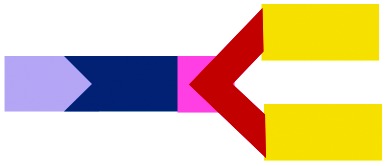
TC-DODEG4	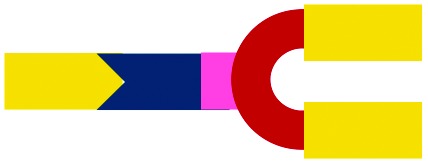	TC-DOesDEG4	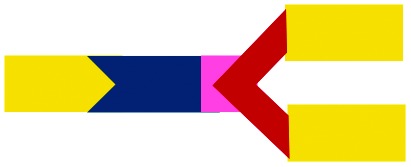

Compounds TC-DODEG4 and TC-DOesDEG4 were formed in 36% and 11% yield respectively, reflecting the general difficulties in purifying the lipids. In addition, the di- and tri-esters DOesDEG4 and TC-DOesDEG4 showed a tendency to hydrolyse when undergoing purification on silica. A methoxy-capped analogue of DOesDEG4, Me-DOesDEG3 (structure in Scheme 2B), was also synthesized from amine **6** and 1-(2-bromoethoxy)-2-(2-methoxyethoxy)ethane^24^ as this is a lipid that cannot undergo transacylation with DOPE (Scheme 2).

**Scheme 3:**
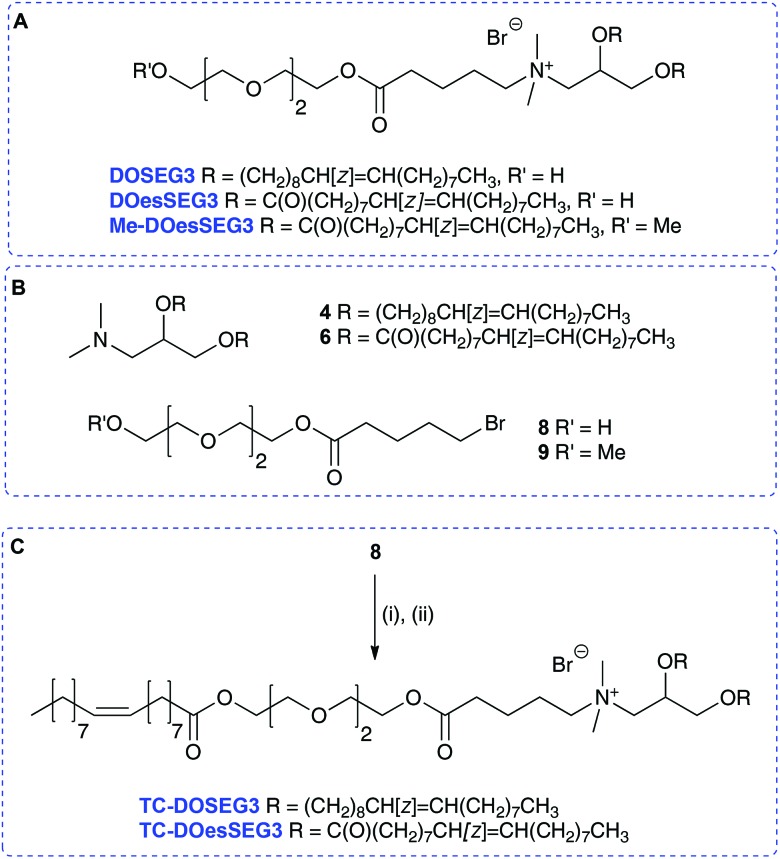
(A) Structures of lipids DOSEG3, DOesSEG3, Me-DOesSEG3. (B) Structures of substrates **4**, **6**, **8**, **9**. (C) Synthesis of TC-DOSEG3 and TC-DOesSEG3. Reagents and conditions: (i) oleic acid, DMAP, DIC, CH_2_Cl_2_, 75%; (ii) for TC-DOSEG3, **4**, acetone, 90 °C, 11%; TC-DOesSEG3, **6**, acetone, 90 °C, 45%.

A further set of lipids were prepared based upon a previously reported short *n*-EG terminated lipid DOSEG3, effective in targeted LPD transfections (Table 1; Group 1 lipids).^19^ This lipid possesses an endosomal esterase cleavable functionality to release the *n*-EG group to enhance endosomal breakdown, and was prepared as previously described *via* the quaternisation of **4** and the *n*-EG ester-bromide **8**.^19^ In addition to DOSEG3, the novel dichain diester analogue DOesSEG3 was prepared, which may have greater biodegradability, and the corresponding methoxy capped analogue Me-DOesSEG3 (structures in Scheme 3A). They were synthesized by the reaction of bromides **8** 19 and **9** 19 with the tertiary amine **6**, in 78% yield (**4**, **6**, **8**, **9** are in Scheme 3B).

Two trichain lipids were also synthesized based on DOSEG3 and DOesSEG3, TC-DOSEG3 and TC-DOesSEG3. The *n*-EG bromide **8** was coupled to oleic acid in 75% yield, and the product reacted with the corresponding tertiary amines **4** and **6** to give TC-DOSEG3 and TC-DOesSEG3 (Scheme 3C). Again, purification of the final TC-lipids was not straightforward, and compounds were purified at least twice by silica column chromatography.

In addition to this first group of cytofectins (Group 1 lipids) comprised of traditional dialkenyl chains and the new TC series, a second group of cytofectins (Group 2 lipids) based on DODEG4, but capped with acyl chains of different lengths, were prepared to probe the effect of the length of the third chain. This type of the TC lipid was denoted by the length of the third acyl chain, namely either an acetyl (AC–), octanoyl (OC–) or a dodecanoyl (DO–) chain.

Analogues of DODEG4 and DODesDEG4 with acetyl groups were synthesised using analogous routes to those described above. The 4-EG bromide **5** was reacted with acetic anhydride to give **10** (Scheme 2C), which was reacted with amines **4** and **6** to give AC-DODEG4 and AC-DOesDEG4 in 30% and 55% yield, respectively. Compound OC-DODEG4, with an intermediate terminal octanoyl chain length, was prepared similarly from **5** and octanoyl chloride to give bromide **11**, which was then reacted with **4** to give the lipid in 60% yield. Dodecanoyl chloride was also used to make bromide **12**, which was reacted with **4** and **6** to give DO-DODEG4 and DO-DOesDEG4 (Scheme 2C and Table 1).

### Lipid : Peptide : DNA transfections

Transfections were performed in rat neuroblastoma B104 cells, using LPDs prepared from vesicles containing the lipids, in the presence of a 1 : 1 molar ratio of DOPE (or 1,2-dioleoyl-*sn-glycero*-3-phosphocholine (DOPC) in the case of DO-DODEG4 and DO-DOesDEG4 since it was impossible to formulate these lipids into vesicles in the presence of DOPE) and peptide **A**. The cyclic sequence -CRGDC-LG of peptide **A** was chosen because it binds to α_v_ integrin receptors and we have previously demonstrated that LPDs formulated using peptide **A** mediate recognition and cell-specific uptake in neuroblastoma cells *in vitro* and *in vivo*.^19^,^20^ Control LPD formulations were also prepared using a scrambled sequence K_16_RVRRGA-CGRDC-LG (**sc**) which should not be recognised by the α_v_ integrin receptors.

**Fig. 3:**
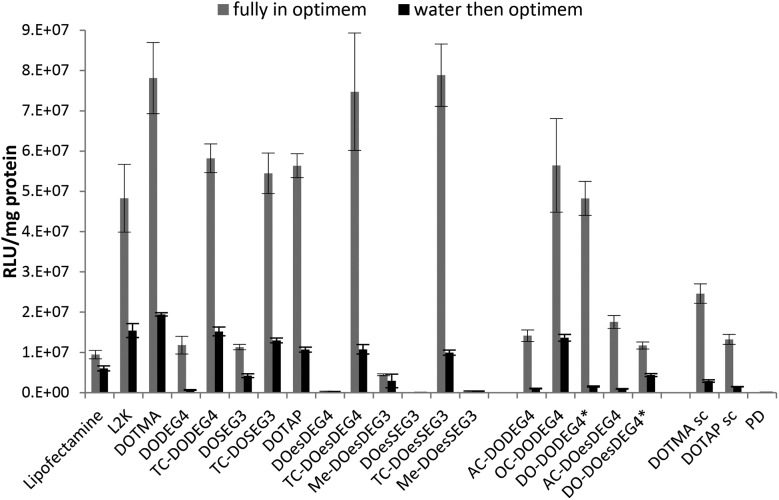
*In vitro* transfection efficiency (expressed as RLU mg^–1^ protein) obtained in B104 cells after exposure to LPDs prepared at a lipid : peptide : DNA charge ratio of 0.25 : 6.5 : 1 in either Opti-MEM (grey bars) or in 25% v/v water then diluted with Opti-MEM (black bars). Cationic lipid used to prepare the LPDs shown on the abscissa were formulated with DOPE or *DOPC. PDs prepared at a peptide : DNA charge ratio of 6.5 : 1, and LDs of Lipofectamine and L2K prepared at weight ratios of 5 : 1 and 4 : 1, respectively. Peptide and DNA used in all cases were peptide **A** and gWiz plasmid DNA (pDNA), respectively. Data is the mean of three measurements ± standard deviation.

The LPDs were prepared either in Opti-MEM or in water and diluted by 1 in 4 with Opti-MEM, and the results are shown in Fig. 3. The preparation of LPDs in Opti-MEM was to allow a comparison with other researchers who have used this protocol. All LPDs tested were formulated at lipid : peptide : DNA (L : P : D) charge ratios of 0.25 : 6.5 : 1 (equivalent to a weight ratio in the range 0.7–1 : 4 : 1, the exact weight ratio depending upon the particular cationic lipid used) since this was the charge ratio found to produce the highest transfection results during a preliminary screen using B104 cells (Fig. S2, ESI†). The preliminary screen (B104 cells) with LPDs were made from cationic vesicles containing DOPE and either DODEG4, TC-DODEG4, DOSEG3 and TC-DOSEG3 together with peptide **A** at final L : P : D weight ratios of 0.5 : 4 : 1, 1 : 4 : 1, 2 : 4 : 1 and 3 : 4 : 1. The level of transfection achieved using LPDs with the higher lipid weight ratio of 3 : 4 : 1 was ∼20% of that obtained for the lower lipid ratios of 1 : 4 : 1 or 0.5 : 4 : 1. LPDs prepared using these lower weight ratios produced similarly high levels of transfection. Transfections were repeated on more than one occasion and while differing absolute levels of transfection were obtained on different occasions the order of transfection efficiencies were consistent between experiments.

As can be seen in Fig. 3, regardless of whether the LPDs were initially prepared in Opti-MEM or water, the LPDs made at a L : P : D charge ratio of 0.25 : 6.5 : 1 using the TC lipids of Group 1 exhibited superior transfection abilities to those prepared using DC lipids. Increases in transfection ranged from a 5-fold in the case of LPDs containing DOSEG3 and DODEG4 to a 200 and 300-fold increase in the case of LPDs prepared using DOesSEG3 and DOesDEG4, respectively, suggesting that the presence of a short ethylene glycol chain had a detrimental effect on transfection *in vitro*. Although the acetyl and, to a lesser extent, the methoxy-capped analogues exhibited a slight improvement in transfection efficiency, this increase was not as high as that obtained when using the corresponding oleoyl containing TC analogues. In contrast the OC- and DO-derivatives of DODEG4 (Group 2 lipids), gave comparable transfection levels to the LPDs prepared using TC-lipids. Note that the DO-derivative of DOesDEG4 exhibited a low level of transfection, less than that achieved with AC-DOesDEG4. LPDs prepared using DOTMA and DOTAP exhibited similar transfection efficiencies to the TC- and OC-lipids, suggesting that the presence of a third acyl chain negates the detrimental effect of the short ethylene glycol chain on *in vitro* transfection.

For the purposes of comparison, polyplexes (PD) of peptide **A**: DNA (prepared at a charge ratio of 6.5 : 1) and lipoplexes of Lipofectamine and L2K (prepared at lipid : DNA weight ratios of 5 : 1 and 4 : 1, respectively) were also examined for their transfection ability (Fig. 3). The polyplex demonstrated an extremely poor transfection efficiency, a result in line with previous studies^15^ while the use of complexes prepared using Lipofectamine and L2K generally resulted in similar or lower transfection levels and higher toxicities than those achieved with LPDs prepared using the TC lipids, some DC lipids, DOTMA or DOTAP. Transfection experiments were performed on at least three separate occasions and the trends observed (Fig. 3) were reproducible. Scrambling of peptide **A** resulted in a 70–75% decrease in transfection confirming selective targeting of the B104 cells by peptide **A**. Complexes prepared in the absence of a targeting peptide *i.e.* TC lipid : DNA (LDs) have recently been published in a separate study.^25^ The LDs prepared from TC lipids also showed superior transfection efficiencies compared to their DC equivalents, however the absolute transfection efficiency of the LDs was lower by around an order of magnitude.^25^

LPDs prepared fully in Opti-MEM generally showed better transfection efficiency than those prepared in 25% v/v water, followed by dilution with Opti-MEM before applying onto the cells. The reasons are not currently understood but could be due to differences in the size of the complexes when prepared in Opti-MEM as opposed to water (particle size of the complexes prepared fully in Opti-MEM were larger than those prepared in water), and/or the looser association of DNA with the peptide and the lipid when formulated in Opti-MEM due to the presence of electrolyte that partially neutralises the cationic charge on the peptides and lipid. However, similar trends in transfection were observed regardless of the Opti-MEM or water content, the only exception being DO-DODEG4 which exhibited a far lower transfection efficiency when prepared in water rather than Opti-MEM.

An assay to determine the percentage protein in each well compared to blank (untreated) cells was simultaneously performed with every transfection experiment to give an indication of any toxicity caused by the various formulations (Fig. S3, ESI†). Significantly, the cells exposed to the LPD formulations generally exhibited greater than 80% protein content, suggesting that the formulations caused little or no toxicity. The only cells exhibiting a lower protein content of ∼60% were those exposed to LPDs prepared using the TC-DOesSEG3 and then only when the LPDs were formulated in water and diluted with Opti-MEM, *i.e.* not fully prepared in Opti-MEM. Notably, both Lipofectamine and L2K exhibited considerable toxicity towards the cells as evidenced by the ∼65–70% and 35–55% protein contents obtained when in the cells exposed to LDs prepared either fully in Opti-MEM or in water and diluted with Opti-MEM, respectively.

In addition, some transfection studies were performed to investigate the influence of the neutral co-lipid lyso-PE on the effectiveness of DNA delivery. In these studies, lyso-PE was used in the place of DOPE during the preparation of LPDs. Previous reports have suggested that replacing DOPE with lyso-PE in cationic liposome formulations reduces the effective volume of the lipid hydrophobe, thus lowering the curvature stress of the liposomes which in turn, results in liposome formulations that cannot effectively induce cell–cell fusion.^26^ It was possible therefore that the presence of lyso-PE in an LPD formulation might lead to a reduction in transfection efficiency. A screen of LPDs prepared using an equimolar amount of TC-lipid and lyso-PE in place of DOPE to mimic the fact that lyso-PE would be formed during the transacylation process (Fig. S4, ESI†) showed that LPDs containing both TC-lipid and lyso-PE tended to be similar to or slightly more effective at transfecting B104 cells, with no significant detrimental effect on toxicity as assessed by determination of the percentage protein in each well. This observation supports the hypothesis that it is the TC-lipid and not the lyso-PE formed that is the beneficial transfection agent in these studies.

A series of biophysical experiments were then performed to understand the reasons for the enhancement in transfection observed with the Group 1 and Group 2 TC lipids. Of particular interest was whether the use of a TC lipid resulted in a different LPD structure than has been previously observed for LPDs formed by DC lipids,^5^ where biophysical studies showed that the lipopolyplexes consisted of a core of DNA complexed with peptide surrounded by a bilayer of lipid. Studies using LDs prepared from TC lipids showed a major structural re-organisation of the lipid bilayer, suggesting that the third chain may fold over and create inter-digitated structures.^25^ It is not known, however how the trichain lipids behave in ternary systems in the presence of a targeting peptide.

### Size and zeta potential

**Fig. 4:**
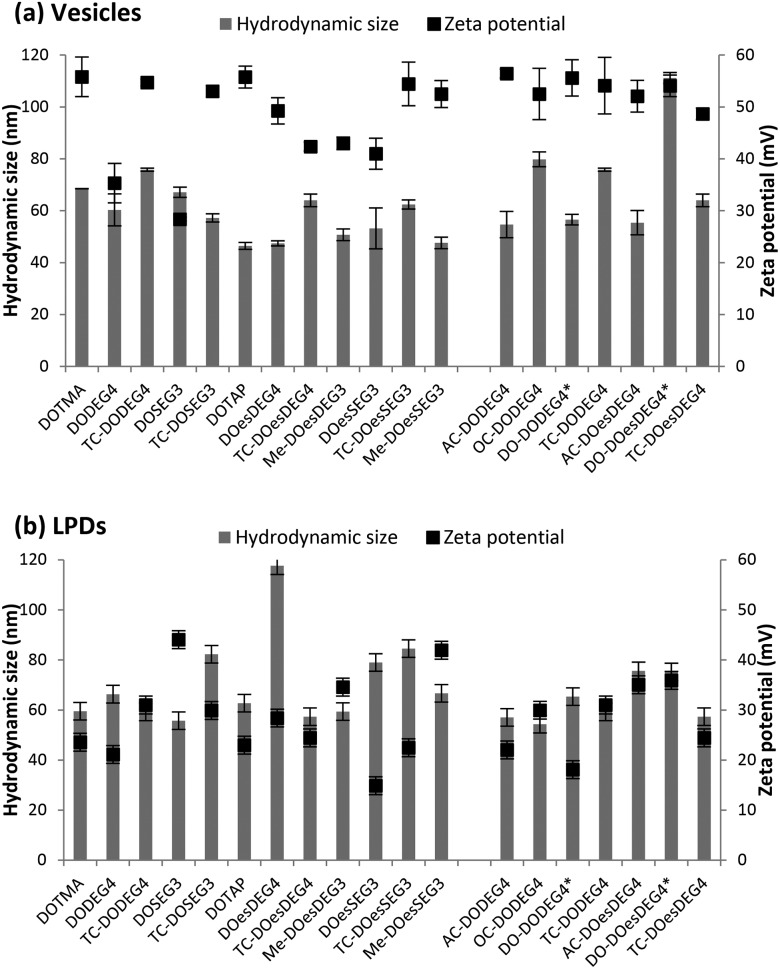
The apparent hydrodynamic size (grey bars) and zeta potential (black squares) of (a) cationic lipid vesicles prepared using at 1 : 1 molar ratio of DOPE or *DOPC and (b) LPD complexes prepared using cationic lipid vesicles, peptide **A** and pDNA at L : P : D charge ratios of 0.25 : 6.5 : 1. Measurements were performed at a cationic lipid concentration of 5 μg mL^–1^ for vesicles and a DNA concentration of 1 μg mL^–1^ for LPDs. Data is the mean of three independent measurements ± standard deviation.

The apparent hydrodynamic size (assuming spherical particles) and zeta potential of the cationic lipids formulated in water were determined, either as vesicles with DOPE at 1 : 1 molar ratio or as LPDs at lipid : peptide **A** : gWIZ plasmid (pDNA) charge ratios of 0.25 : 6.5 : 1, measured at cationic lipid concentrations of 5 μg mL^–1^ and 1 μg mL^–1^, respectively (Fig. 4). With the exception of the vesicles formed using the Group 2 lipid, DO-DOesDEG4, which were ∼110 nm, all vesicles prepared using Group 1 and Group 2 lipids exhibited sizes in the range 45–80 nm, and polydispersities of between 0.1–0.3 (Fig. 4a and b). The size of the DC-containing vesicles, including those prepared using methyl capped lipids, tended to be at the lower end of the size range suggested that these vesicles are predominately unilamellar in nature, although the presence of a small population of multilamellar structures cannot be dismissed.

By way of comparison, vesicles prepared using the longer acyl chain TC lipids (*i.e.* a third acyl chain of C_8_ and above) tended to be slightly larger than their DC counterparts. The observation that the longer acyl chain TC lipids, when in combination with DOPE, formed vesicles was unexpected. Indeed, because of the relatively large hydrophobic regions of both the TC lipids containing an acyl chain of C_8_ and above and DOPE, mixtures of such lipids with DOPE had been expected to promote the formation of reverse structures such as reverse hexagonal phases.

All vesicles (prepared from Group 1 and Group 2 lipids) exhibited a zeta potential of at least ∼30 mV (DOSEG3-containing vesicles), although the typical value was in the range 40–50 mV, indicating that the vesicles should exhibit a high stability (Fig. 4a and b).

The apparent hydrodynamic sizes recorded for the LPDs were similar, although slightly larger than those obtained for the vesicles at between 55–115 nm, with most being less than 80 nm, with polydispersities in the range 0.1–0.4. Significantly, the zeta potentials measured for the LPDs were still positive, albeit lower than the vesicles at 15–45 mV, suggesting that the LPDs may be less stable. Interestingly, no correlation between the size and zeta potential of the LPDs and the structure of the cationic lipids used to prepare the complexes was evident.

### Transmission electron microscopy

**Fig. 5:**
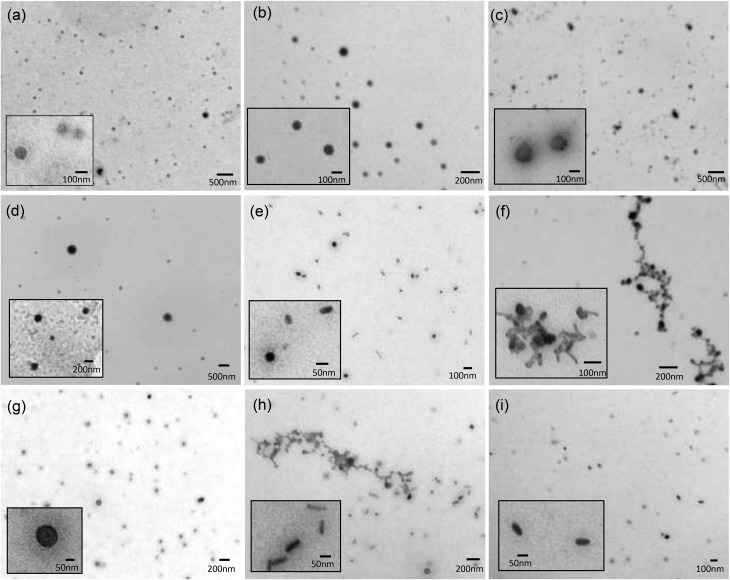
Transmission electron micrographs of lipid vesicles composed of a molar ratio of DOPE and cationic lipid (a) DODEG4, (b) TC-DODEG4, (c) DOSEG3 and (d) TC-DOSEG3; and LPDs formulated with peptide **A** and pDNA at charge ratios of 0.25 : 6.5 : 1 using (e) DODEG4, (f) TC-DODEG4, (g) DOSEG3 and (h) TC-DOSEG3; and (i) PD prepared from peptide **A** : pDNA complexes at 6.5 : 1 charge ratio. All samples were stained with a 4% w/v uranyl acetate solution.

Transmission electron microscopy (TEM) in combination with negative staining using a 4% w/v solution of uranyl acetate was used to examine the structure of the vesicles (co-formulated at a 1 : 1 molar ratio with DOPE) and LPDs (prepared from cationic vesicles, peptide **A** and gWIZ plasmid pDNA at a 0.25 : 6.5 : 1 charge ratio) made using a selection of Group 1 DC and TC lipids, namely DODEG4, TC-DODEG4, DOSEG3 and TC-DOSEG3 (Fig. 5). Significantly, although some polydispersity in size was apparent, the aggregates observed were predominately spherical in nature, irrespective of whether the cationic lipid used was a DC or TC, supporting the presence of vesicular structures (Fig. 5a–d). Although vesicles are not visualised well using TEM, since the sample is dehydrated upon staining, TEM was performed in order to rule out the formation of other aggregate types, since to our knowledge this is the first time that LPDs have been prepared from lipids containing three long (C18:1) acyl chains.

In contrast to the spherical nature of the vesicles, the LPD formulations (Fig. 5e–h) consisted of a mixture of spherical and rod-shaped particles, with no apparent visible internal structure: an observation in agreement with LPD formulations reported previously.^15^,^27^ This observation is probably a consequence of the fact that the ratio of lipid : peptide : DNA was not fully optimised in the present study. Furthermore, there were some observable differences between the LPDs prepared using the DC and TC lipids in that the LPDs formulated from the DC lipids were relatively monodisperse with individual particles of between 50–100 nm in diameter, while the LPDs prepared using the TC lipids, although similar in shape were more polydisperse and displayed some particle aggregation (Fig. 5f and h). It is not known at present whether these aggregates are due to drying and/or surface effects, although it should be noted that the size of the LPDs measured by dynamic light scattering suggested that when in solution the LPDs are not (significantly) aggregated. Furthermore, the LPDs were prepared freshly for the TEM study.

### Small angle neutron scattering

**Fig. 6:**
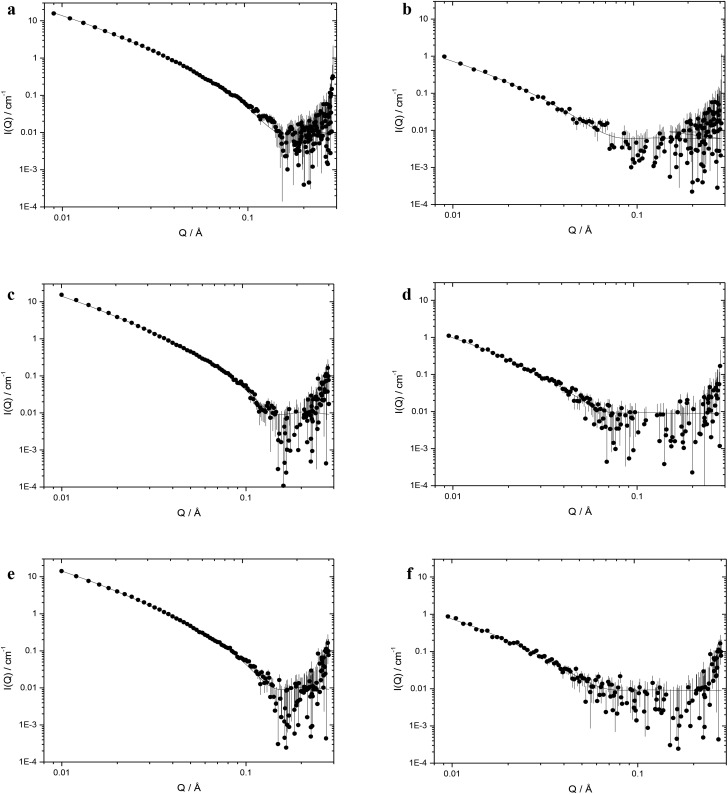
Small angle neutron scattering data (dots) recorded at 298 K and the best fit to the data (solid line) obtained using the mixed sheet and stack model for freshly prepared vesicles prepared from DOPE and 1 : 1 molar ratio of cationic lipid: (a) the dichain lipid, DOesDEG4, (c) the corresponding trichain lipid TC-DOesDEG4, and (e) their methoxy capped dichain counterpart Me-DOesDEG4, at a cationic lipid concentration of 1 mg mL^–1^ and LPDs prepared from these vesicles containing (b) DOesDEG4, (d) TC-DOesDEG4, and (f) Me-DOesDEG3, peptide **K16** at a ctDNA concentration of 0.0625 mg mL^–1^ and a L : P : D charge ratio of 0.5 : 6 : 1.

Table 2 shows the structural parameters obtained through model fitting of small angle neutron scattering (SANS) data obtained for cationic vesicles containing 50 mol% DOPE (at a cationic lipid concentration of 1 mg mL^–1^) and LPDs prepared at a L : P : D charge ratio of 0.25 : 6.5 : 1 (containing 0.175 mg mL^–1^ of **K16** peptide and 0.0625 mg mL^–1^ of ctDNA) as single, flat sheets (or bilayers) with or without the presence of stacks thereof. The data for the corresponding vesicles and LPDs formed from DOTMA and DOTAP are also included in Table 2. A representative selection of the SANS profiles recorded for the cationic lipid : DOPE vesicles (those made from DOesDEG4, TC-DOesDEG4 and Me-DOesDEG4) together with the LPDs (prepared using ctDNA and **K16** at L : P : D charge ratios of 0.25 : 6.5 : 1) are shown in Fig. 6.

**Table 2:** Structural parameters obtained through model fitting of small angle neutron scattering data of freshly prepared cationic vesicles containing 50 mol% DOPE at a cationic lipid concentration of 1 mg mL^–1^, and LPDs prepared at a L : P : D charge ratio of 0.25 : 6.5 : 1 and containing **K16** peptide at a DNA concentration of 0.0625 mg mL^–1^

Cationic lipid	*L* (nm)	*D* (nm)
DOTMA	V 4.2 ± 0.1	V 6.2 ± 0.1
	LPD 4.2 ± 0.3	LPD 6.5 ± 0.1
DODEG4	V 4.1 ± 0.1	ND
	LPD 6.3 ± 0.2	ND
TC-DODEG4	V 4.2 ± 0.1	V 5.9 ± 0.1^*a*^
	LPD 9.5 ± 0.3	LPD 11.8 ± 0.1
DOSEG3	V 4.1 ± 0.1	ND
	LPD 6.4 ± 0.2	ND
TC-DOSEG3	V 4.0 ± 0.1	V 5.9 ± 0.1^*a*^
	LPD 6.8 ± 0.2	LPD 11.6 ± 0.1
DOTAP	V 4.2 ± 0.1	V 6.2 ± 0.1
	LPD 4.2 ± 0.3	LPD 6.5 ± 0.1
DOesDEG4	V 3.9 ± 0.1	ND
	LPD 6.9 ± 0.3	ND
TC-DOesDEG4	V 4.1 ± 0.1	V 5.8 ± 0.3
	LPD 7.8 ± 0.4	ND
Me-DOesDEG4	V 4.0 ± 0.1	ND
	LPD 7.6 ± 0.5	ND
DOesSEG3	V 4.1 ± 0.1	V 6.0 ± 0.1
	LPD 7.3 ± 0.3	ND
TC-DOesDEG3	V 4.1 ± 0.1	ND
	LPD 9.1 ± 0.4	LPD 13.0 ± 0.1
Me-DOesDEG3	V 4.0 ± 0.1	ND
	LPD 6.6 ± 0.3	ND
AC-DODEG4	V 3.9 ± 0.1	ND
OC-DODEG4	V 3.8 ± 0.1	V 5.9 ± 0.1
DO-DODEG4	V 3.9 ± 0.1	ND

^*a*^Here *M* (the number of stacks in a multilayer structure) = 5.

Regardless of the presence and length of a third chain, all the cationic vesicles examined in the present study were well modelled assuming the presence of single, flat sheets (*i.e.* unilamellar vesicles) and, where necessary, with the addition of stacks (*i.e.* multilamellar vesicles). The size of all the cationic vesicles in the present study are too large to be analysed by the SANS instrument used here as spherical structures. Indeed, the assumption of flat sheets is in line with the apparent hydrodynamic size of the vesicles made in earlier studies on related, DC cationic lipid-containing systems.^13^ As with the vesicles, it was possible to analyse the LPDs formed by all the Group 1 and Group 2 DC and TC lipids assuming the presence of sheets or bilayers of lipid. Significantly, in the absence of any data suggesting the presence of non-vesicular structures, it was reasonable to assume that the LPDs prepared using the TC lipids were, as with the LPDs prepared using DC lipids, composed of a lipid bilayer (or bilayers). This observation is supported by the results of the transmission electron microscopy study.

The SANS profiles of the vesicles and LPDs were measured on more than one occasion. This was important as vesicles prepared from the TC cationic lipids were expected to be less stable than their DC counterparts. Indeed, preliminary studies suggested that the amount of multilamellar structure present in the vesicles containing the DC lipids was consistently very low, while the amount in vesicular preparations containing a TC lipid tended to be higher depending on the age of the vesicles, particularly if the vesicles were over 1 month old. Significantly, however, the thickness of bilayer present in the TC vesicles did not change over the corresponding time period. When the LPDs were prepared from freshly made vesicles there was little evidence of the presence of any multilamellar structures, regardless of the nature of the lipid (DC or TC) used. However, if the LPDs were prepared from vesicles in which a significant amount of multilamellar structure was present, the LPDs exhibited a greater content of multilamellar structure. The data shown in Table 2 was acquired using freshly prepared vesicles and LPDs so the level of multilamellar content was very low.

Inspection of the values of the bilayer thickness, *L*, obtained for vesicles prepared from Group 1 and Group 2 DC and TC lipids in the presence of DOPE (Table 2) showed, perhaps somewhat surprisingly, that there was no change in the bilayer thickness of the lipids as a function of bond type (ester or ether), length of the *n*-EG linker and the absence/presence or length of a third chain. Furthermore, regardless of whether the cationic lipid was a DC or TC, the thickness of the water layer between the bilayers, when present, varied between 1.7 and 1.9 nm, suggesting a water layer between each bilayer of ∼2 nm, in line with previous results for other DC cationic vesicles.^13^

Interestingly the thickness of the bilayer was much greater when the cationic lipids were part of an LPD, with the thickness of the bilayers containing the DC lipids typically increasing by between ∼2.2 and 3.2 nm, while the increase in the bilayer containing the TC lipids was even larger in the range 2.8–5.3 nm. The methoxy capped lipids behaved more like the DC lipids exhibiting an increase in bilayer thickness of 2.6–3.6 nm. These results suggest a significant re-arrangement of the lipids in the presence of DNA, to accommodate the interaction of the cation with the negative charges on DNA, with the re-arrangement being greatest for the TC lipids. In contrast, DOTMA and DOPE and indeed previous SANS studies on vesicles and their corresponding LPDs prepared from cationic lipids without a short ethylene glycol chain did not exhibit any difference in bilayer thickness between the vesicles and LPDs.^15^

A limited SANS study was performed using LPDs prepared at a L : P : D charge ratio of 0.25 : 6.5 : 1 but containing 0.15 mg mL^–1^ of ctDNA and either 0.42 mg mL^–1^ of **K16** peptide or 0.6 mg mL^–1^ of peptide **A** to see the effect of concentration on the structure of the LPDs (Fig. S5, ESI† shows the vesicles and LPDs prepared from TC-DODEG). The thickness of the bilayer and lamellar *d*-spacing present in the LPDs prepared using the higher level of peptides **K16** and **A** were *L* = 9.3 ± 0.1 and *D* = 13.4 ± 0.1 (**K16**) and *L* = 8.8 ± 0.2 and *D* = 12.5 ± 0.1 (peptide **A**). These compared favourably to values of *L* = 9.5 ± 0.3 and *D* = 11.8 ± 0.1 nm when the LPDs were prepared at a ctDNA concentration of 0.0625 mg mL^–1^ and contained 0.175 mg mL^–1^ of **K16** peptide, suggesting that the concentration of LPDs had no effect under the present experimental conditions. Similar results were obtained for LPDs prepared using all of the cationic lipids tested.

### Circular dichroism

**Fig. 7:**
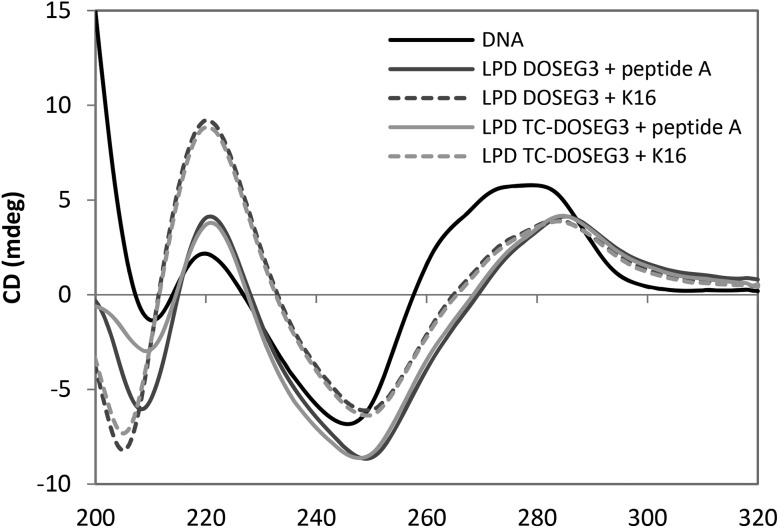
CD spectra in (*x*-axis nm) of ctDNA and LPDs prepared using DOSEG3 or TC-DOSEG3 in combination with either peptide **A** (green and pale blue) or **K16** (black and purple) at charge ratios of 0.25 : 6.5 : 1. Measurements were performed using a 0.5 cm path length at a final DNA concentration of 0.05 mg mL^–1^.

Circular dichroism (CD) spectra of LPDs prepared using representative Group 1 DC and TC lipids, DOSEG3 and TC-DOSEG3 respectively, either peptide **A** or **K16** peptide and calf thymus DNA (ctDNA) at a 0.25 : 6.5 : 1 charge ratio was measured and compared to a solution containing the same concentration of ctDNA (0.05 mg mL^–1^) (Fig. 7). All samples were prepared in D_2_O in order to increase the optical transparency of the solution at low wavelengths. Dynamic light scattering measurements of vesicles and LPDs made in D_2_O showed no difference in size suggesting that the replacement of H_2_O with D_2_O does not significantly alter the structure of the complexes. The CD spectrum obtained for ctDNA in solution (Fig. 7) showed that ctDNA adopts a B type conformation (namely a positive peak at ∼275 nm and a negative region between 260–230 nm) typical of DNA. A similar CD spectrum was obtained for pDNA (data not shown).

The CD spectra obtained for the DC and TC LPDs prepared using either peptide exhibited a red shift in the CD spectrum above ∼240 nm. Since there is no absorbance either from the peptide or the lipid above 240 nm (Fig. S6, ESI† shows the spectra of free peptide and lipid at the same concentration as used in the LPDs) this suggests that the conformation of ctDNA is altered when in the form of an LPD, most likely adopting a more condensed structure. Similar results have been obtained in previous studies using LPD formulations.^27^ Interestingly, while no difference in the extent of the red shift was observed with the type of lipid used, a greater red shift was seen in the LPDs containing peptide **A**. These results suggest that the ctDNA in the LPD complex is predominantly condensed by the peptide with relatively little contribution from the lipid present. This result also agrees with the LPD structure model described previously^5^ wherein the LPDs were proposed as having a core composed of predominately DNA : peptide surrounded by a bilayer of cationic lipid.

### Gel electrophoresis

**Fig. 8:**
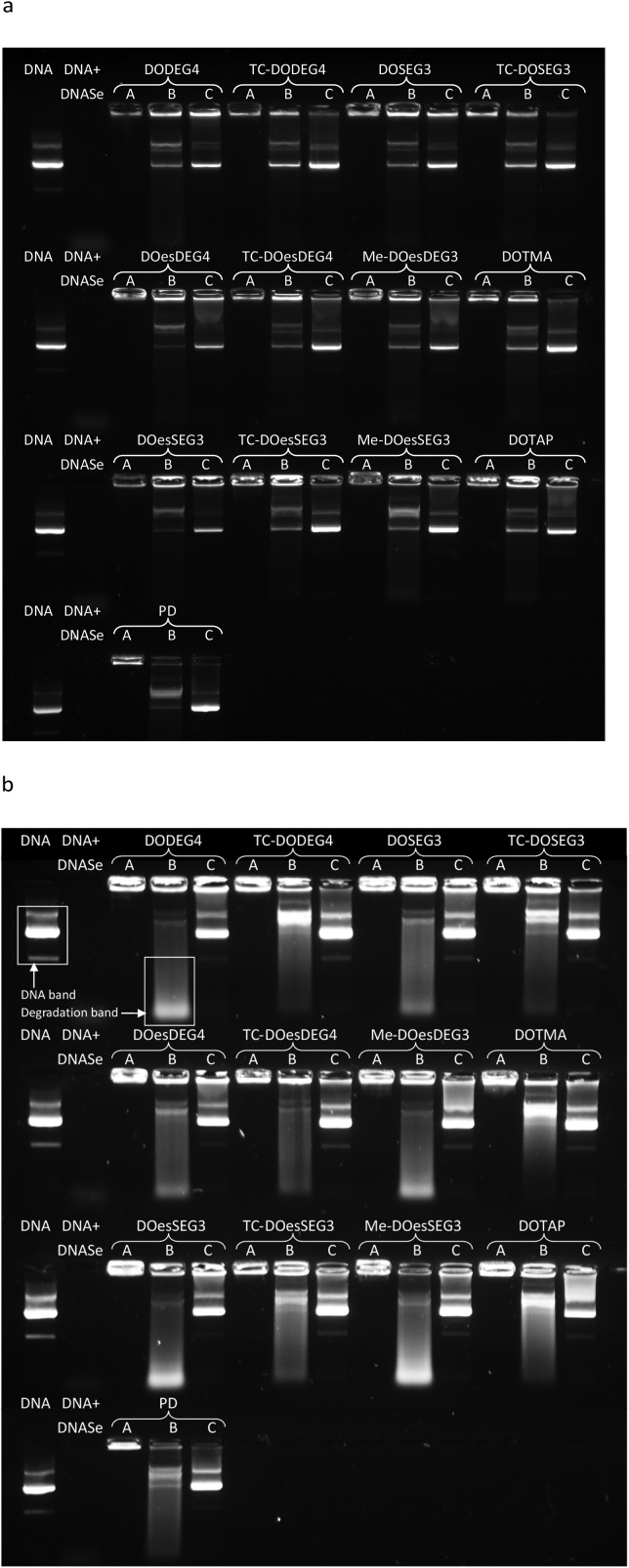
Gel condensation, protection and release assay of LPDs or PDs prepared at 0.25 : 6.5 : 1 or 6.5 : 1 charge ratios, respectively with peptide **A**, and gWIZ plasmid DNA (pDNA) at a concentration of 0.25 μg per 10 μL per well. LPDs were prepared using the DC and TC lipids and treated in either one of three ways: Lane A = untreated LPDs (showing DNA condensation); Lane B = LPDs treated with DNAse and pAsp (showing pDNA protection against enzymatic degradation); Lane C = LPDs treated with pAsp only (showing efficiency of DNA release from complex). Free (untreated) pDNA and pDNA treated with DNAse I were used as controls in each row. (a) Single strength (0.25 IU) DNAse I was used per well and (b) double strength (0.5 IU) DNAse I was used.

Gel electrophoresis was performed on LPDs prepared using the Group 1 and Group 2 DC and TC lipids, peptide **A** and pDNA at charge ratios of 0.25 : 6.5 : 1 and the corresponding polyplex (PD) to determine the extent of complexation of, enzyme protection afforded to, and release of DNA (Fig. 8, Lanes A, B and C, respectively). The extent of protection of pDNA in the LPDs against DNAse I, was measured using two amounts of DNAse I, namely single strength (1 IU per μg of pDNA) and double strength (2 IU per μg of pDNA) DNAse I (Fig. 8 Lane B). Double strength DNAse I was used to help elucidate trends.

It was evident that all the LPDs as well as the PD were able to condense pDNA completely as no migration of pDNA down any Lane A was observed (Fig. 8). Significant differences between the protection from DNAse I afforded by the various lipids was, however, observed when the complexes were treated with DNAse I (Fig. 8, Lanes B). Generally, LPDs formulated using the Group 1 TC lipids, DOTMA or DOTAP protected pDNA to a greater extent than LPDs formulated with the DC lipids and, to a lesser extent, the Group 2 lipids. In the case of the DC lipids, a greater amount of low molar weight pDNA fragments were particularly evident. These differences were particularly noticeable when double strength DNAse I was used (Fig. 8b). Taken together these results suggest that the pDNA is well complexed by the peptide and that the lipids add little benefit in this respect. The different lipids however protect the pDNA to various extents supporting the hypothesis that the lipid forms a barrier between the lipid and DNA. This suggestion is further supported by the fact that no uncomplexed DNA was observed.

All complexes were able to release pDNA on the addition of excess pAsp (Lanes C), although the extent of release obtained varied with the nature of the lipid present. LPDs prepared using Group 1 TC lipids appeared to release more pDNA due to the presence of brighter pDNA bands and a lower residual fluorescence remaining in the wells. pDNA release from the LPDs prepared using the Group 2 TC lipids was intermediate with relatively low levels of fluorescence remaining in the wells. In an attempt to quantify the protection and release of pDNA from the complexes formed using the Group 1 lipids as well as those formed using DOTMA or DOTAP, the intensities of the DNA bands in Lane B were analysed and compared either to the amount of pDNA in their corresponding Lane C (*i.e.* the pDNA release in the absence of DNAse I) or the corresponding (untreated) pDNA control band using the data obtained in Fig. 8b (first sample on every row). The results of this intensity analysis are shown in Table 3. With the exception of TC-DOesDEG4, the complexes prepared with the Group 1 TC lipids, DOTMA and DOTAP exhibited much higher DNA band intensity (52% and higher) compared to their DC equivalents (28% and lower), suggesting that more DNA was preserved. This was also mirrored by observing brighter low molecular weight degradation bands for LPDs containing DC lipids compared to Group 1 TC lipids, DOTMA or DOTAP, providing further evidence that the DNA was digested to lower molecular weight fragments to a greater extent. These observations suggest that the bilayer formed by the TC lipids provides a more effective barrier to the degradation of DNA.

**Table 3:** Density analysis of gel electrophoresis data from Fig. 8b ^*a*^

Group 1 lipid present in the LPD	Intensity of lane B DNA band (as % of corresponding lane C band)	Intensity of lane B DNA band as % of untreated DNA control (the first sample in each row)
DODEG4	11	7
TC-DODEG4	81	95
DOSEG3	20	18
TC-DOSEG3	57	46
DOesDEG4	28	22
TC-DOesDEG4	5	4
Me-DOesDEG3	4	4
DOesSEG3	7	5
TC-DOesSEG3	52	52
Me-DOesSEG3	20	19
DOTMA	81	86
DOTAP	76	81
PD	35	41

^*a*^Data analysed using GelAnalyzer.

Since transfection efficiency was highest in LPDs prepared using DOTMA, DOTAP or the Group 1 and Group 2 TC lipids as opposed to their DC counterparts, and a similar trend was observed in the pDNA protection capacity, it is possible that the ability of the LPD complex to protect the pDNA and release it from the LPD particle plays a major role in enhancing its transfection efficiency. A similar relationship between transfection and the extent of protection and subsequent release of the DNA payload a complex carried has been observed in our previous studies^15^ as well as those reported by others.^28^–^30^

## Conclusions

The identification of a novel TC lipid, formed from the helper lipid DOPE and a cationic lipid possessing a terminal hydroxyl group, led us to design and synthesise several trichain lipids and the corresponding DC cytofectins. The transfection efficacies (in Opti-MEM and Opti-MEM/water) of the TC cytofectins in LPD ternary vectors was significantly enhanced compared to the DC analogues and therefore biophysical studies were performed to enhance understanding of the behaviour of lipids with three alkyl chains attached. In general, the size of the TC lipid vesicles and LPD nanoparticles were slightly larger than the corresponding DC-containing systems. TEM revealed that the TC lipids formed spherical vesicles, whereas for LPDs both spherical and rod-shaped particles were formed, with monodisperse particles for the DCs and more polydispersity with the TCs. SANS studies indicated that for vesicles with DC and TC lipids, multilamellar structures were present: when DC lipids were used this was low, rising to 25–50% for TC lipids. For LPD formulations, however, there was no evidence for the formation of multilamellar structures, only unilamellar structures of very large layer thickness, suggesting a re-arrangement of the lipids in the presence of DNA to accommodate the interaction between the TCs and the negatively charged DNA. This is the first time a re-arrangement of the lipid bilayer, on formation of lipopolyplexes, has been reported. CD spectra of the LPDs indicated that the peptide interacted with DNA preferentially to the lipid, which suggests that the structure of the LPD consists of a DNA-peptide core surrounded by a lipid bilayer as previously shown for other DC lipids.^15^ Finally gel electrophoresis studies revealed that the TC lipids in LPD formulations had enhanced pDNA protection properties, compared to the corresponding DC analogues. Thus, the major enhancement in transfection performance of the TC lipids is probably due to their ability to protect and release DNA. At a molecular level this may be achieved by folding back of the third hydrophobic chain into the bilayer or partial inter-digitation of the third alkyl chain into the other bilayer component. In summary, a new design concept for use in effective gene delivery lipids has been identified. Further studies are now underway to probe this effect.

## Experimental

Detailed experimental procedures, characterisation data, and ESI Fig. S1–S6 are in the ESI.†

## Conflicts of interest

There are no conflicts to declare.

## Supplementary Material

Supplementary information
