# Supplementary material for: The discovery and enhanced properties of trichain lipids in lipopolyplex gene delivery systems
Source: Org Biomol Chem. 2018 Dec 19;17(4):945–57. doi: 10.1039/c8ob02374c (PMC6350505; doi:10.1039/c8ob02374c)
Supplement: Supplementary file 1 [file OB-017-C8OB02374C-s001.pdf]

Electronic Supplementary Material (ESI) for Organic & Biomolecular Chemistry.
This journal is © The Royal Society of Chemistry 2019

The Discovery and Enhanced Properties of Trichain Lipids in Lipopolyplex Gene Delivery Systems

Atefeh Mohammadi, Laila Kudsiova, M. Firouz MohD Mustapa, Frederick Campbell, Danielle Vlaho, Katharina Welser, Harriet Story, Aristides D. Tagalakis, Stephen L. Hart, David J. Barlow, Alethea B. Tabor, M. Jayne Lawrence and Helen C. Hailes

SUPPORTING INFORMATION

General materials and methods S3

Synthetic procedures S4

1-(2-Bromoethoxy)-2-(2-methoxyethoxy)ethane S4

***N*-(2,3-Bis((9*Z*)-octadecenyloxy)propyl)-*N*,*N*-dimethyl-13-oxo-3,6,9,12-tetraoxatriacont-(21*Z*)-en-1-aminium bromide TC-DODEG4** S4

***N*-(2-(2-(2-(2-Hydroxyethoxy)ethoxy)ethoxy)ethyl)-*N*,*N*-dimethyl-2,3-bis((9*Z*)-oleoyloxy)propan-1-aminium bromide DOesDEG4** S5

***N*,*N*-dimethyl-*N*-(3-(oleoyloxy)-2-((9*Z*)-2-oxooctadecenyloxy)propyl)-13-oxo-3,6,9,12-tetraoxatriacont-(21*Z*)-en-1-aminium bromide TC-DOesDEG4** S5

2-(2-(2-(2-Bromoethoxy)ethoxy)ethoxy)ethyl oleate 7 S6

***N*-(2-(2-(2-Methoxyethoxy)ethoxy)ethyl)-*N*,*N*-dimethyl-2,3-bis((oleoyloxy)propan-1-aminium bromide Me-DOesDEG3** S6

***N*-(2,3-Bis((oleoyloxy)propyl)-5-(2-(2-(2-hydroxyethoxy)ethoxy)ethoxy)-*N*,*N*-dimethyl-5-oxopentan-1-aminium bromide DOesSEG3** S7

***N*-(2,3-Bis((oleoyloxy)propyl)-*N*,*N*-dimethyl-12-oxo-2,5,8,11-tetraoxahexadecan-16-aminium bromide Me-DOesSEG3** S7

(9*Z*)-2-(2-(2-(5-Bromopentanoyloxy)ethoxy)ethoxy)ethyl oleate S8

***N*-(2,3-bis((9*Z*)-octadecenyloxy)propyl)-*N*,*N*-dimethyl-5,16-dioxo-6,9,12,15-tetraoxatritriacont-(24*Z*)-en-1-aminium bromide TC-DOSEG3** S8

***N*,*N*-dimethyl-*N*-(3-((9*Z*)-octadecenyloxy)-2-(oleoyloxy)-3-oxopropyl)-5,16-dioxo-6,9,12,15-tetraoxatritriacont-(24*Z*)-en-1-aminium bromide TC-DOesSEG** S9

2-(2-(2-(Bromoethoxy)ethoxy)ethoxy)ethyl acetate 10 S10

2-(2-(2-(2-Bromoethoxy)ethoxy)ethoxy)ethyl octanoate 11 S10

2-(2-(2-(2-Bromoethoxy)ethoxy)ethoxy)ethyl dodecanoate 12 S11

***N*-(2,3-Bis((9*Z*)-octadecenyloxy)propyl)-*N*,*N*-dimethyl-13-oxo-3,6,9,12-tetraoxatetradecan-1-aminium bromide AC-DODEG4** S11

***N*-(2,3-Bis((oleoyloxy)propyl)-*N*,*N*-dimethyl-13-oxo-3,6,9,12-tetraoxatetradecan-1-aminium bromide AC-DOesDEG4** S12

***N*-(2,3-Bis((9*Z*)-octadecenyloxy)propyl)-*N*,*N*-dimethyl-13-oxo-3,6,9,12-tetraoxaicosan-1-aminium bromide OC-DODEG4** S12

***N*-(2,3-Bis((9*Z*)-octadecenyloxy)propyl)-*N*,*N*-dimethyl-13-oxo-3,6,9,12-tetraoxatetracosan-1-aminium bromide DO-DODEG4** S13

_N_-(2,3-Bis(oleoyloxy)propyl)-_N_,_N_-dimethyl-13-oxo-3,6,9,12-tetraoxatetracosan-1-aminium bromide DO-DOesDEG4 S13

Scrambled peptide A synthesis (sc) S14

HPLC Methods S15

^{1}H and ^{13}C NMR spectra of compounds S17

HPLC and MS of Scrambled peptide A (sc) S32

Formulation, transfection and biophysical studies S33

Lipid transesterification studies S33

Transfection procedures and biophysical studies S33

Preparation of vesicles S33

Formulation of lipopolyplexes S33

Dynamic light scattering S34

Cell culture S34

***In vitro* transfection experiments** S34

Gel retardation, release and protection assay S35

Transmission electron microscopy S36

Small angle neutron scattering S36

Circular dichroism S37

Figures S38

Figure S1 S38

Figure S2 S39

Figure S3 S40

Figure S4 S41

Figure S5 S42

Figure S6 S43

References S43

General materials and methods

Synthesis Unless otherwise noted, solvents and reagents for synthesis were reagent grade from commercial suppliers and used without further purification. Dry CH_2Cl_2 and MeCN were obtained using anhydrous alumina columns.[1] All moisture-sensitive reactions were performed under a nitrogen or argon atmosphere using oven-dried glassware. Reactions were monitored by TLC on Kieselgel 60 F_{254} plates with detection by UV, potassium permanganate, and phosphomolybdic acid stains. Flash column chromatography was carried out using silica gel (particle size 40-63 µm). ^{1}H NMR and ^{13}C NMR spectra were recorded on a Bruker AMX300 MHz, AMX400, Avance-500 MHz and Avance-600 MHz machines. Chemical shifts (in ppm) are quoted relative to tetramethylsilane and referenced to residual pronated solvent. Coupling constants (*J*) are measured in Hertz (Hz) and multiplicities for ^{1}H NMR coupling are shown as s (singlet), d (doublet), t (triplet), q (quartet), quint (quintet) and m (multiplet). Mass spectrometry data was acquired at the UCL Chemistry Mass Spectrometry Facility on Thermo Finnegan MAT 900XP, Micromass Quattro LC electrospray and VG70-SE mass spectrometers. Also at the EPSRC National Mass Spectrometry Service Centre, Swansea University. Infrared spectra were recorded on a Shimadzu FTIR-8700 spectrometer. The tertiary amines 2,3-di-((9*Z*)-octadecenyloxy)propyl-*N*,*N*-dimethylamine (**4**) and (9*Z*)-3-(dimethylamino)propane-1,2-diyl dioleate (**6**).[2-4] DODEG4 was synthesised as previously described[5] as was DOSEG3[6]. 11-Bromo-3,6,9-trioxoundecan-1-ol (**5**), 2-(2-(2-hydroxyethoxy)ethoxy)ethyl 5-bromopentanoate (**8**), and 2-(2-(2-methoxyethoxy)ethoxy)ethyl 5-bromopentanoate (**9**) were prepared as previously described.[5,6] Peptide **A** and H-$(Lys)_{16}$-OH (**K16**) were synthesized as previously reported.[6]

Transfection and biophysical studies The lipids DOTMA (*N*-[1-(2,3-dioleyloxy)-propyl]-*N*,*N*,*N*-trimethylammonium chloride) and DOPE (dioleoylphosphatidylethanolamine) were purchased from TCI Europe N. V. Belgium, and Avanti Polar lipids, USA, respectively. Lipofectamine, Lipofectamine 2000 (L2K) and OptiMEM were purchased from Invitrogen Life Technologies, UK. The gWIZ luciferase plasmid (pDNA) was supplied from Aldevron, USA. Calf thymus DNA (ctDNA) and all other cell culture media were supplied from Sigma, Poole, UK. All reagents for transfection and biophysical studies were used without further purification and were of the highest grade possible. De-ionised double distilled water was used throughout. D_2O (> 99.9% D) was purchased from Aldrich, Poole, UK.

Synthetic procedures

1-(2-Bromoethoxy)-2-(2-methoxyethoxy)ethane.

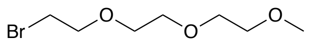

To a solution of triethylene glycol monomethyl ether (3.0 mL, 19 mmol) in dichloromethane (50 mL) at 0 °C, thionyl bromide (2.0 mL, 26 mmol mmol) was added dropwise. The reaction was stirred at room temperature for 18 h, the organic phase washed with water (2 × 50 mL), dried ($MgSO_4$) and concentrated *in vacuo*. The crude product was purified by flash silica chromatography (hexane/Et_2O, 4:1) to give 1-(2-bromoethoxy)-2-(2-methoxyethoxy)ethane[7] as a colourless oil (2.0 g, 47%). R_F 0.26 (hexane/Et_2O, 4:1); ν_{max}(neat)/cm^{-1} 2850, 1458; ^{1}H NMR (500 MHz; $CDCl_3$) δ 3.36 (3H, s, OCH_3), 3.37 (2H, m, OCH_2), 3.45 (2H, t, *J* = 6.4 Hz, CH_2Br), 3.47 (6H, m, 3 x OCH_2), 3.80 (2H, t, *J* = 6.4 Hz, OCH_2); *m/z* [HRMS ES+] found MH$^+$ 227.0283. $C_7H_{16}O_3{}^{79}Br$ requires 227.0283.

***N*-(2,3-Bis((9*Z*)-octadecenyloxy)propyl)-*N*,*N*-dimethyl-13-oxo-3,6,9,12-tetraoxatriacont-(21*Z*)-en-1-aminium bromide TC-DODEG4.**

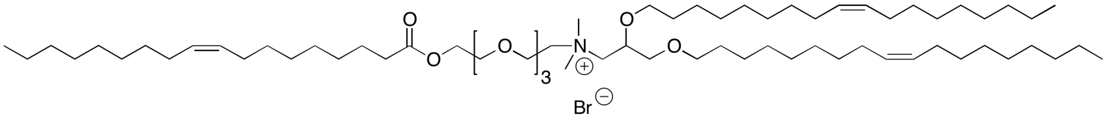

A solution of the amine **4**[2] (0.260 g, 0.420 mmol) and the 4-EG oleoyl ester **7** (0.240 g, 0.460 mmol) in acetone (2 mL) were stirred in a sealed tube at 90 °C for 48 h. The acetone was removed *in vacuo*. Purification by flash silica chromatography (CH_2Cl_2/MeOH, 19:1) yielded **TC-DODEG4** as a pale yellow oil (171 mg, 36%). R_F 0.40 (CH_2Cl_2/MeOH, 9:1); ν_{max}(neat)/cm^{-1} 2950, 2859, 1740, 1464; ^{1}H NMR (600 MHz; $CDCl_3$) δ 0.83 (9H, t, *J* = 7.0 Hz, 3 x CH_3), 1.13–1.22 (64H, m, 32 x CH_2), 1.51 (4H, m, 2 x OCH$_2$CH_2), 1.57 (2H, m, CH_2CH$_2$CO$_2$), 1.92–1.97 (12H, m, 6 x CH_2CH=CH), 2.29 (2H, t, *J* = 7.7 Hz, CH_2CO$_2$), 3.38–3.51 (14H, m, 7 x CH_2), 3.51 (6H, m, 2 x CH_3N), 3.58 (4H, m, 2 x CH_2), 3.92–4.05 (4H, m, 2 x CH_2), 4.08 (1H, m, *H*CO), 4.18 (2H, m, CH_2OCO), 5.27 (6H, m, 3 x C*H*=C*H*); ^{13}C NMR (150 MHz; $CDCl_3$) δ 14.2, 22.8, 25.0, 26.1, 26.3, 27.26, 27.30, 29.0, 29.1–29.9 (signals superimposed), 30.1, 32.1, 32.7, 34.2, 53.3, 53.4, 63.3, 65.0, 65.2, 66.8, 68.6, 69.29, 69.32, 70.3, 70.4, 70.5, 70.6, 72.1, 73.5, 127.9–130.5 (signals superimposed), 173.9 (C=O); *m/z* [HRMS ES+] found [M-Br]$^+$ 1060.9904. $C_{67}H_{130}NO_7$ requires 1060.9847; *m/z* (+ES) 1061 ([M-Br]$^+$, 100%), 980 (60), 931 (70), 843 (65), 306 (63).

***N*-(2-(2-(2-(2-Hydroxyethoxy)ethoxy)ethoxy)ethyl)-*N*,*N*-dimethyl-2,3-bis(oleoyloxy)propan-1-aminium bromide DOesDEG4.**

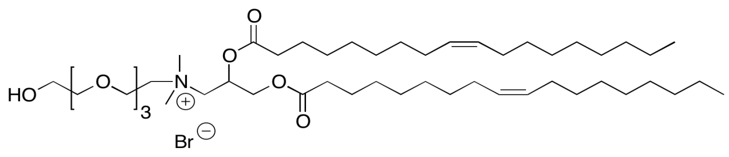

A solution of amine **6**[3] (0.590 g, 0.910 mmol) and the 4-EG bromide **5**[5] (0.280 g, 1.09 mmol) in THF (2 mL) were stirred in a sealed tube at 90 °C for 48 h. The THF was evaporated *in vacuo* then the crude product purified by flash silica chromatography (CH_2Cl_2/MeOH, 19:1) to give **DOesDEG4** as a pale yellow oil (232 mg, 28%). R_F 0.21 (CH_2Cl_2/MeOH, 9:1); ν_{max}(neat)/cm^{-1} 3400, 2924, 1741, 1465; ^{1}H NMR (400 MHz; $CDCl_3$) δ 0.86 (6H, t, *J* = 6.4 Hz, 2 x C*H*$_3$), 1.25–1.28 (40H, m, 2 x C*H*$_2$), 1.58 (4H, m, 2 x C*H*$_2$CH$_2$CO$_2$), 2.00 (8H, m, 4 x C*H*$_2$CH=CH), 2.28–2.34 (4H, m, 2 x C*H*$_2$CO$_2$), 3.42 (3H, s, C*H*$_3$N), 3.51 (3H, s, C*H*$_3$N), 3.61–4.15 (18H, m, C*H*$_2$N, 6 × C*H*$_2$O, C*H*$_2$OH, OCHCH*H*, CH*H*OCO), 4.27 (1H, d, *J* = 14.0 Hz, C*H*HOCO), 4.48 (1H, dd, *J* = 13.5 and 3.2 Hz, OCHC*H*H), 5.33 (4H, m, 2 x C*H*=C*H*), 5.66 (1H, m, C*H*OCO); ^{13}C NMR (100 MHz; $CDCl_3$) δ 14.1, 22.7, 24.65 and 24.71, 27.16 and 27.20, 29.1–29.7 (signals superimposed), 31.9, 33.9, 34.2, 53.0, 54.4, 61.0, 63.3, 63.7, 64.8, 65.1, 65.9, 69.8, 70.0, 70.3, 72.5, 129.61 and 129.64, 129.99 and 130.01, 172.9 (C=O), 173.2 (C=O); *m/z* [HRMS ES+] found [M-Br]$^+$ 824.6939. $C_{49}H_{94}NO_8$ requires 824.6979; *m/z* (+ES) 825 ([M-Br]$^+$, 50%), 722 (20), 561 (100).

***N*,*N*-Dimethyl-*N*-(3-(oleoyloxy)-2-((9*Z*)-2-oxooctadecenyloxy)propyl)-13-oxo-3,6,9,12-tetraoxatriacont-(21*Z*)-en-1-aminium bromide TC-DOesDEG4.**

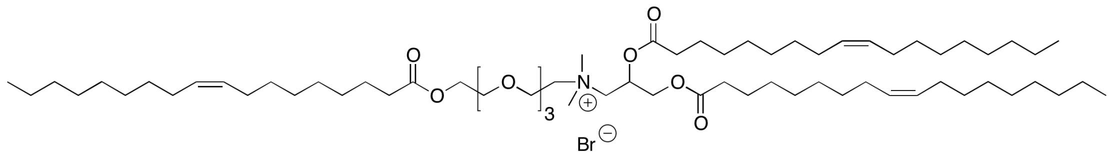

A solution of the amine **6**[3] (0.610 g, 0.941 mmol) and the 4-EG oleoyl ester **7** (0.640 g, 1.22 mmol) in acetone (2 mL) were stirred in a sealed tube at 80 °C for 48 h. The acetone was removed *in vacuo*. Purification by flash silica chromatography (CH_2Cl_2/MeOH, 19:1) yielded **TC-DOesDEG4** as a pale yellow oil (118 mg, 11%). R_F 0.24 (CH_2Cl_2/MeOH, 19:1); ν_{max} (neat)/cm^{-1} 2924, 2854, 1740, 1465; ^{1}H NMR (500 MHz; $CDCl_3$) δ 0.86 (9H, t, *J* = 6.8 Hz, 3 x C*H*$_3$), 1.25–1.32 (60H, m, 30 x C*H*$_2$), 1.58 (6H, m, 3 x C*H*$_2$CH$_2$CO$_2$), 1.99 (12H, m, 6 x C*H*$_2$CH=CH), 2.30 (4H, t, *J* = 7.5 Hz, 2 x C*H*$_2$CO$_2$), 2.34 (2H, m, C*H*$_2$CO$_2$), 3.44 (3H, s, C*H*$_3$N), 3.51 (3H, s, C*H*$_3$N), 3.64–3.68 (10H, m, 5 x C*H*$_2$), 3.82 (1H, dd, *J* = 13.5 and 10.0 Hz, CH*H*OCO), 3.94 (2H, m, C*H*$_2$), 4.05 (2H, m, 2 x C*H*), 4.13 (1H, m, C*H*), 4.20 (2H, t, *J* = 5.0 Hz, C*H*$_2$OCO), 4.35 (1H, d, *J* = 13.5 Hz, C*H*HOCO), 4.48 (1H, dd, *J* = 12.3 and 3.3 Hz, OCHC*H*H), 5.28–5.35 (6H, m, 3 x C*H*=C*H*), 5.64 (1H, m, C*H*OCO); ^{13}C NMR (125 MHz; $CDCl_3$) δ 14.2, 22.7, 24.7, 24.8, 25.0, 25.7, 27.2, 27.3, 29.1–29.8, 31.6, 32.0, 34.0, 34.3, 52.8, 52.9, 63.2, 63.3, 64.0, 64.9, 65.6, 65.8,

69.3, 70.1, 70.45, 70.48, 70.6, 129.7–130.1 (signals superimposed), 172.9 (C=O), 173.2 (C=O), 173.8 (C=O); *m/z* [HRMS ES+] found $[M-Br]^+$ 1088.9382. $C_{67}H_{126}NO_9$ requires 1088.9433; *m/z* (+ES) 1089 ($[M-Br]^+$, 100%).

(9*Z*)-2-(2-(2-(2-Bromoethoxy)ethoxy)ethoxy)ethyl oleate 7

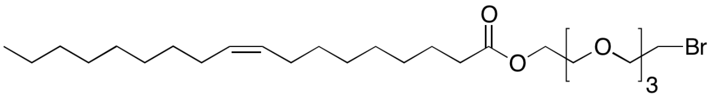

A solution of oleic acid (2.00 mL, 6.30 mmol), the 4-EG bromide **5**[4] (1.50 g, 5.83 mmol), and DMAP (70 mg, 0.58 mmol) in anhydrous dichloromethane (50 mL) were stirred at rt for 5 min. After cooling to 0 °C, *N*,*N*′-diisopropylcarbodiimide (DIC) (1.20 mL, 7.75 mmol) was added dropwise and the mixture stirred at rt for 18 h. The dichloromethane was removed *in vacuo*, ethyl acetate (60 mL) was added, and the mixture washed with sodium hydrogencarbonate (2 × 60 mL), brine (60 mL), dried (Na_2SO_4) and concentrated *in vacuo*. Purification by silica flash chromatography (EtOAc/hexane, 1:4) yielded **6** as a yellow oil (1.65 g, 54%). R_F 0.19 (EtOAc/hexane, 1:4); ν_{max}(neat)/cm^{-1} 2923, 1736, 1457; ^{1}H NMR (300 MHz; $CDCl_3$) δ 0.84 (3H, t, *J* = 6.7 Hz, CH_3), 1.18–1.30 (20H, m, 10 x CH_2), 1.58 (2H, m, CH_2CH_2CO_2), 1.93–2.00 (4H, m, 2 x CH_2CH=CH), 2.29 (2H, t, *J* = 7.6 Hz, CH_2CO_2), 3.43 (2H, t, *J* = 6.3 Hz, CH_2Br), 3.59–3.67 (10H, m, 5 x OCH_2), 3.77 (2H, t, *J* = 6.3 Hz, OCH_2CH_2Br), 4.19 (2H, t, *J* = 4.8 Hz, CH_2OCO), 5.27–5.36 (2H, m, C*H*=C*H*); ^{13}C NMR (75 MHz; $CDCl_3$) δ 14.1, 22.6, 24.9, 27.2, 29.1–29.8 (signals superimposed), 30.4, 31.9, 34.0, 63.3, 69.2, 70-4–70.6 (signals superimposed), 71.2, 129.7, 130.0, 173.6 (C=O); *m/z* [HRMS ES+] found $[MNa]^+$ 543.2671. $C_{26}H_{49}O_5{}^{79}BrNa$ requires 543.2661.

***N*-(2-(2-(2-Methoxyethoxy)ethoxy)ethyl)-*N*,*N*-dimethyl-2,3-bis((oleoyloxy)propan-1-aminium bromide Me-DOesDEG3.**

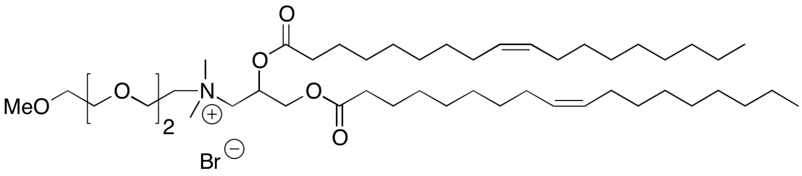

A solution of amine **6**[3] (210 mg, 0.324 mmol) and 1-(2-bromoethoxy)-2-(2-methoxyethoxy)ethane[7] (84 mg, 0.370 mmol) in acetone (2 mL) were stirred in a sealed tube at 90 °C for 48 h. The acetone was evaporated *in vacuo* and product purified by flash silica chromatography (CH_2Cl_2/MeOH, 19:1) to give **Me-DOesDEG3** as a pale yellow oil (158 mg, 56%). ν_{max} (neat)/cm^{-1} 2923, 2853, 1741, 1465; ^{1}H NMR (500 MHz; $CDCl_3$) δ 0.87 (6H, t, *J* 6.8 Hz, 2 x CH_3), 1.23–1.28 (40H, m, 10 x CH_2), 1.57 (4H, m, 2 x CH_2CH_2CO_2), 1.97 (8H, m, 4 x CH_2CH=CH), 2.28 (4H, m, 2 x CH_2CO_2), 3.32 (3H, s, OCH_3), 3.44 (3H, s, NCH_3), 3.47 (2H, m, CH_2), 3.50 (3H, s, NCH_3), 3.57 (4H, m, 2 x CH_2), 3.65 (2H, m, CH_2), 3.80 (1H, dd, *J* = 14.0 and 8.0 Hz, CH*H*OCO), 3.92 (2H, m, CH_2), 4.02 (2H, m, 2 x C*H*), 4.07 (1H, m, C*H*), 4.30 (1H, d, *J* = 14.0 Hz, C*H*HOCO), 4.46 (1H, dd, *J* = 13.0 and 3.0 Hz, OCHC*H*H), 5.26–5.31 (4H, m, 2 x C*H*=C*H*),

5.61 (1H, m, C*H*OCO); ^{13}C NMR (125 MHz; $CDCl_3$) δ 14.1, 22.7, 24.7 and 24.8, 27.2 and 27.3, 29.1–29.8 (signals superimposed), 31.9, 33.9, 34.2, 52.7, 52.9, 59.0, 63.3, 64.1, 64.9, 65.4, 65.9, 70.0, 70.3, 71.9, 129.7, 130.1, 172.9 (C=O), 173.1 (C=O); *m/z* [HRMS FAB+] found $[M-Br]^+$ 794.6886. $C_{48}H_{92}NO_7$ requires 794.6873; *m/z* (+FAB) 794 ($[M-Br]^+$, 100%), 299 (13), 190 (16).

***N*-(2,3-Bis(oleoyloxy)propyl)-5-(2-(2-(2-hydroxyethoxy)ethoxy)ethoxy)-*N*,*N*-dimethyl-5-oxopentan-1-aminium bromide DOesSEG3.**

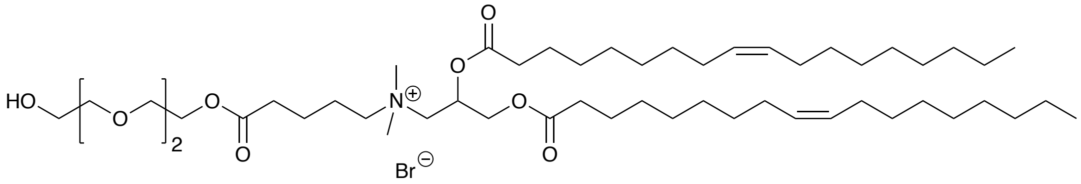

A solution of the amine **6**[3] (0.500 g, 0.772 mmol) and bromide **8**[6] (0.310 g, 0.990 mmol) in acetone (2 mL) were stirred in a sealed tube at 80 °C for 48 h. Acetone was evaporated *in vacuo* and the product purified by flash silica chromatography (CH_2Cl_2/MeOH, 19:1) to give **DOesSEG3** as a pale yellow oil (579 mg, 78%). R_F 0.24 (CH_2Cl_2/MeOH, 9:1); ν_{max}(neat)/cm^{-1} 3408, 2924, 1736, 1650, 1264; ^{1}H NMR (600 MHz; $CDCl_3$) δ 0.78 (6H, t, *J* = 7.1 Hz, 2 x CH_3), 1.17–1.24 (40H, m, 20 x CH_2), 1.49 (4H, quint, *J* = 7.0 Hz, C$H_2$$CH_2CO_2$), 1.61 (quint, *J* = 7.2 Hz, C$H_2$$CH_2CO_2$), 1.82 (2H, m, C$H_2$$CH_2$N), 1.89–1.96 (8H, m, C$H_2$CH=CH), 2.19–2.27 (4H, m, 2 x C$H_2$$CO_2$), 2.39 (2H, t, *J* = 7.2 Hz, C$H_2$$CO_2$), 3.30 (3H, s, NC$H_3$), 3.32 (3H, s, NC$H_3$), 3.41–3.65 (13H, m, 6 × CH_2, O*H*), 3.71 (1H, dd, *J* = 14.0 and 8.9 Hz, CH*H*OCO), 4.04 (dd, *J* = 12.1 and 5.9 Hz, OCHCH*H*), 4.15 (2H, m, CH_2OCO), 4.27 (1H, d, *J* = 14.0 Hz, C*H*HOCO), 4.44 (1H, dd, *J* = 12.1 and 3.4 Hz, OCHC*H*H), 5.23 (4H, m, 2 x C*H*=*CH*), 5.53 (1H, m, C*H*OCO); ^{13}C NMR (100 MHz; $CDCl_3$) δ 14.6, 21.4, 22.1, 22.8, 24.4, 24.5, 27.4, 29.0–29.9 (signals superimposed), 32.1, 33.1, 34.1, 34.4, 51.8, 53.5, 61.7, 63.7, 64.1, 65.3, 65.7, 69.1, 70.3 (signals superimposed), 70.7, 72.7, 129.7, 130.2, 172.7 (C=O), 172.9 (C=O), 173.3 (C=O); *m/z* (+ES) 881 ($[M-Br]^+$, 100%), 663 (15).

***N*-(2,3-Bis(oleoyloxy)propyl)-*N*,*N*-dimethyl-12-oxo-2,5,8,11-tetraoxahexadecan-16-aminium bromide Me-DOesSEG3.**

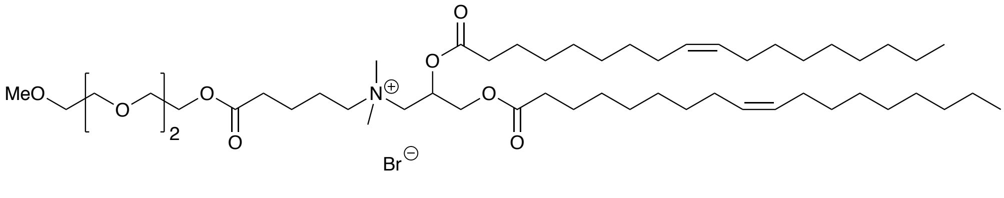

A solution of the amine **6**[3] (0.210 g, 0.324 mmol) and the bromide **9**[6] (0.210 g, 0.642 mmol) in acetone (2 mL) were stirred in a sealed tube at 90 °C for 48 h. Acetone was evaporated *in vacuo* and the product purified by silica flash chromatography (CH_2Cl_2/MeOH, 19:1) to give **Me-DOesSEG3** as a

pale yellow oil (249 mg, 78%). R_F 0.32 (CH_2Cl_2/MeOH, 9:1); ν_{max}(neat)/cm^{-1} 2923, 2854, 1741, 1465; ^{1}H NMR (600 MHz; $CDCl_3$) δ 0.79 (6H, t, J = 6.3 Hz, 2 x CH_3), 1.18–1.25 (40H, m, 20 x CH_2), 1.47 (4H, m, C$H_2$$CH_2CO_2$), 1.60 (2H, quint, J = 7.5 Hz, CH_2), 1.77 (2H, m, CH_2), 1.92–1.98 (8H, m, 4 x CH_2CH=CH), 2.24 (2H, t, J = 7.4 Hz, C$H_2$$CO_2$), 2.27 (2H, t, J = 7.6 Hz, C$H_2$$CO_2$), 2.38 (2H, m, C$H_2$$CO_2$), 3.28 (3H, s, OC$H_3$), 3.33 (3H, s, NC$H_3$), 3.36 (3H, s, NC$H_3$), 3.46 (2H, m, C$H_2$), 3.50–3.69 (10H, m, 5 x CH_2), 3.72 (1H, dd, J = 14.0 and 8.6 Hz, CH*H*OCO), 4.04 (1H, dd, J = 12.2 and 6.0 Hz, OCHCH*H*), 4.13 (2H, m, CH_2OCO), 4.32 (1H, d, J = 14.0 Hz, C*H*HOCO), 4.44 (1H, dd, J = 12.2 and 3.5 Hz, OCHC*H*H), 5.21–5.28 (4H, m, 2 x C*H*=C*H*), 5.53 (1H, m, C*H*OCO); ^{13}C NMR (150 MHz; $CDCl_3$) δ 14.1, 21.4, 22.1, 22.7, 24.7, 24.8, 27.2, 29.2–29.8 (signals superimposed), 31.9, 33.1, 33.9, 34.2, 51.7, 51.9, 59.0, 63.3, 63.6, 65.0, 65.6, 69.0, 70.4–70.5 (signals superimposed), 71.9, 128.0–130.8 (signals superimposed), 172.70 (C=O), 172.74 (C=O), 173.1 (C=O); *m/z* [HRMS FAB+] found [M-Br]$^+$ 894.7378. $C_{53}H_{100}NO_9$ requires 894.7393; *m/z* (FAB+) 895 ([M-Br]$^+$, 100%), 307 (65).

(9*Z*)-2-(2-(2-(5-Bromopentanoyloxy)ethoxy)ethoxy)ethyl oleate.

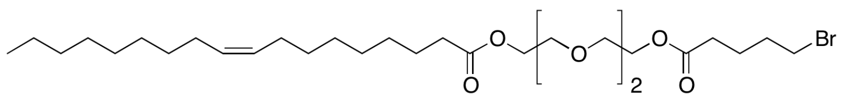

The reaction was carried out under anhydrous conditions. A solution of oleic acid (2.00 mL, 6.42 mmol), bromide **8**[6] (1.80 g, 5.84 mmol) and DMAP (70 mg, 0.58 mmol) in dichloromethane (50 mL) were stirred at rt for 5 min. After cooling to 0 °C, DIC (1.18 mL, 7.59 mmol) was added dropwise and the mixture stirred at rt for 18 h. The dichloromethane was removed *in vacuo* and ethyl acetate (60 mL) added, then the mixture washed with sodium carbonate (2 × 60 mL) and brine (60 mL), dried (Na_2SO_4) and concentrated *in vacuo*. Purification by flash silica chromatography (EtOAc/hexane, 2:8) yielded the titled compound as a yellow oil (2.50 g, 75%). R_F 0.42 (hexane/EtOAc, 7:3); ν_{max}(neat)/cm^{-1} 2923, 2854, 1737, 1705, 1520, 1457; ^{1}H NMR (600 MHz; $CDCl_3$) δ 0.89 (3H, t, J = 6.9 Hz, CH_3), 1.15–1.30 (20H, m, 10 x CH_2), 1.62 (2H, m, C$H_2$$CH_2CO_2$), 1.78 (2H, quint, J = 7.5 Hz, C$H_2$$CH_2$Br), 1.89 (2H, quint, J = 6.7 Hz, C$H_2$$CH_2CO_2$), 2.00 (4H, m, 2 x C$H_2$CH=CH), 2.31 (2H, t, J = 7.5 Hz, C$H_2$$CO_2$), 2.38 (2H, t, J = 7.3 Hz, C$H_2$$CO_2$), 3.41 (2H, t, J = 6.7 Hz, CH_2Br), 3.65 (4H, m, 2 x OCH_2), 3.69 (4H, m, 2 x OCH_2), 4.23 (4H, m, 2 x CH_2OCO), 5.33 (2H, m, C*H*=C*H*); ^{13}C NMR (150 MHz; $CDCl_3$) δ 14.1, 22.8, 23.5, 25.0, 27.2, 27.3, 29.2, 29.3, 29.5 (signals superimposed), 29.7, 29.8, 29.9, 32.0, 32.1, 33.2, 33.3, 34.3, 63.4, 63.6, 69.3, 69.4, 70.7, 129.9, 130.1, 173.2 (C=O), 174.0 (C=O); *m/z* [HRMS ES+] found [MNa]$^+$ 599.2892. $C_{29}H_{53}{}^{79}BrNaO_6$ requires 599.2923; *m/z* (ES+) 601 ([MNa]$^+$(^{81}Br), 95%), 599 ([MNa]$^+$(^{79}Br), 100), 431 (82), 324 (68).

***N*-(2,3-bis((9*Z*)-octadecenyloxy)propyl)-*N*,*N*-dimethyl-5,16-dioxo-6,9,12,15-tetraoxatritriacont-(24*Z*)-en-1-aminium bromide TC-DOSEG3.**

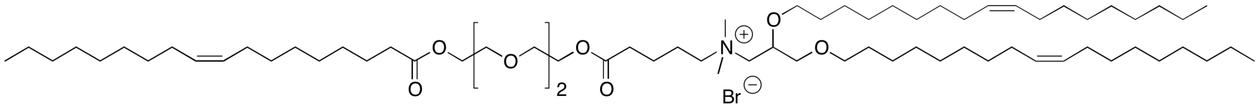

A solution of the amine **4**[2] (0.610 g, 0.984 mmol) and 2-(2-(2-(5-bromopentanoyloxy)ethoxy)ethoxy)ethyl oleate (0.640 g, 1.11 mmol) in THF (2 mL) were stirred in a sealed tube at 90 °C for 48 h. The THF was evaporated *in vacuo*. Purification by flash silica chromatography (CH_2Cl_2/MeOH, 19:1) yielded **TC-DOSEG3** as a pale yellow oil (129 mg, 11%). R_F 0.15 (CH_2Cl_2/MeOH, 99:1); ν_{max}(neat)/cm^{-1} 2924, 2853, 1737, 1465; ^{1}H NMR (600 MHz; $CDCl_3$) δ 0.86 (9H, t, *J* = 7.0 Hz, 3 x C*H*$_3$), 1.23–1.30 (64H, m, 32 x C*H*$_2$), 1.53 (4H, m, 2 x OCH_2C*H*$_2$), 1.58 (2H, m, C*H*$_2$$CH_2CO_2$), 1.68 (2H, quint, *J* = 7.2 Hz, C*H*$_2$$CH_2CO_2$), 1.78–1.88 (2H, m, C*H*$_2$), 1.93–2.03 (12H, m, 6 x C*H*$_2$CH=CH), 2.30 (2H, t, *J* = 7.6 Hz, C*H*$_2$$CO_2$), 2.43 (2H, t, *J* = 7.2 Hz, C*H*$_2$$CO_2$), 3.36 (3H, s, NC*H*$_3$), 3.39 (3H, s, NC*H*$_3$), 3.40–3.42 (4H, m, 2 x C*H*$_2$), 3.52 (1H, dd, *J* = 10.6 and 2.9 Hz, CH*H*OCO), 3.57 (1H, dd, *J* = 10.6 and 4.6 Hz, C*H*HOCO), 3.63–3.72 (11H, m, 5 x C*H*$_2$ and C*H*), 3.89 (1H, d, *J* = 13.5 Hz, OCHC*H*H), 4.04 (1H, m, *H*CO), 4.21 (4H, m, 2 x C*H*$_2$OCO), 5.33 (6H, m, 3 x C*H*=C*H*); ^{13}C NMR (150 MHz; $CDCl_3$) δ 14.3, 21.6, 22.2, 22.8, 25.0, 26.1, 26.3, 27.3, 29.2–29.9 (signals superimposed), 30.1, 32.0, 32.7, 33.2, 34.3, 52.2, 63.4, 63.8, 65.3, 65.9, 68.4, 69.2, 69.3, 69.4, 70.6, 72.1, 73.4, 129.8–130.6 (signals superimposed), 172.8 (C=O), 173.9 (C=O); *m/z* [HRMS FAB+] found [M-Br]$^+$ 1117.0055. $C_{70}H_{134}NO_8$ requires 1117.0109; *m/z* (FAB+) 1117 ([M-Br]$^+$, 100%), 663 (15).

***N*,*N*-dimethyl-*N*-(3-((9*Z*)-octadecenyloxy)-2-(oleoyloxy)-3-oxopropyl)-5,16-dioxo-6,9,12,15-tetraoxatritriacont-(24*Z*)-en-1-aminium bromide TC-DOesSEG3.**

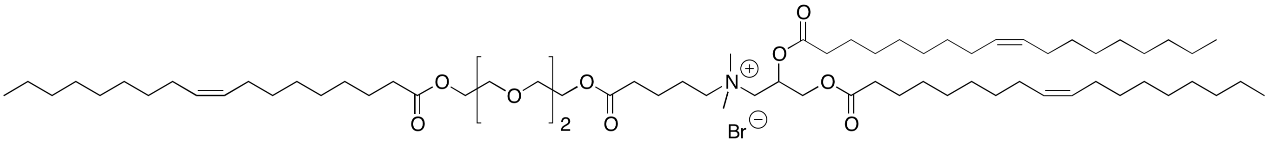

A solution of the amine **6**[3] (0.710 g, 1.10 mmol) and 2-(2-(2-(5-bromopentanoyloxy)ethoxy)ethoxy)ethyl oleate (0.680 g, 1.18 mmol) in acetone (2 mL) were stirred in a sealed tube at 80 °C for 48 h. The acetone was evaporated *in vacuo* and the product purified by flash silica chromatography (CH_2Cl_2/MeOH, 19:1) to give **TC-DOesSEG3** as a pale yellow oil (600 mg, 45%). R_F 0.21 (CH_2Cl_2/MeOH, 19:1); ν_{max}(neat)/cm^{-1} 2923, 2853, 1740, 1465; ^{1}H NMR (500 MHz; $CDCl_3$) δ 0.83 (9H, t, *J* = 7.1 Hz, 3 x C*H*$_3$), 1.20–1.29 (60H, m, 30 x C*H*$_2$), 1.55 (6H, m, 3 x C*H*$_2$$CH_2CO_2$), 1.65 (2H, quint, *J* = 7.4 Hz, C*H*$_2$), 1.82 (2H, m, C*H*$_2$), 1.98 (12H, m, 6 x C*H*$_2$CH=CH), 2.25–2.31 (6H, m, 3 x C*H*$_2$$CO_2$), 2.41 (2H, t, *J* = 7.4 Hz, C*H*$_2$$CO_2$), 3.38 (3H, s, NC*H*$_3$), 3.41 (3H, s, NC*H*$_3$), 3.54–3.65 (10H, m, 5 x C*H*$_2$), 3.77 (1H, dd, *J* = 14.0 and 8.5 Hz, CH*H*OCO), 4.06 (1H, dd, *J* = 12.0 and 6.0 Hz, OCHCH*H*), 4.17 (4H, m, C*H*$_2$OCO), 4.38 (1H, d, *J* = 14.0 Hz, C*H*HOCO), 4.48 (1H, dd, *J* = 12.0 and 3.5 Hz, OCHC*H*H), 5.25–5.32 (6H, m, 3 x C*H*=C*H*), 5.56 (1H, m, C*H*OCO); ^{13}C NMR (125 MHz; $CDCl_3$) δ 14.1, 21.5, 22.1,

22.6, 22.7, 24.7, 24.8, 24.9, 25.7, 27.2, 29.1–29.8 (signals superimposed), 31.5, 31.9, 32.6, 33.0, 33.9, 34.2, 51.7, 52.0, 63.3, 63.5, 63.7, 65.0, 65.6, 69.1–69.2 (signals superimposed), 70.5, 129.6–130.2 (signals superimposed), 172.7 (C=O), 172.8 (C=O), 173.1 (C=O), 173.8 (C=O); *m/z* [HRMS ES+] found $[M-Br]^+$ 1144.9756. $C_{70}H_{130}NO_{10}$ requires 1144.9695; *m/z* (+ES) 1145 ($[M-Br]^+$, 100%).

2-(2-(2-(Bromoethoxy)ethoxy)ethoxy)ethyl acetate 10.

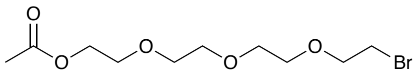

To a solution of **5** (1.00 g, 3.89 mmol) and 4-dimethylaminopyridine (0.095 g, 0.78 mmol) in pyridine (10 mL) acetic anhydride (0.80 g, 7.8 mmol) was added dropwise. The reaction was then heated at 60 °C for 4 h and the pyridine was removed *in vacuo*. Dichloromethane (10 mL) was added, and the solution was washed with HCl (1 M; 100 mL), saturated $NaHCO_3$ (100 mL), and brine (100 mL), dried ($MgSO_4$) and concentrated *in vacuo*. Purification *via* flash silica column chromatography (EtOAc/hexane, 3:2) yielded **10** as a yellow oil (0.412 g, 35%). R_F 0.48 (EtOAc/hexane, 4:1); ν_{max}(neat)/cm^{-1} 2868, 1735; ^{1}H NMR (600 MHz; $CDCl_3$) δ 2.08 (3H, s, C*H*$_3$CO$_2$), 3.47 (2H, t, *J* = 6.3 Hz, C*H*$_2$Br), 3.67–3.69 (8H, m, 4 x C*H*$_2$O), 3.70 (2H, t, *J* = 4.8 Hz, C*H*$_2$CH$_2$OCO), 3.81 (2H, t, *J* = 6.3 Hz, C*H*$_2$CH$_2$Br), 4.22 (2H, t, *J* = 4.8 Hz, C*H*$_2$OCO); ^{13}C NMR (150 MHz, $CDCl_3$) δ 21.0, 30.3, 63.6, 69.2, 70.5, 70.60, 70.64, 70.7, 71.2, 171.1 (C=O); *m/z* [HRMS FAB+] found $[MH]^+$ 299.0503. $C_{10}H_{20}{}^{79}BrO_5$ requires 299.0494; *m/z* (ES+) 323 ($[MNa(^{81}Br)]^+$, 98%), 321 ($[MNa(^{79}Br)]^+$, 100).

2-(2-(2-(2-Bromoethoxy)ethoxy)ethoxy)ethyl octanoate 11.

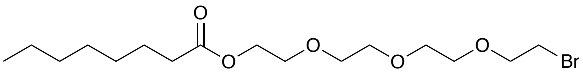

Thionyl chloride (1.19 g, 10.0 mmol) was added dropwise to octanoic acid (0.72 g, 5.0 mmol) and the reaction was heated at reflux for 4 h. Excess thionyl chloride was removed *in vacuo* to yield the acid chloride which was directly added dropwise to a solution of **5** (0.643 g, 2.50 mmol), 4-dimethylaminopyridine (61 mg, 0.50 mmol) and triethylamine (0.556 g, 5.49 mmol) in anhydrous dichloromethane (150 mL). The reaction mixture was stirred at room temperature for 18 h. The reaction was then washed with saturated $NaHCO_3$ (150 mL) and brine (150 mL), dried ($MgSO_4$), and concentrated *in vacuo* to give **11** as a pale yellow oil (1.01 g, quantitative yield) NMR spectroscopic analysis indicated that it was of high purity so it was used directly in the next step. R_F 0.31 (CH_2Cl_2/MeOH, 99:1); ν_{max}(neat)/cm^{-1} 2925, 2857, 1734; ^{1}H NMR (500 MHz; $CDCl_3$) δ 0.88 (3H, t, *J* = 7.0 Hz, C*H*$_3$), 1.27–1.30 (8H, m, 4 x C*H*$_2$), 1.62 (2H, quint, *J* = 7.5 Hz, C*H*$_2$CH$_2$CO$_2$), 2.33 (2H, t, *J* = 7.5 Hz, C*H*$_2$CO$_2$), 3.47 (2H, t, *J* = 6.3 Hz, C*H*$_2$Br), 3.66–3.71 (8H, m, 4 x OC*H*$_2$), 3.70 (2H, t, *J* = 4.9 Hz,

CH_2CH_2OCO), 3.81 (2H, t, *J* = 6.3 Hz, CH_2CH_2Br), 4.23 (2H, t, *J* = 4.9 Hz, CH_2OCO); ^{13}C NMR (150 MHz, $CDCl_3$) δ 14.1, 22.7, 25.0, 29.0, 29.2, 30.4, 31.7, 34.3, 63.4, 69.3, 70.6, 70.66, 70.72, 70.8, 71.3, 173.9 (C=O); *m/z* [HRMS FAB+] found $[MH]^+$ 383.1436. $C_{16}H_{32}{}^{79}BrO_5$ requires 383.1433; *m/z* (ES+) 407 ($[MNa(^{81}Br)]^+$, 98%), 405 ($[MNa(^{79}Br)]^+$, 100), 298 (38), 254 (50), 172 (100).

2-(2-(2-(2-Bromoethoxy)ethoxy)ethoxy)ethyl dodecanoate 12.

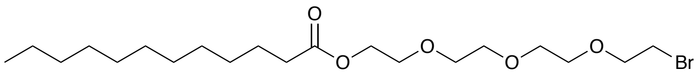

Thionyl chloride (0.452 g, 3.80 mmol) was added dropwise to dodecanoic acid (0.381 g, 1.90 mmol) and the reaction was heated at reflux for 4 h. Excess thionyl chloride was removed *in vacuo* to yield the acid chloride which was directly added dropwise to a solution of **5** (0.500 g, 1.94 mmol), 4-dimethylaminopyridine (46 mg, 0.38 mmol) and triethylamine (0.422 g, 4.17 mmol) in anhydrous dichloromethane (150 mL). The reaction mixture was stirred at room temperature for 18 h. The reaction was then washed with saturated $NaHCO_3$ (150 mL) and brine (150 mL), dried ($MgSO_4$), and concentrated *in vacuo*. Purification *via* flash silica column chromatography (CH_2Cl_2 to CH_2Cl_2/MeOH, 200:1) yielded **12** as a pale yellow oil (0.448 g, 53% yield). R_F 0.35 (CH_2Cl_2/MeOH, 99:1); ν_{max}(neat)/cm^{-1} 2923, 2854, 1735; ^1H NMR (500 MHz; $CDCl_3$) δ 0.88 (3H, t, *J* = 7.0 Hz, CH_3), 1.25–1.29 (16H, m, 8 x CH_2), 1.60-1.62 (2H, m, $CH_2CH_2CO_2$), 2.32 (2H, t, *J* = 7.6 Hz, CH_2CO_2), 3.47 (2H, t, *J* = 6.3 Hz, CH_2Br), 3.66–3.68 (8H, m, 4 x OCH_2), 3.70 (2H, t, *J* = 4.9 Hz, CH_2CH_2OCO), 3.81 (2H, t, *J* = 6.3 Hz, CH_2CH_2Br), 4.23 (2H, t, *J* = 3.9 Hz, CH_2OCO); ^{13}C NMR (150 MHz, $CDCl_3$) δ 14.2, 22.8, 25.0, 29.2, 29.3, 29.4, 29.5, 29.7, 30.4, 32.0, 34.3, 63.4, 69.3, 70.6, 70.66, 70.71, 70.8, 71.3, 173.9 (C=O); *m/z* [HRMS FAB+] found $[MH]^+$ 439.2052. $C_{20}H_{40}{}^{79}BrO_5$ requires 439.2059; *m/z* (ES+) 459 ($[MNH_4(^{81}Br)]^+$, 100%), 457 ($[MNH_4(^{79}Br)]^+$, 100), 227 (95).

***N*-(2,3-Bis((9*Z*)-octadecenyloxy)propyl)-*N*,*N*-dimethyl-13-oxo-3,6,9,12-tetraoxatetradecan-1-aminium bromide AC-DODEG4.**

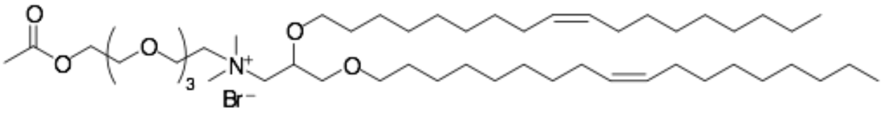

A solution of the amine **4**[2] (0.188 g, 0.303 mmol) and **10** (0.109 g, 0.364 mmol) in acetone (2 mL) were stirred in a sealed tube at 90 °C for 48 h. The acetone was removed *in vacuo*. Purification by flash silica chromatography (CH_2Cl_2/MeOH, 50:1 to 19:1) yielded **AC-DODEG4** as a yellow oil (0.079 g, 28%). R_F 0.35 (CH_2Cl_2/MeOH, 9:1); ν_{max}(neat)/cm^{-1} 2924, 2855, 1737, 1465; ^1H NMR (600 MHz; $CDCl_3$) δ 0.87 (6H, t, *J* = 7.1 Hz, 2 x CH_3), 1.25–1.28 (44H, m, 22 x CH_2), 1.54–1.60 (4H, m, 2 x

$OCH_2CH_2CH_2$), 1.96–2.00 (8H, m, 4 × CH_2CH=CH), 2.08 (3H, s, CH_3CO_2), 3.42–3.70 (24H, m, 9 x CH_2, 2 x NCH_3), 3.93–4.13 (5H, m, C*H*, 2 x CH_2), 4.22 (2H, t, *J* = 4.9 Hz, CH_2OCO), 5.32–5.38 (4H, m, 2 x C*H*=C*H*); ^{13}C NMR (150 MHz, $CDCl_3$) δ 14.3, 21.2, 22.8, 26.2, 26.4, 27.3, 29.5–29.9 (signals superimposed), 32.0, 32.7, 53.4, 53.5, 63.5, 65.0, 65.3, 67.0, 68.5, 69.3, 69.4, 70.3, 70.5–70.6 (signals superimposed), 72.1, 73.6, 129.8, 129.9, 130.11, 130.14, 171.1 (C=O); *m/z* [HRMS ES+] found $[M-Br]^+$ 838.7500. $C_{51}H_{100}NO_7$ requires 838.7494; *m/z* (+ES) 839 ($[M-Br]^+$, 100%), 813 (35).

N-(2,3-Bis(oleoyloxy)propyl)-N,N-dimethyl-13-oxo-3,6,9,12-tetraoxatetradecan-1-aminium bromide AC-DOesDEG4.

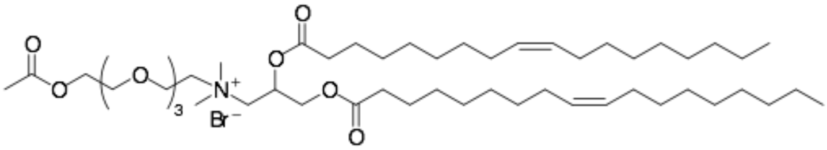

A solution of the amine **6**[3] (0.194 g, 0.300 mmol) and **10** (0.109 g, 0.364 mmol) in acetone (2 mL) were stirred in a sealed tube at 90 °C for 48 h. The acetone was removed *in vacuo*. Purification by flash silica chromatography (EtOAc then CH_2Cl_2/MeOH, 99:1 to 19:1) yielded **AC-DOesDEG4** as a yellow oil (0.155 g, 55%). R_F 0.32 (CH_2Cl_2/MeOH, 9:1); ν_{max}(neat)/cm^{-1} 2923, 2854, 1737, 1464; ^{1}H NMR (600 MHz; $CDCl_3$) δ 0.87 (6H, t, *J* = 7.1 Hz, 2 x CH_3), 1.24–1.35 (44H, m, 22 x CH_2), 1.57–1.60 (4H, m, 2 x CH_2CH_2CO), 1.98–2.04 (8H, m, 4 × CH_2CH=CH), 2.08 (3H, s, CH_3CO_2), 2.29–2.35 (4H, m, 2 x CH_2CO_2), 3.45 (3H, s, NCH_3), 3.52 (3H, s, NCH_3), 3.81 (1H, dd, *J* = 14.0 and 8.6 Hz, NCH*H*CHOCO), 3.94–3.98 (2H, m, CH_2), 4.05–4.22 (5H, m, OCHCH*H,* 2 x CH_2), 4.37 (1H, d, *J* = 14.0 Hz, NC*H*HCHOCO), 4.49 (1H, dd, *J* = 12.1 and 3.5 Hz, OCHC*H*H), 5.30–5.36 (4H, m, 3 x C*H*=C*H*), 5.65 (1H, m, C*H*OCO); ^{13}C NMR (150 MHz, $CDCl_3$) δ 14.2, 21.1, 22.8, 24.8, 24.9, 27.3, 29.2–29.8 (signals superimposed), 29.9, 32.0, 34.0, 34.3, 52.9, 53.0, 63.4, 63.5, 64.1, 65.0, 65.7, 65.9, 69.3, 70.1, 70.5–70.6 (signals superimposed), 129.7, 130.1, 171.1 (C=O), 172.9 (C=O), 173.3 (C=O); *m/z* [HRMS ES+] found $[M-Br]^+$ 866.7091. $C_{51}H_{96}NO_9$ requires 866.7080; *m/z* (+ES) 867 ($[M-Br]^+$, 100%).

N-(2,3-Bis((9Z)-octadecenyloxy)propyl)-N,N-dimethyl-13-oxo-3,6,9,12-tetraoxaicosan-1-aminium bromide OC-DODEG4.

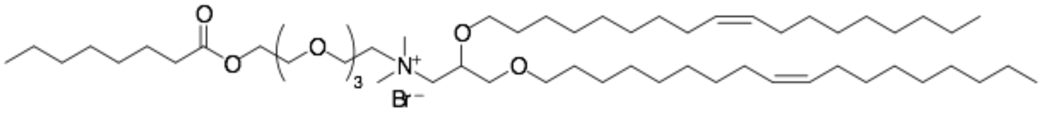

A solution of the amine **4**[2] (0.188 g, 0.303 mmol) and **11** (0.172 g, 0.449 mmol) in acetone (2 mL) were stirred in a sealed tube at 90 °C for 48 h. The acetone was removed *in vacuo*. Purification by flash silica chromatography (EtOAc then CH_2Cl_2/MeOH, 99:1 to 19:1) yielded **OC-DODEG4** as a yellow

oil (0.167 g, 55%). R_F 0.32 (CH_2Cl_2/MeOH, 9:1); ν_{max}(neat)/cm^{-1} 2923, 2854, 1737, 1464; ^{1}H NMR (600 MHz; $CDCl_3$) δ 0.86 (9H, t, *J* = 7.1 Hz, 3 x C*H*$_3$), 1.26–1.29 (52H, m, 26 x C*H*$_2$), 1.52–1.62 (6H, m, 2 x OCH_2C*H*$_2$$CH_2$ and C*H*$_2$$CH_2CO_2$), 1.92–2.03 (8H, m, 4 × C*H*$_2$CH=CH), 2.10 (2H, t, *J* = 7.6 Hz, C*H*$_2$$CO_2$), 3.43–3.69 (24H, m, 9 x C*H*$_2$, 2 x NC*H*$_3$), 3.96–4.12 (5H, m, C*H*, 2 x C*H*$_2$), 4.22 (2H, t, *J* = 5.0 Hz, C*H*$_2$OCO), 5.30–5.38 (4H, m, 2 x C*H*=C*H*); ^{13}C NMR (150 MHz, $CDCl_3$) δ 14.1, 14.2, 22.7, 22.8, 25.0, 26.1, 26.3, 27.3, 29.0, 29.2, 29.3, 29.4–29.9 (signals superimposed), 30.1, 31.7, 32.0, 32.7, 34.3, 53.5, 53.6, 63.2, 65.1, 65.4, 67.0, 68.6, 69.3, 69.4, 70.3, 70.5–70.6 (signals superimposed), 72.1, 73.6, 129.8, 130.1, 173.9 (C=O); *m/z* [HRMS ES+] found [M-Br]$^+$ 922.8441. $C_{57}H_{112}NO_7$ requires 922.8433; *m/z* (+ES) 923 ([M-Br]$^+$, 100%).

***N*-(2,3-Bis((9*Z*)-octadecenyloxy)propyl)-*N*,*N*-dimethyl-13-oxo-3,6,9,12-tetraoxatetracosan-1-aminium bromide DO-DODEG4.**

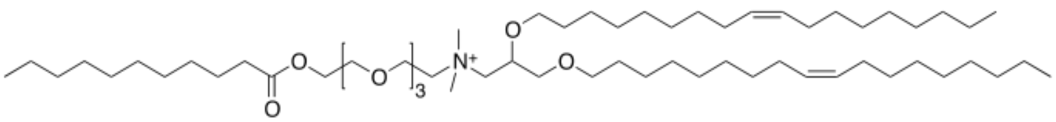

A solution of the amine **4**[2] (0.186 g, 0.300 mmol) and **12** (0.158 g, 0.360 mmol) in acetone (2 mL) were stirred in a sealed tube at 90 °C for 48 h. The acetone was removed *in vacuo*. Purification by flash silica chromatography (EtOAc then CH_2Cl_2/MeOH, 99:1 to 19:1) yielded **DO-DODEG4** as a yellow oil (0.060 g, 19%). R_F 0.31 (CH_2Cl_2/MeOH, 9:1); ν_{max}(neat)/cm^{-1} 2923, 2854, 1736, 1464; ^{1}H NMR (600 MHz; $CDCl_3$) δ 0.87 (9H, t, *J* = 6.9 Hz, 3 x C*H*$_3$), 1.20–1.31 (54H, m, 27 x C*H*$_2$), 1.50–1.80 (12H, m, 6 x C*H*$_2$), 1.95–2.05 (8H, m, 4 × C*H*$_2$CH=CH), 2.32 (2H, t, *J* = 7.6 Hz, C*H*$_2$$CO_2$), 3.42–3.68 (20H, m, 5 x C*H*$_2$ and 2 x NC*H*$_3$), 3.90–4.11 (5H, m, C*H* and 2 x C*H*$_2$), 4.22 (2H, t, *J* = 4.9 Hz, C*H*$_2$OCO), 5.32–5.35 (4H, m, 2 x C*H*=C*H*); ^{13}C NMR (150 MHz, $CDCl_3$) δ 14.1, 22.8, 25.0, 26.2, 26.4, 27.3, 29.3–29.7 (signals superimposed), 29.9, 30.1, 31.95, 32.03, 34.3, 53.5, 53.6, 63.3, 65.0, 65.3, 67.1, 68.5, 69.3, 69.4, 70.3–70.6 (signals superimposed), 72.1, 73.6, 129.8, 130.1, 173.9 (C=O); *m/z* [HRMS ES+] found [M-Br]$^+$ 978.9098. $C_{61}H_{120}NO_7$ requires 978.9059; *m/z* (+ES) 979 ([M-Br]$^+$, 100%), 649 (86).

***N*-(2,3-Bis(oleoyloxy)propyl)-*N*,*N*-dimethyl-13-oxo-3,6,9,12-tetraoxatetracosan-1-aminium bromide DO-DOesDEG4.**

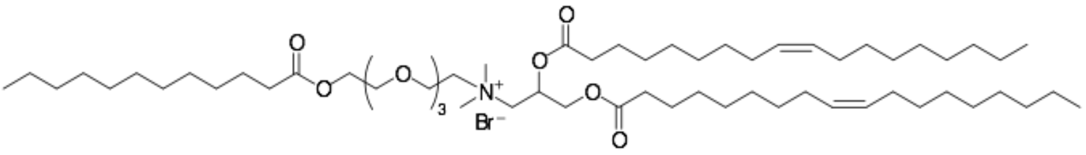

A solution of the amine **6**[3] (0.233 g, 0.360 mmol) and **12** (0.131 g, 0.298 mmol) in acetone (2 mL) were stirred in a sealed tube at 90 °C for 48 h. The acetone was removed *in vacuo*. Purification by

flash silica chromatography (EtOAc then CH_2Cl_2/MeOH, 99:1 to 19:1) yielded **DO-DOesDEG4** as a yellow oil (0.180 g, 56%). R_F 0.59 (CH_2Cl_2/MeOH, 9:1); ν_{max}(neat)/cm^{-1} 2919, 2850, 1736, 1629; ^{1}H NMR (600 MHz; $CDCl_3$) δ 0.87 (6H, t, *J* = 7.1 Hz, 2 x C*H*$_3$), 1.25–1.36 (56H, m, 28 x C*H*$_2$), 1.57–1.60 (6H, m, 3 x C*H*$_2$CH$_2$CO), 1.98–2.05 (8H, m, 4 × C*H*$_2$CH=CH), 2.30–2.35 (6H, m, 3 x C*H*$_2$CO$_2$), 3.44 (3H, s, NC*H*$_3$), 3.51 (3H, s, NC*H*$_3$), 3.58–3.70 (10H, m, 5 x C*H*$_2$), 3.80 (1H, dd, *J* = 14.2 and 8.5 Hz, NCH*H*CHOCO), 3.93–3.98 (2H, m, C*H*$_2$), 4.05–4.24 (5H, m, OCHCH*H,* 2 x C*H*$_2$), 4.37 (1H, d, *J* = 14.2 Hz, NC*H*HCHOCO), 4.49 (1H, dd, *J* = 12.2 and 3.4 Hz, OCHC*H*H), 5.31–5.37 (4H, m, 3 x C*H=CH*), 5.65 (1H, m, C*H*OCO); ^{13}C NMR (150 MHz, $CDCl_3$) δ 14.2, 22.8, 24.8, 24.9, 25.0, 27.3, 29.3–29.8 (signals superimposed), 29.9, 32.0, 34.0, 34.4, 52.9, 53.0, 63.2, 63.4, 64.1, 65.0, 65.8, 65.9, 69.3, 70.1, 70.5–70.6 (signals superimposed), 129.7, 130.2, 172.9 (C=O), 173.3 (C=O), 173.9 (C=O); *m/z* [HRMS ES+] found [M-Br]$^+$ 1006.8658. $C_{61}H_{116}NO_9$ requires 1006.8645; *m/z* (+ES) 1007 ([M-Br]$^+$, 100%).

Peptide A scrambled (K16-RVRRGA-CGRDCLG) **(sc): Peptide sc** was synthesized on a MultiSynTech Syro I automated system. Pre-loaded Fmoc-Gly-NovaSyn TGT resin (0.21 mmol/g, 150 mg, 31.5 µmol) was pre-swelled in DMF for 30 min prior to start of the synthesis. Standard Fmoc solid phase peptide synthesis (SPPS) was employed. The total volume of all reagents in each step was 1.5 mL. All reagents were dissolved in HPLC-grade DMF.

Fmoc Deprotection: To the reaction syringe containing Fmoc-Gly-NovaSyn TGT resin was added piperidine in DMF (40% v/v, 1.5 mL). The mixture was agitated for 20 s every minute for a total of 3 min. The reagents were removed by filtration under vacuum and the resin washed with DMF (4 x 1.5 mL). Piperidine in DMF solution (40% v/v, 0.75 mL) was added to the reaction syringe followed by DMF (0.75 mL) to make an overall 20% v/v solution of piperidine in DMF. This mixture was agitated for 20 s every minute for a total of 10 min. The reagents were removed by filtration under vacuum and the resin washed with DMF (6 x 1.5 mL).

Amino Acid Coupling: To the reaction syringe was added Fmoc-protected amino acid (0.60 mL, 0.56-0.84 M, 4-6 eq.), HBTU (0.60 mL, 0.56-0.84 M, 4-6 eq.) and DIPEA (0.30 mL, 1.12-1.68 M, 8-12 eq.). The mixture was agitated for 20 s every 3 min for a total of 40 min. The reagents were removed by filtration under vacuum and the resin washed with DMF (4 x 1.5 mL).

Formation of disulfide bonds via iodine oxidation: To the resin-bound, fully protected linear peptide was added I_2 (10 eq.) in DMF (3 mL). The reaction syringe was agitated for 2 h at room temperature. The iodine solution was drained from the syringe under vacuum and the resin washed with DMF (5 x 1.5 mL), 2% ascorbic acid in DMF (2 x 1.5 mL) and DMF (5 x 1.5 mL). The N-terminal Fmoc group was removed (as described) and the peptide cleaved from the resin.

Peptide Cleavage: TFA/TIPS/EDT/H_2O (94:2.5:2.5:1; 3 mL) was added to the reaction syringe. The syringe was then agitated for 3 h at room temperature. The cleavage cocktail was drained from the vessel under vacuum and diethyl ether (~10-15 mL) added to the filtrate. The resulting precipitate in solution was spun at 4000 rpm for 10 mins at 4 °C to produce a crude peptide pellet. The supernatant (diethyl ether) was decanted off and the peptide washed a further three times with diethyl ether. The crude peptide pellet was then re-dissolved in minimum water and freeze-dried for storage prior to purification.

Formation of disulfide bonds via aerial oxidation:

Crude peptides were re-dissolved in water (1 mg per 10 mL) and stirred at room temperature for 5 days. The solution was then concentrated and freeze-dried for storage prior to HPLC purification.

General peptide purification: The peptides were analyzed and purified *via* reverse phase HPLC using a Varian ProStar system with a Model 210 solvent delivery module and a Model 320 UV detector. The preparative purification was performed using a Discovery®BIO Wide Pore C18 (Varian; 100 x 21.2 mm, 5 µm beads, flow rate of 10 mL/min) loaded with 200-400 µL aliquots of a 10-20 mg/mL solution of peptide dissolved in 0.1% TFA containing water. The mobile phase was a decreasing gradient of water (0.1% TFA) in acetonitrile (0.1% TFA). The fractions containing the correct peak were pooled, the solvent removed under reduced pressure to approximately 2 mL and the solution freeze-dried.

The purified peptide was analyzed by analytical HPLC using an Onyx monolithic C18 column (Phenomenex®; 100 x 3.0 mm, 2 µm macropore size, 13 nm mesopore size, flow rate 0.85 mL/min). The analysis of the chromatograms was conducted using Star Chromatography Workstation software Version 1.9.3.2.

ESI-MS analysis was performed on a Waters Acquity Ultra Performance LC/MS machine.

HPLC Methods:

Preparative high performance liquid chromatography:

Discovery®BIO Wide Pore C18 (Varian; 100 x 21.2 mm, 5 µm beads) flow rate of 10 mL/min, and UV detection at 215 and 254 nm. Linear gradient: 10-50% B over 25 min, A = H_2O, 0.1% TFA, B = CH_3CN, 0.1% TFA).

Analytical high performance liquid chromatography:

Onyx monolithic C18 column (Phenomenex®; 100 x 3.0 mm, 2µm macropore size, 13nm mesopore size), flow rate 0.85 mL/min, UV detection at 215nm. Linear gradient: 5-90% B over 20 min, A = H_2O, 0.1% TFA, B = CH_3CN, 0.1% TFA). R_T 6.43 min.

^{1}H and ^{13}C NMR spectra of key compounds

TC-DODEG4

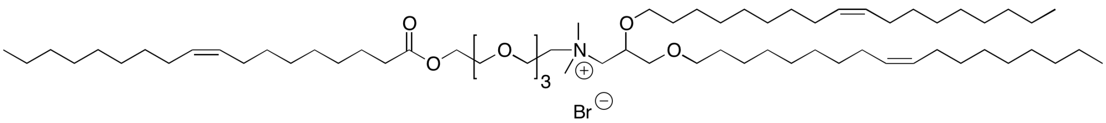

^{1}H NMR spectrum (600 MHz)

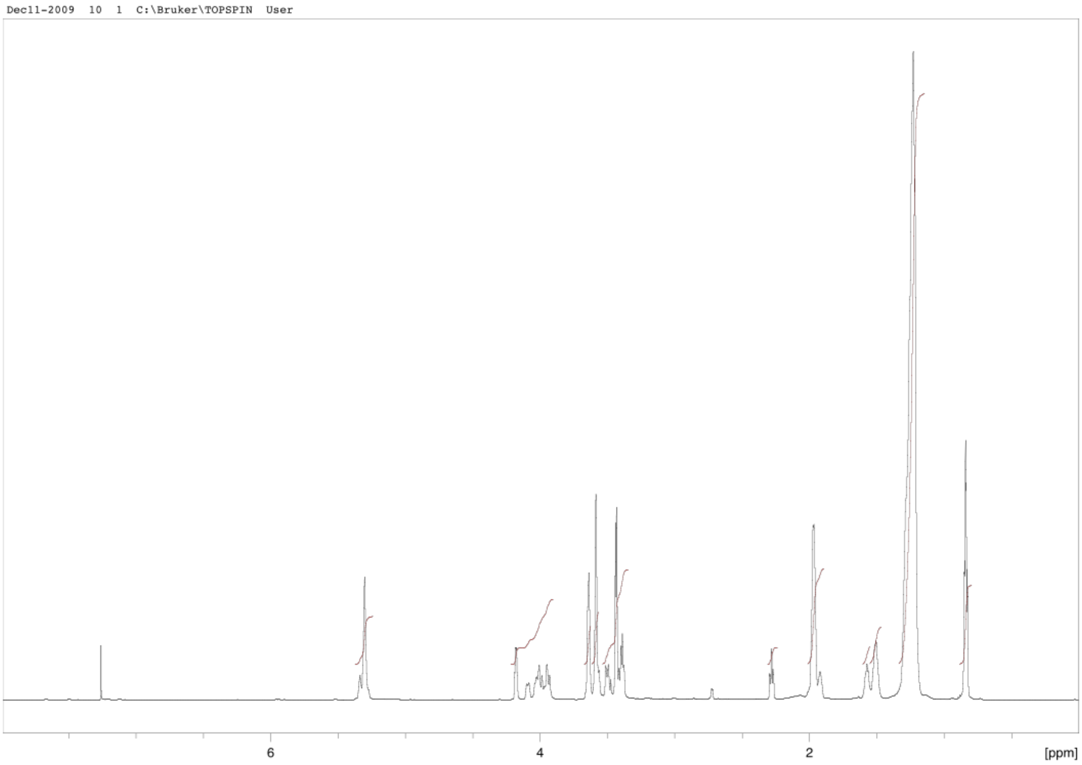

^{13}C NMR spectrum (150 MHz)

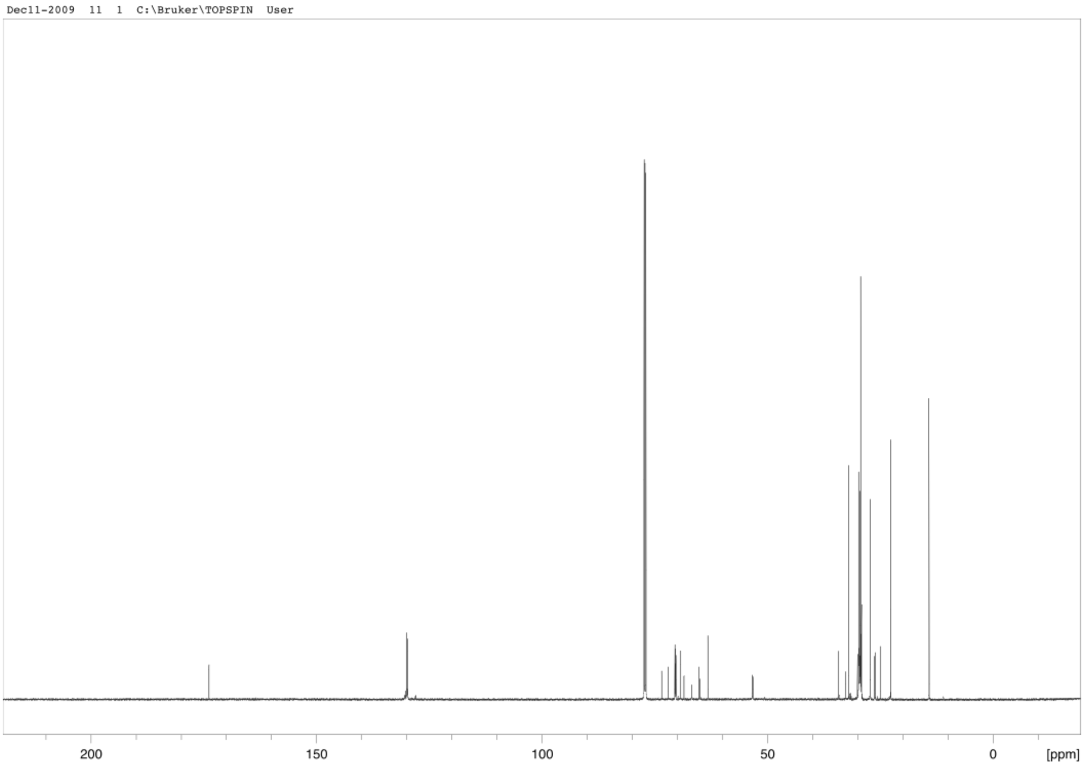

DOesDEG4

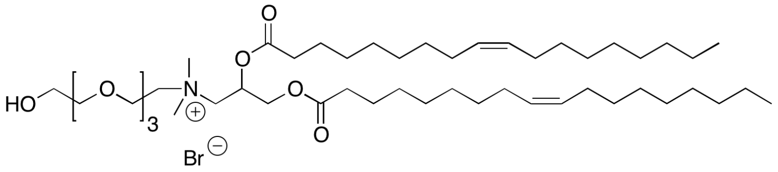

^1H NMR spectrum (400 MHz)

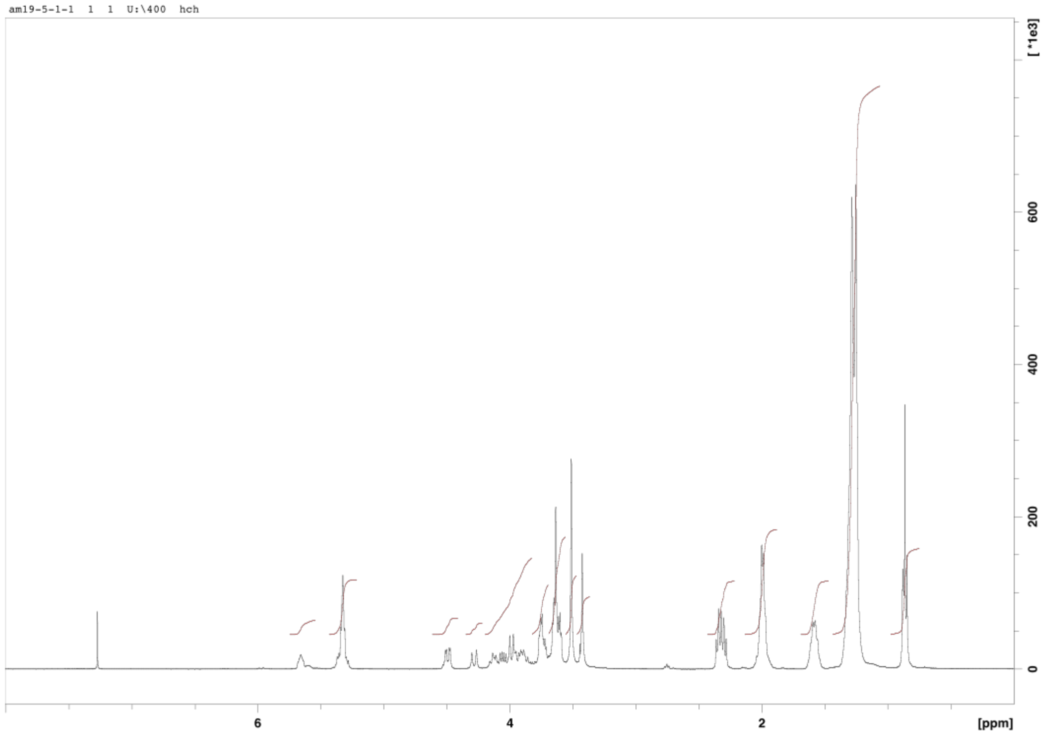

^{13}C NMR spectrum (100 MHz)

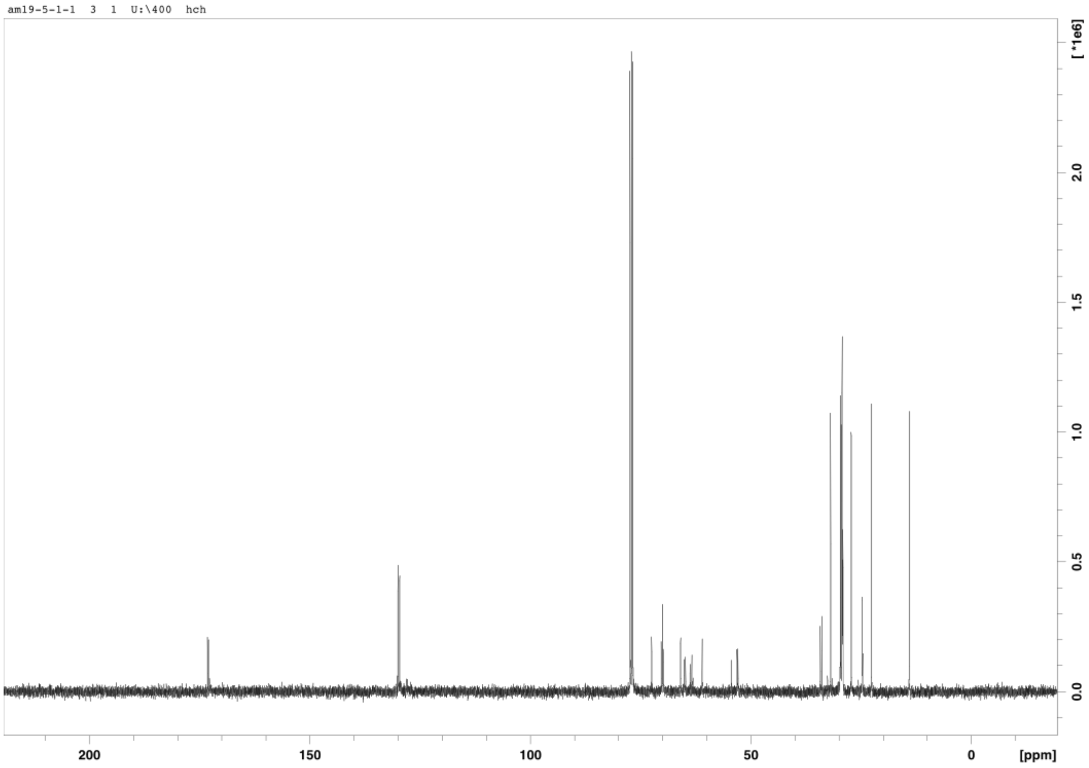

TC-DOesDEG4

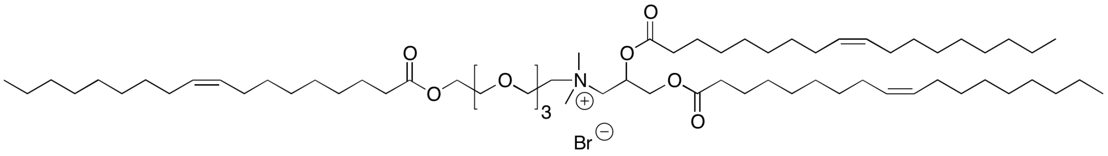

^{1}H NMR spectrum (500 MHz)

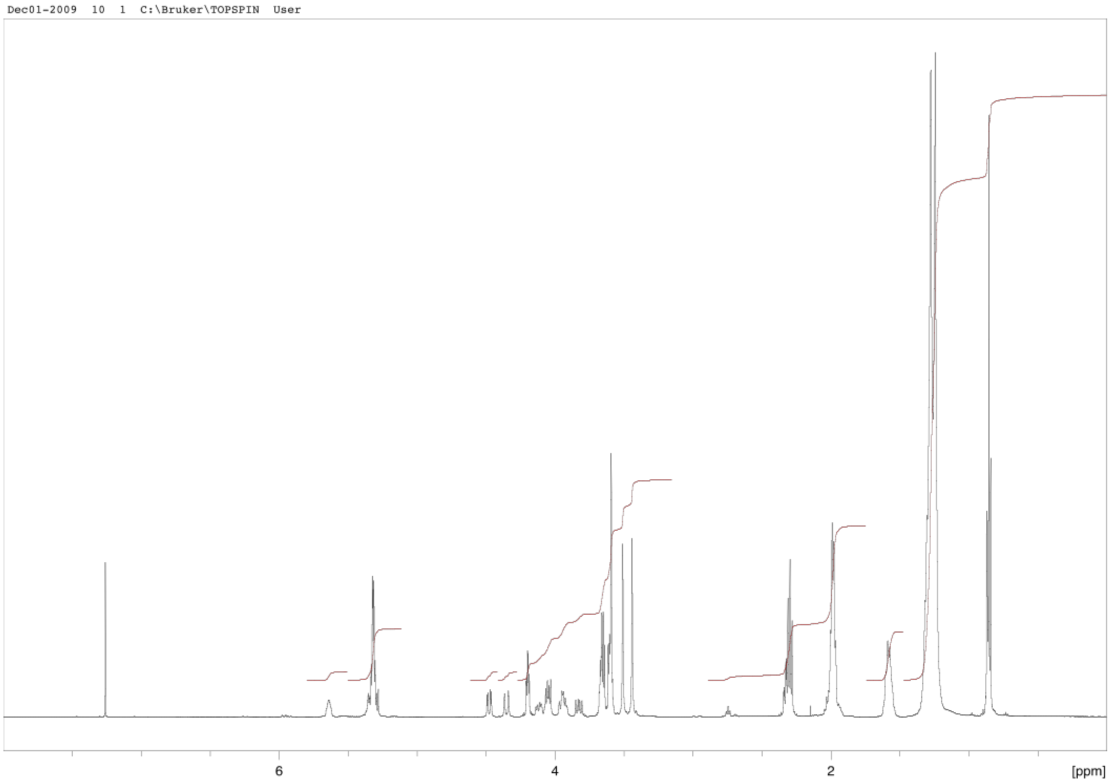

^{13}C NMR spectrum (125 MHz)

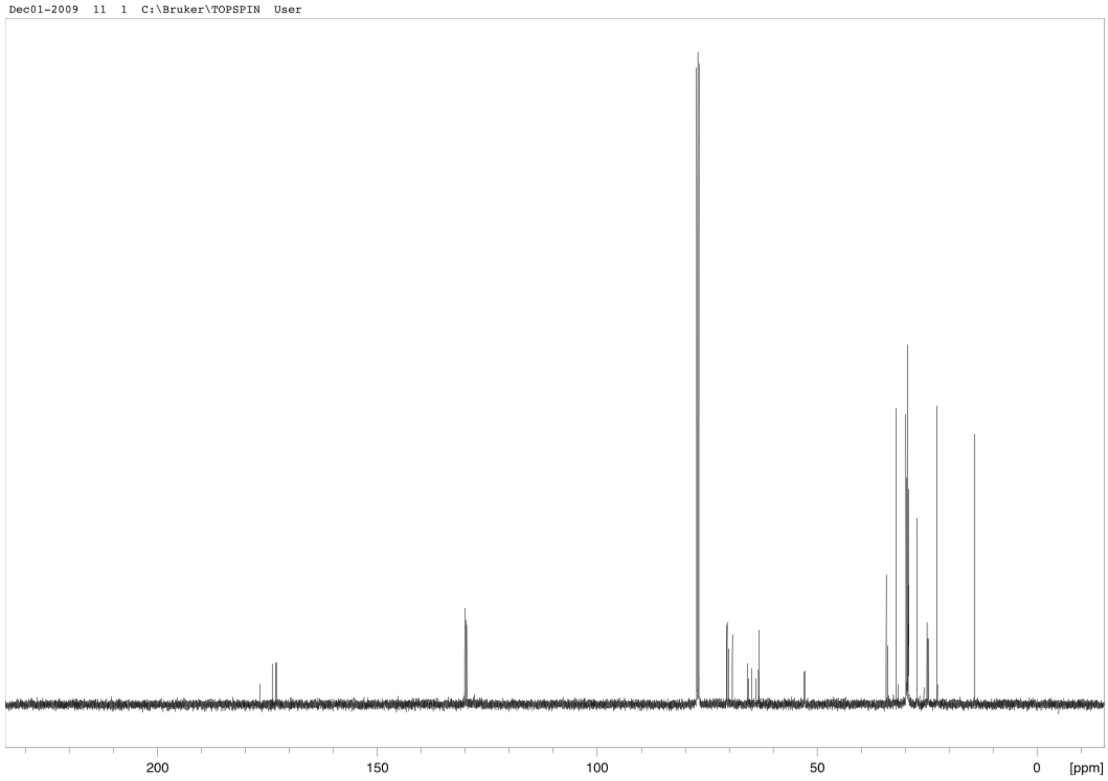

7

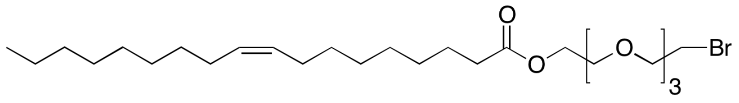

^{1}H NMR spectrum (300 MHz)

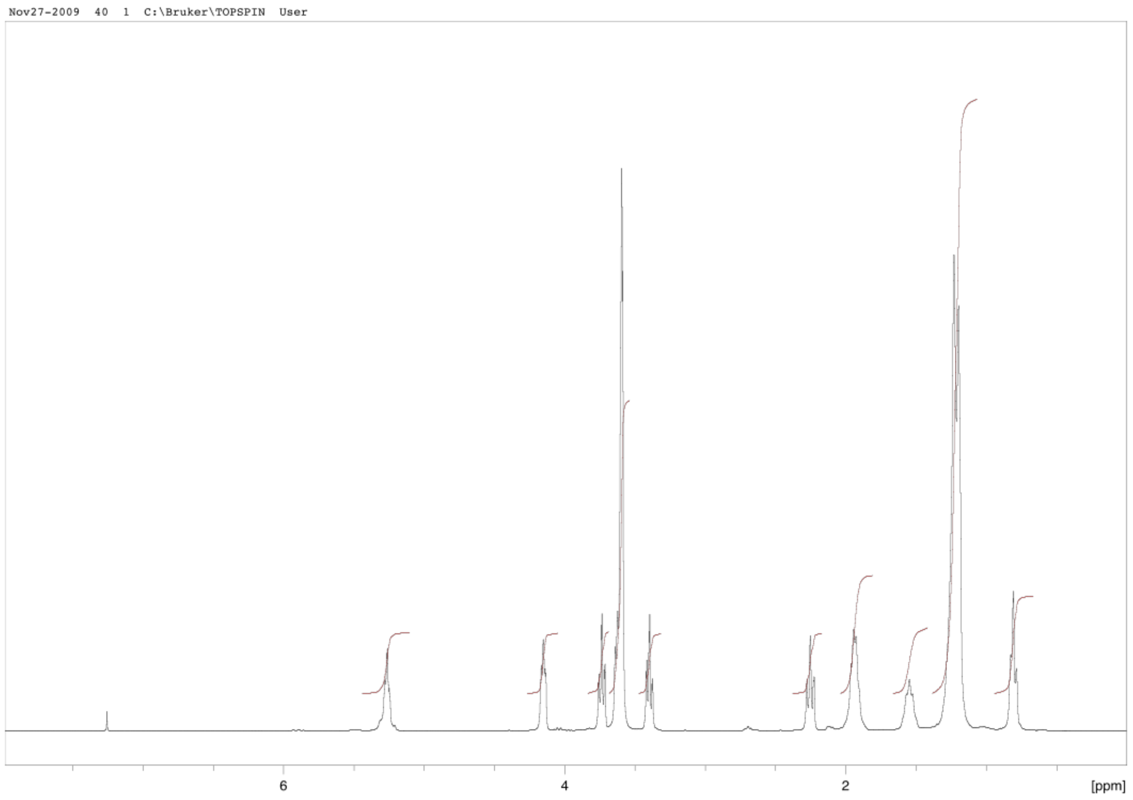

^{13}C NMR spectrum (75 MHz)

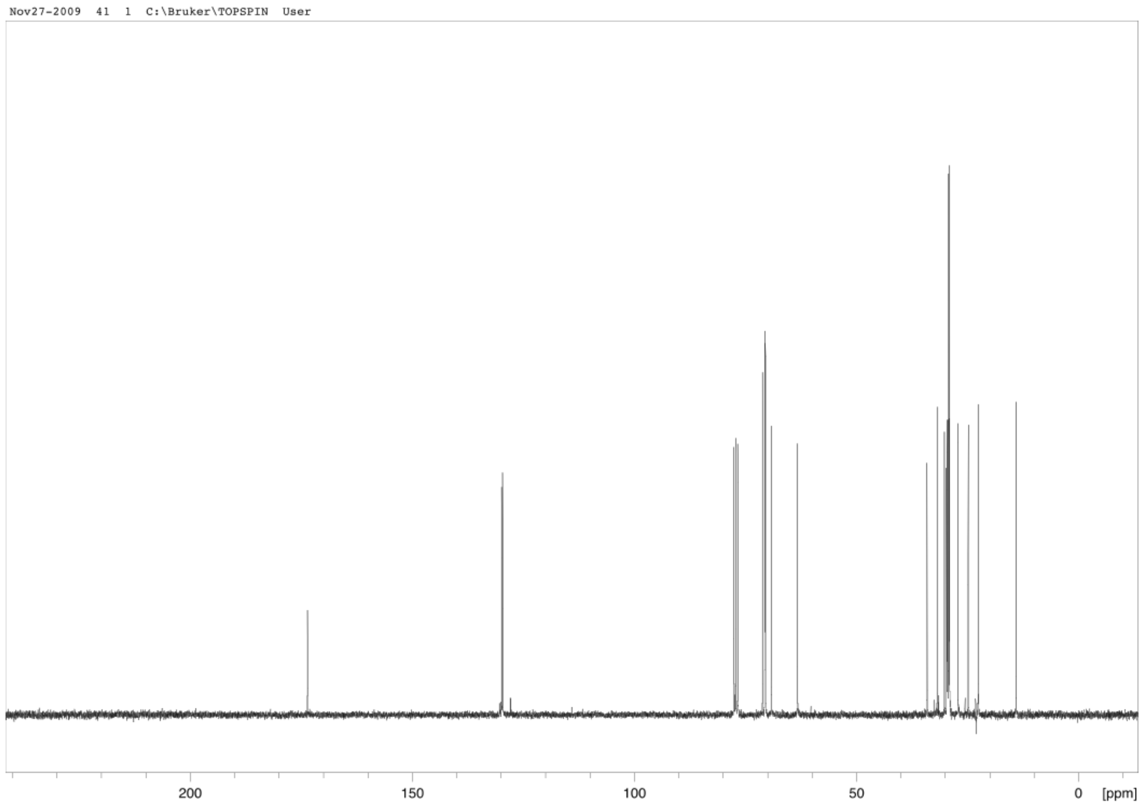

Me-DOesDEG3

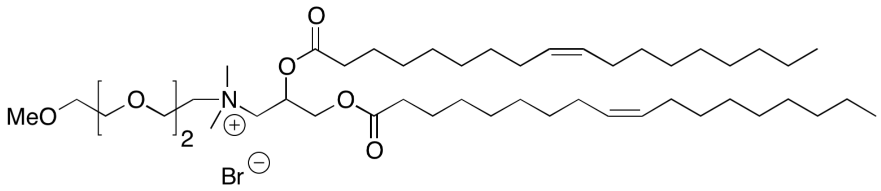

^{1}H NMR spectrum (500 MHz)

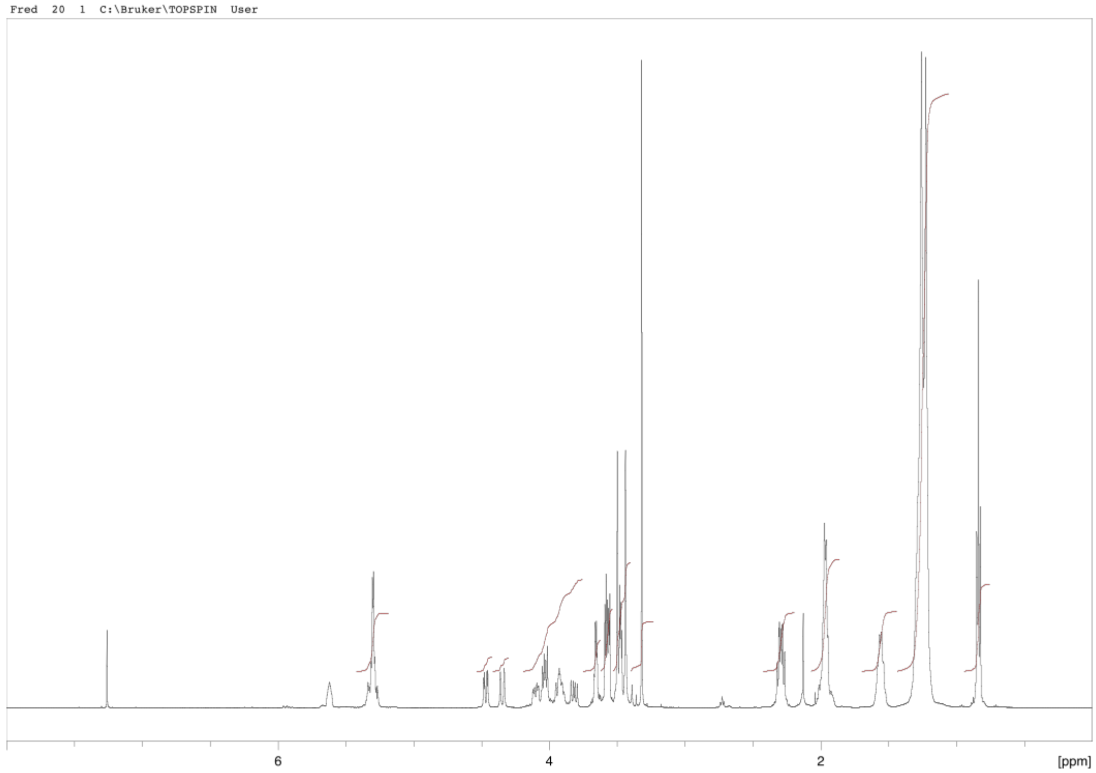

^{13}C NMR spectrum (125 MHz)

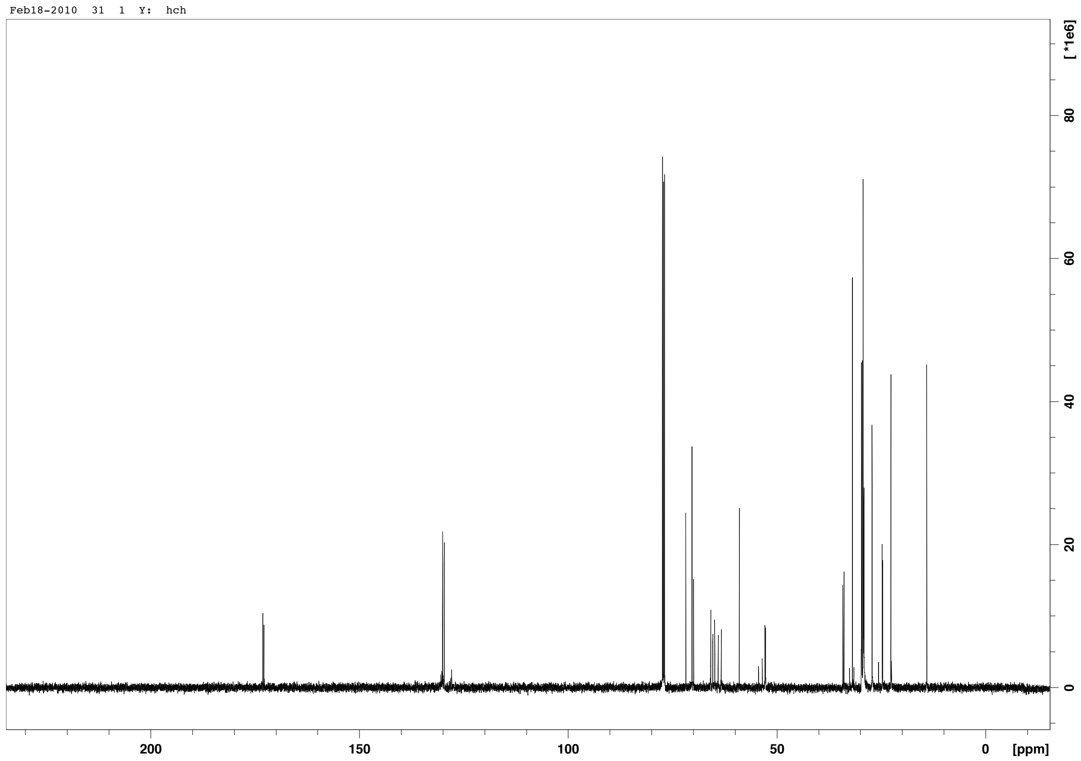

DOesSEG3

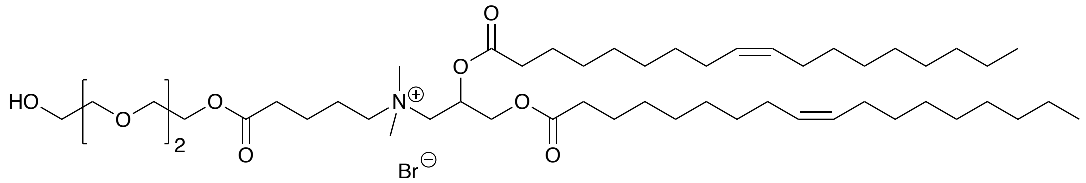

^1H NMR spectrum (600 MHz)

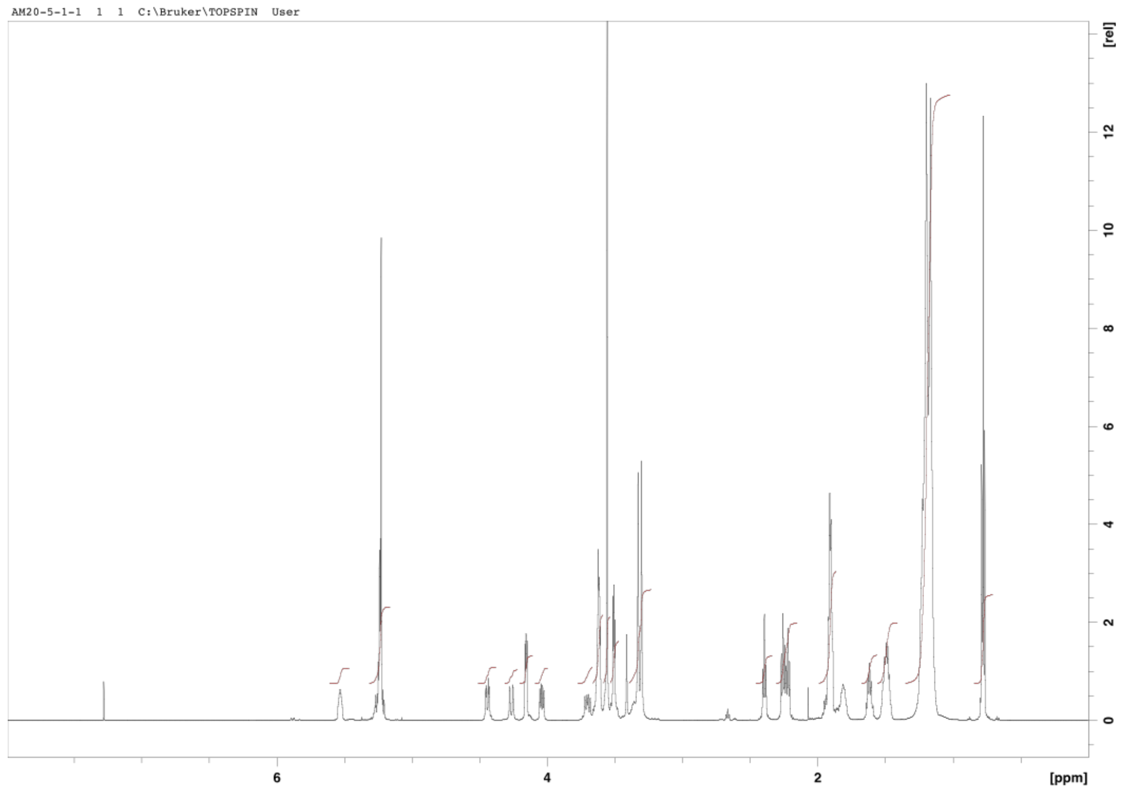

^{13}CNMR spectrum (150 MHz)

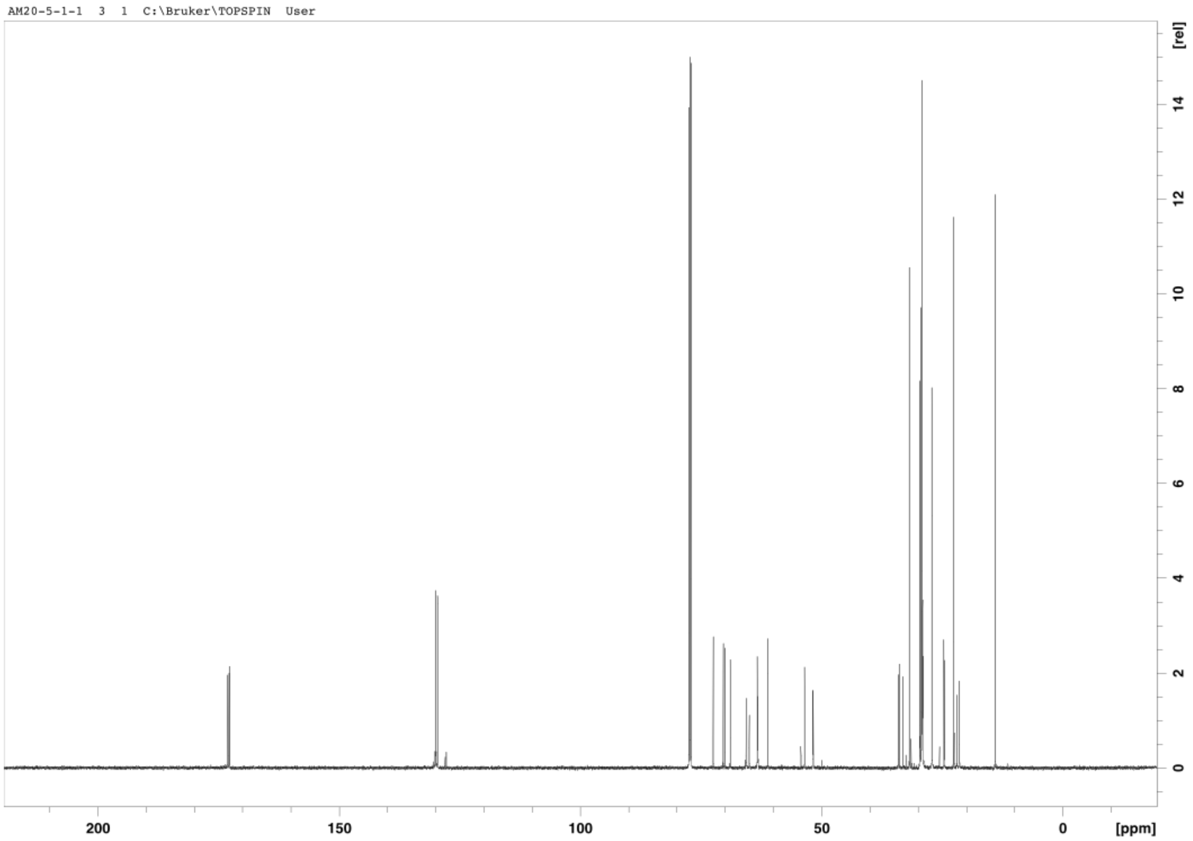

Me-DOesSEG3

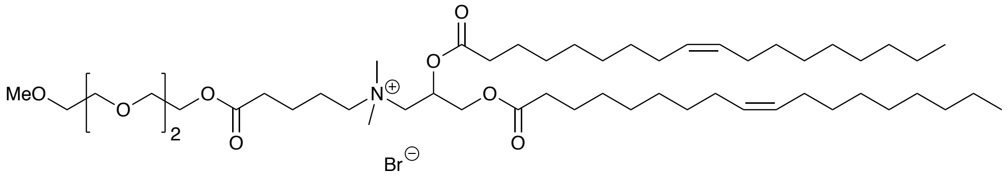

^{1}H NMR spectrum (600 MHz)

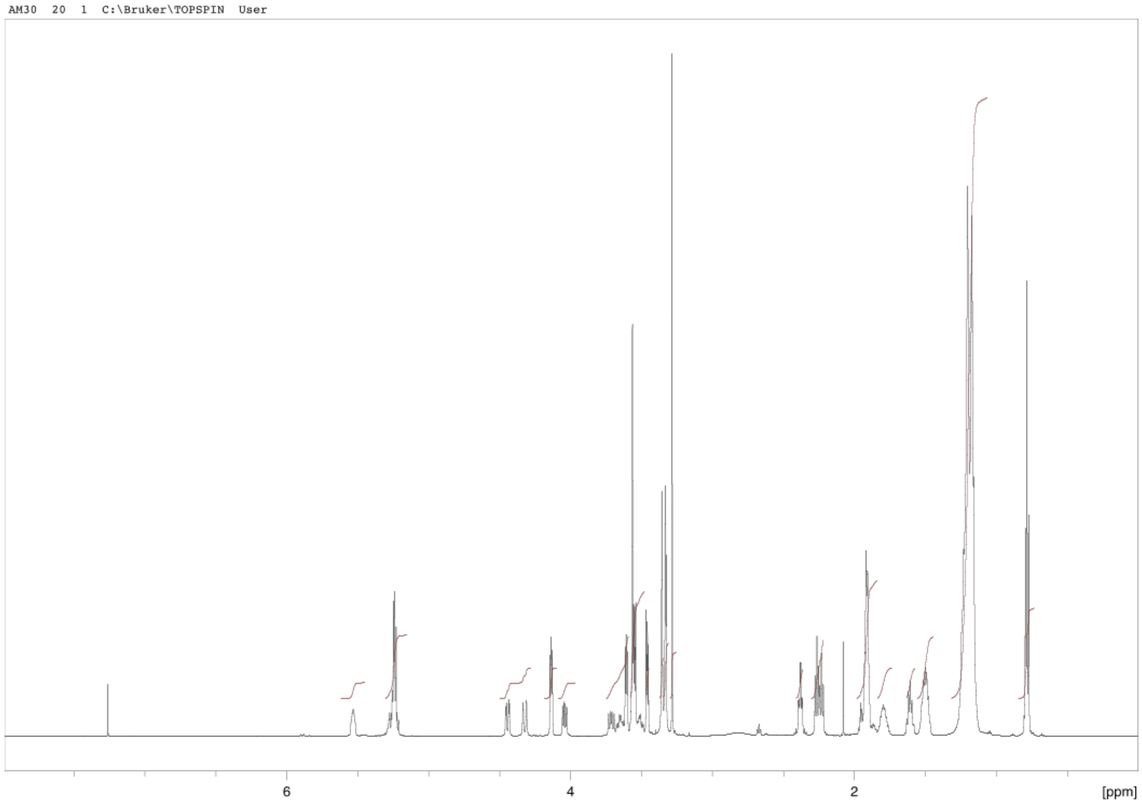

^{13}C NMR spectrum (150 MHz)

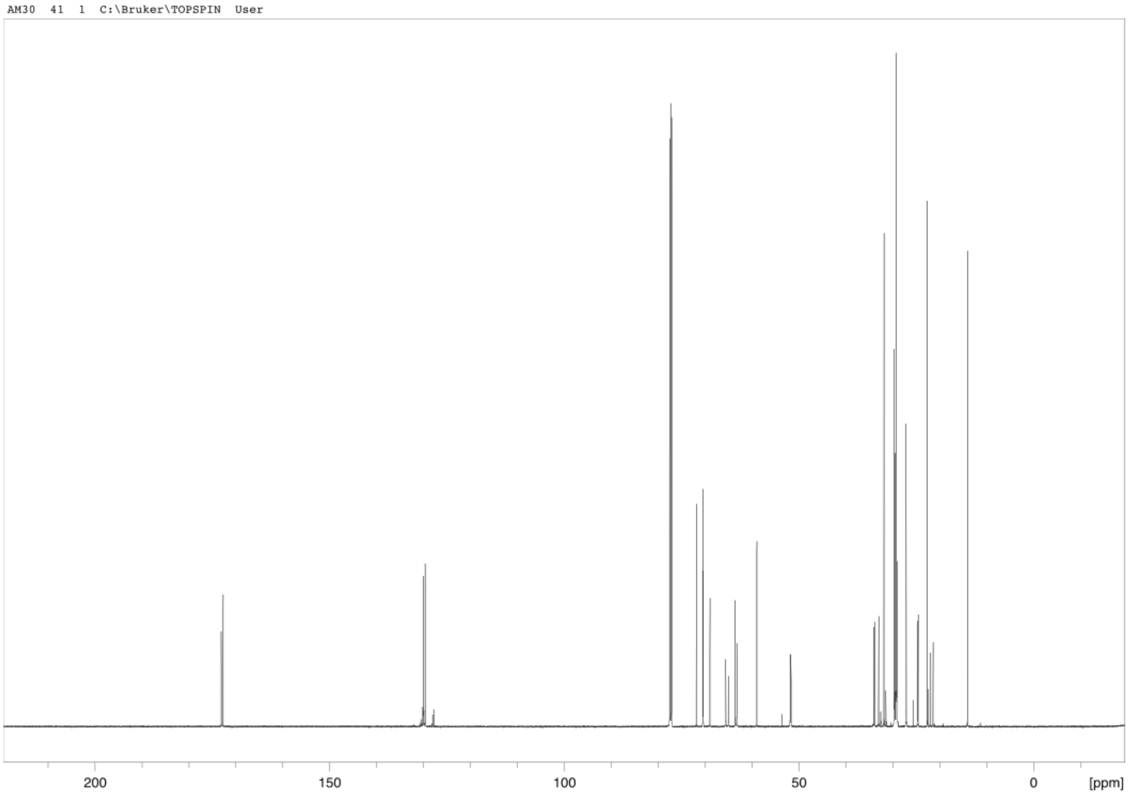

(9*Z*)-2-(2-(2-(5-Bromopentanoyloxy)ethoxy)ethoxy)ethyl oleate

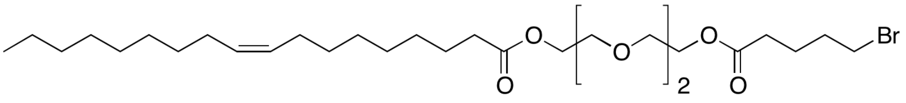

^{1}H NMR spectrum (600 MHz)

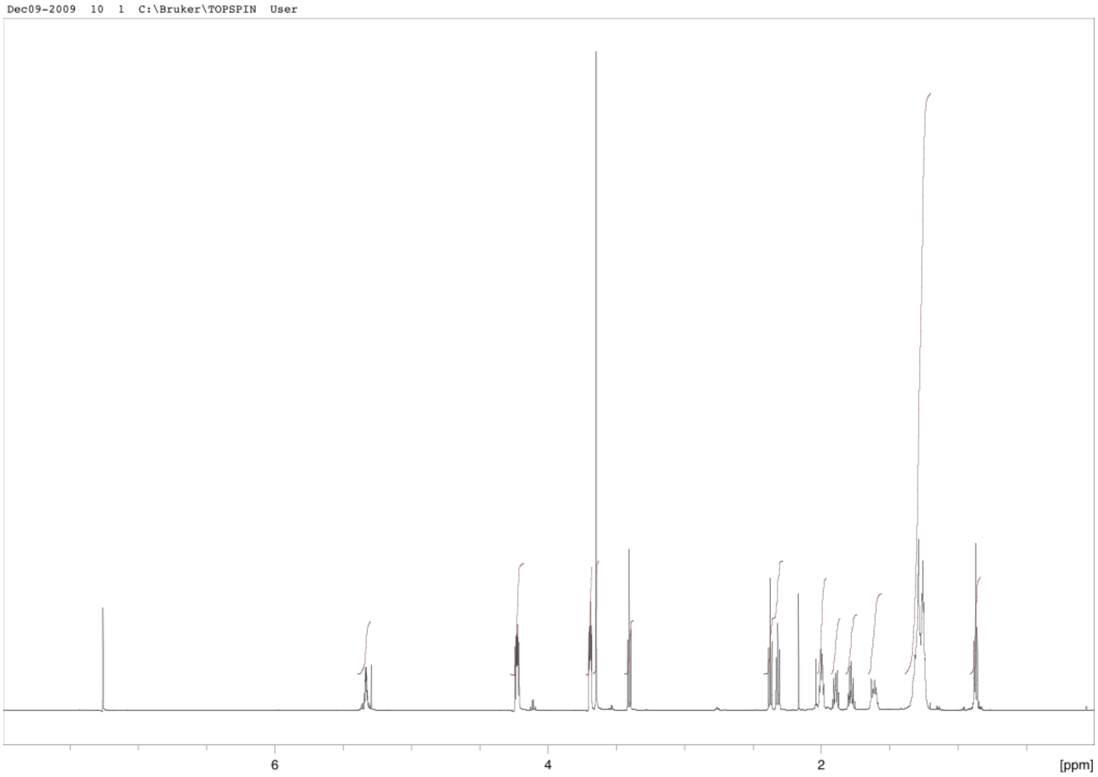

^{13}C NMR spectrum (150 MHz)

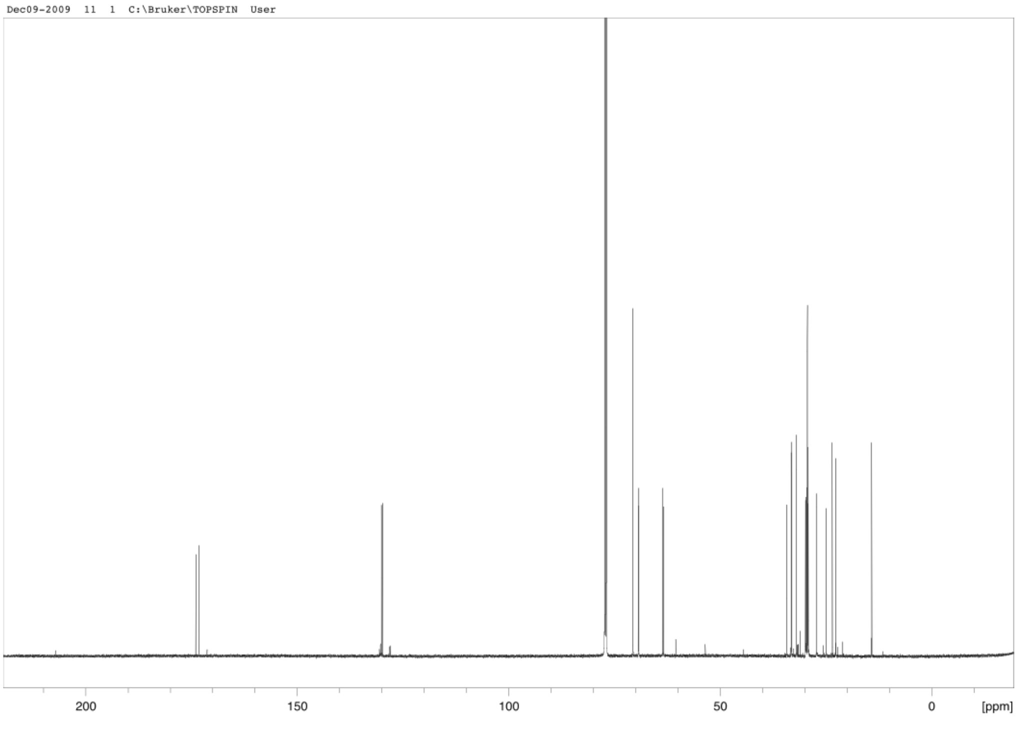

TC-DOSEG3

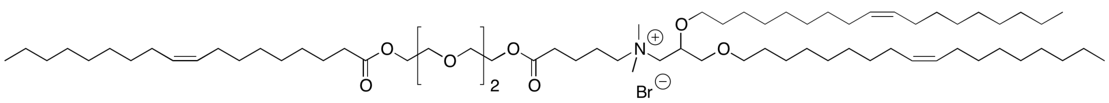

^{1}H NMR spectrum (600 MHz)

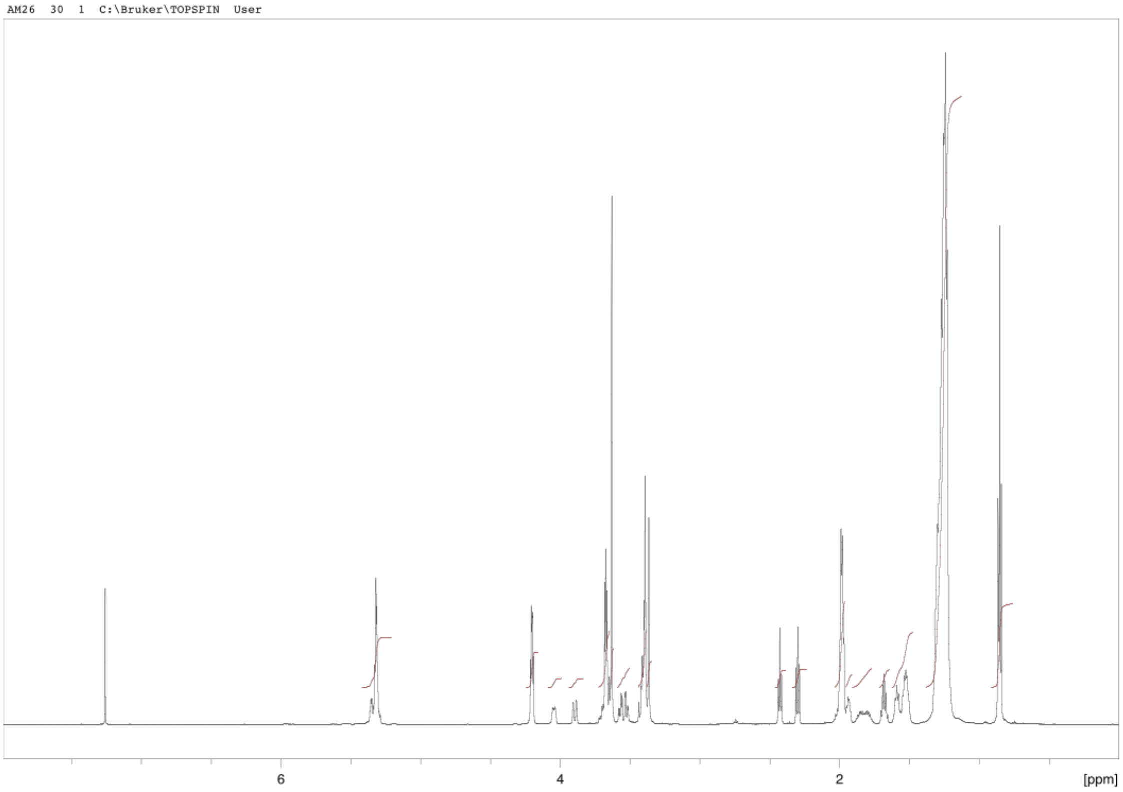

^{13}C NMR spectrum (150 MHz)

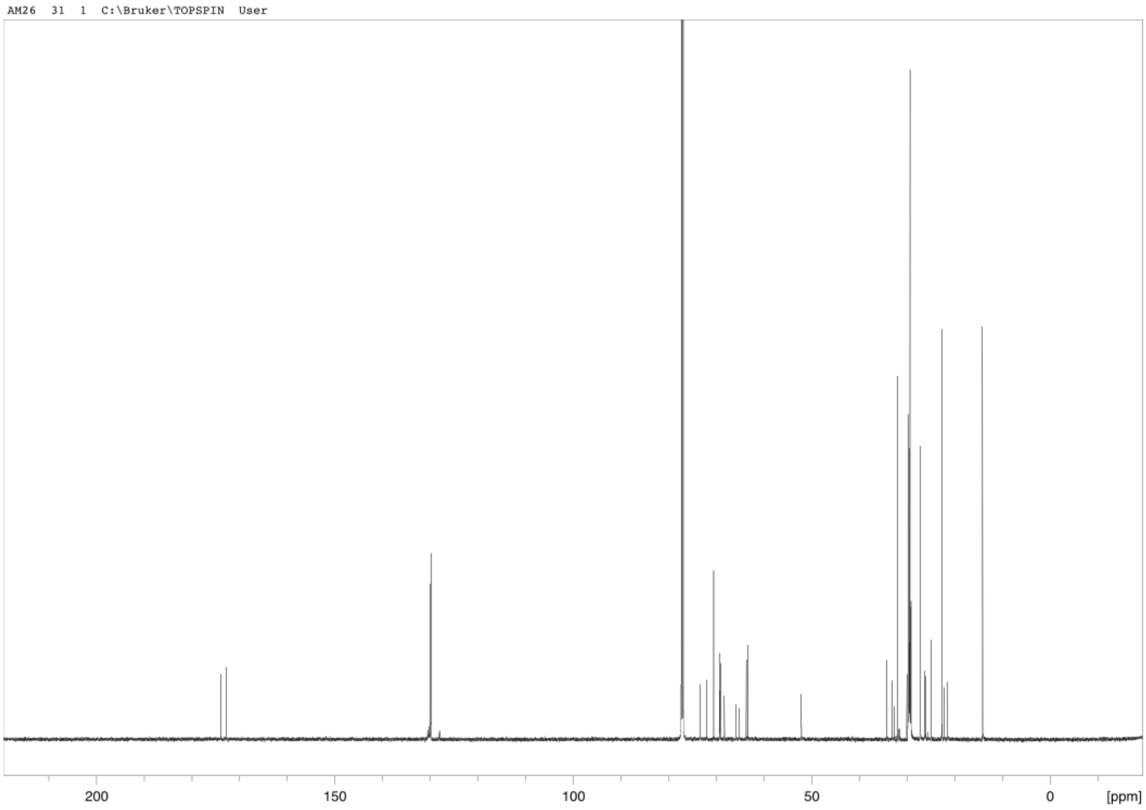

TC-DOesSEG3

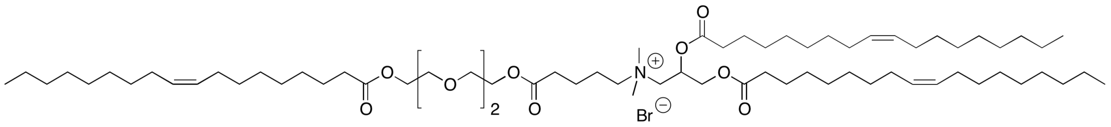

^{1}H NMR spectrum (500 MHz)

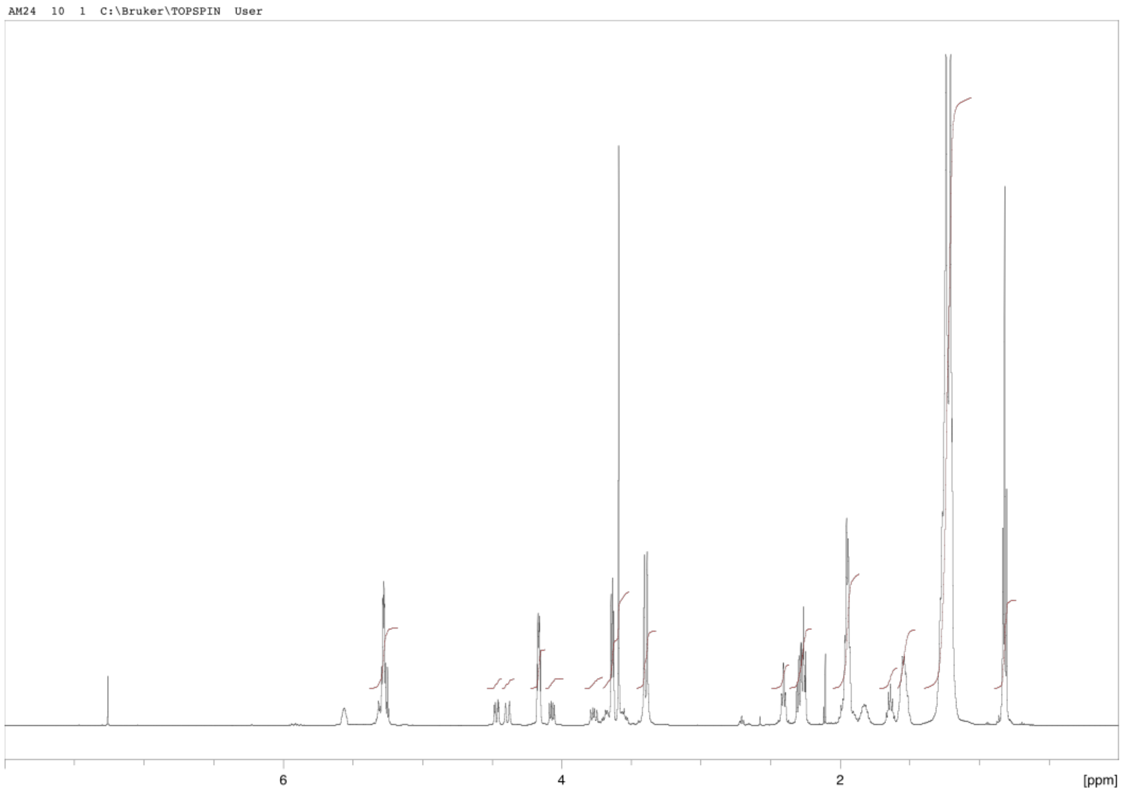

^{13}C NMR spectrum (125 MHz)

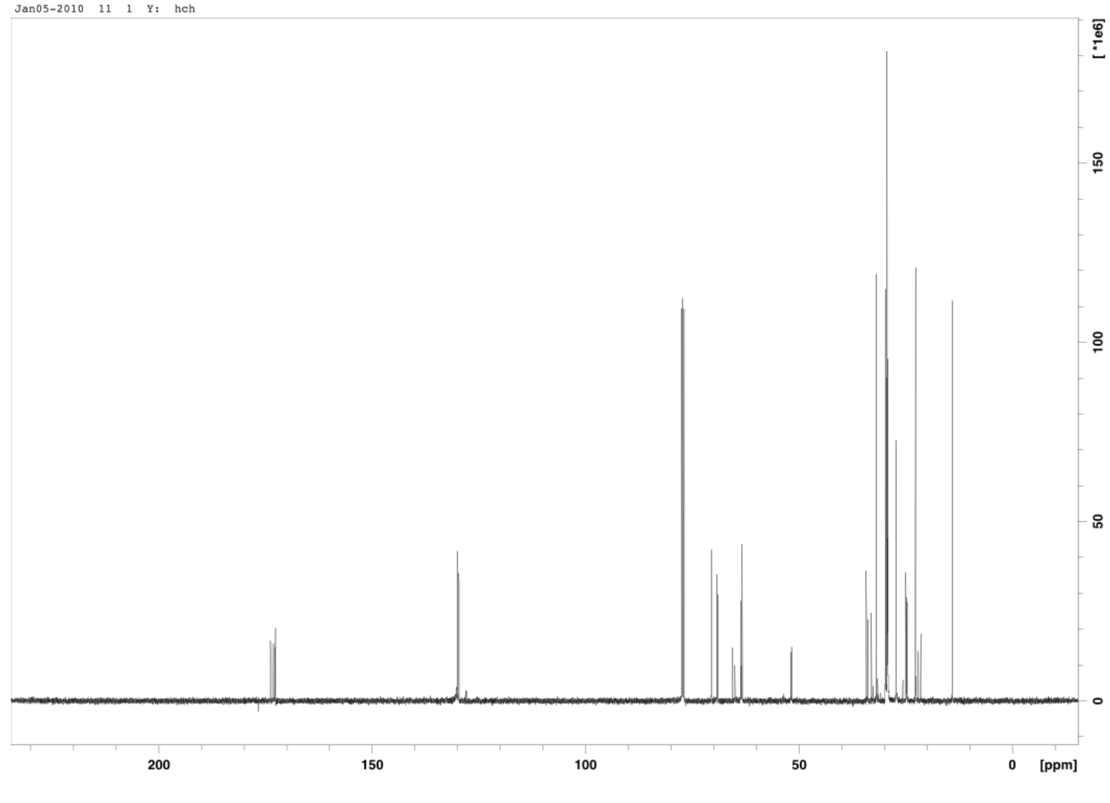

AC-DODEG4

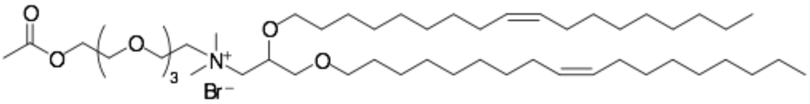

^{1}H NMR spectrum (600 MHz)

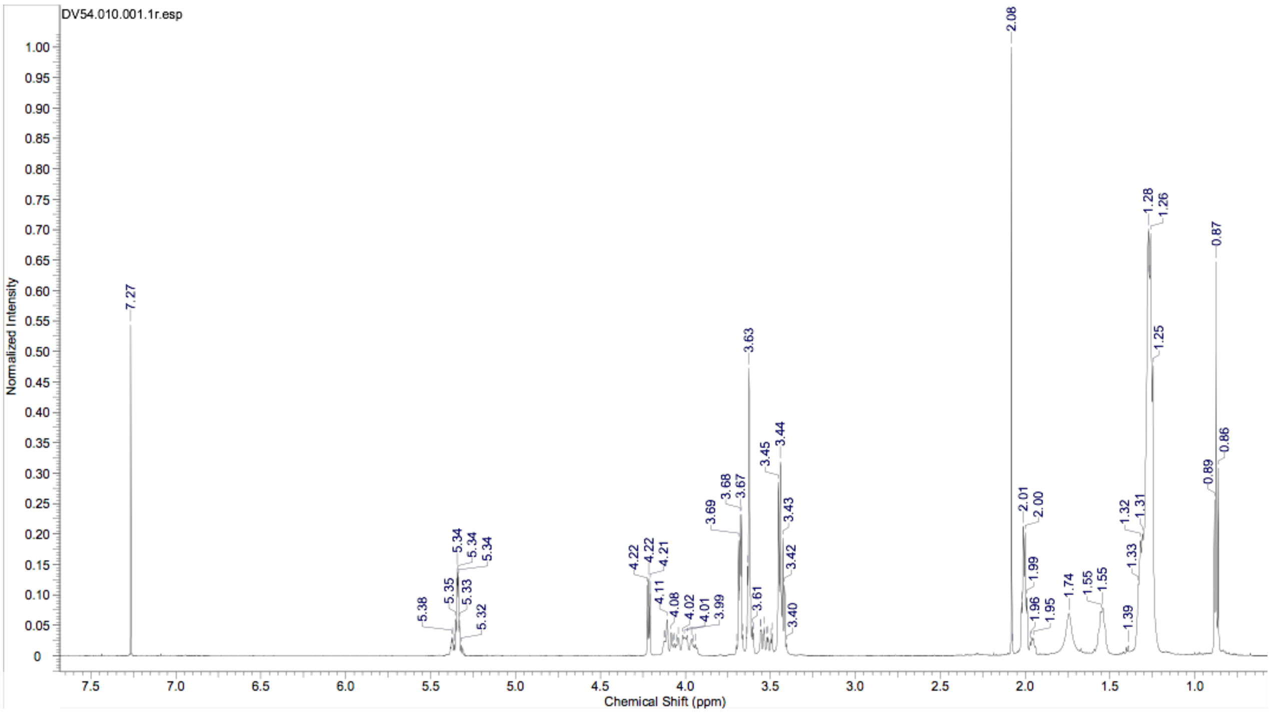

^{13}C NMR spectrum (150 MHz)

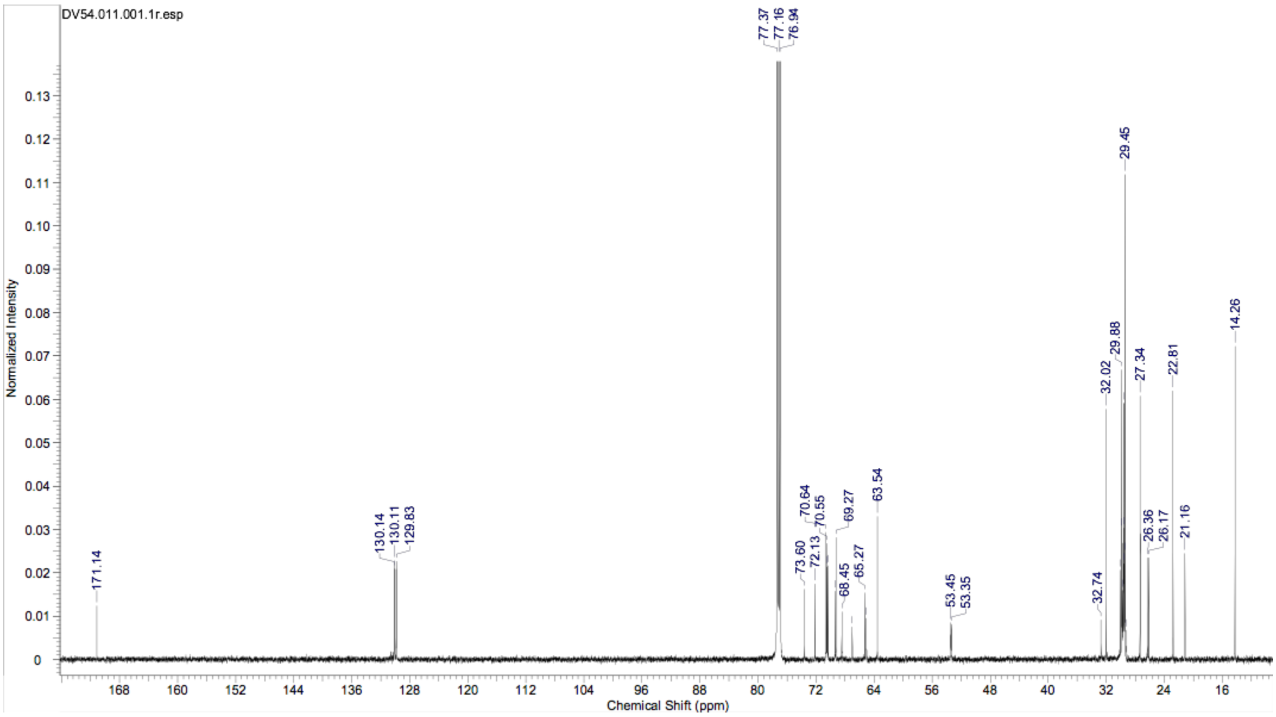

AC-DOesDEG4

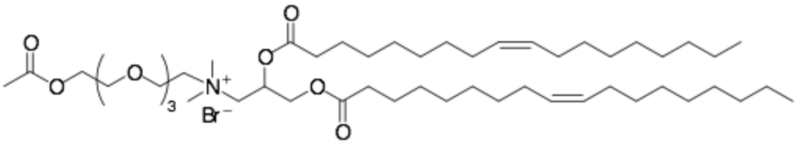

^{1}H NMR spectrum (600 MHz)

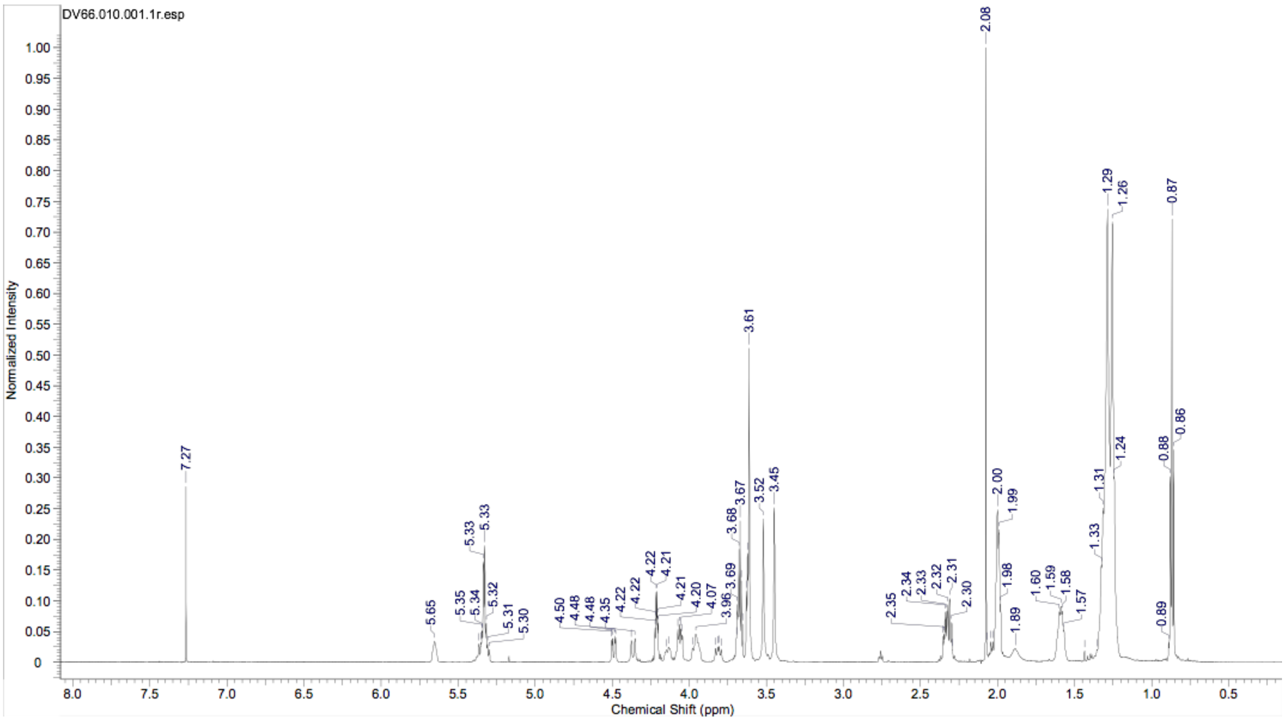

^{13}C NMR spectrum (150 MHz)

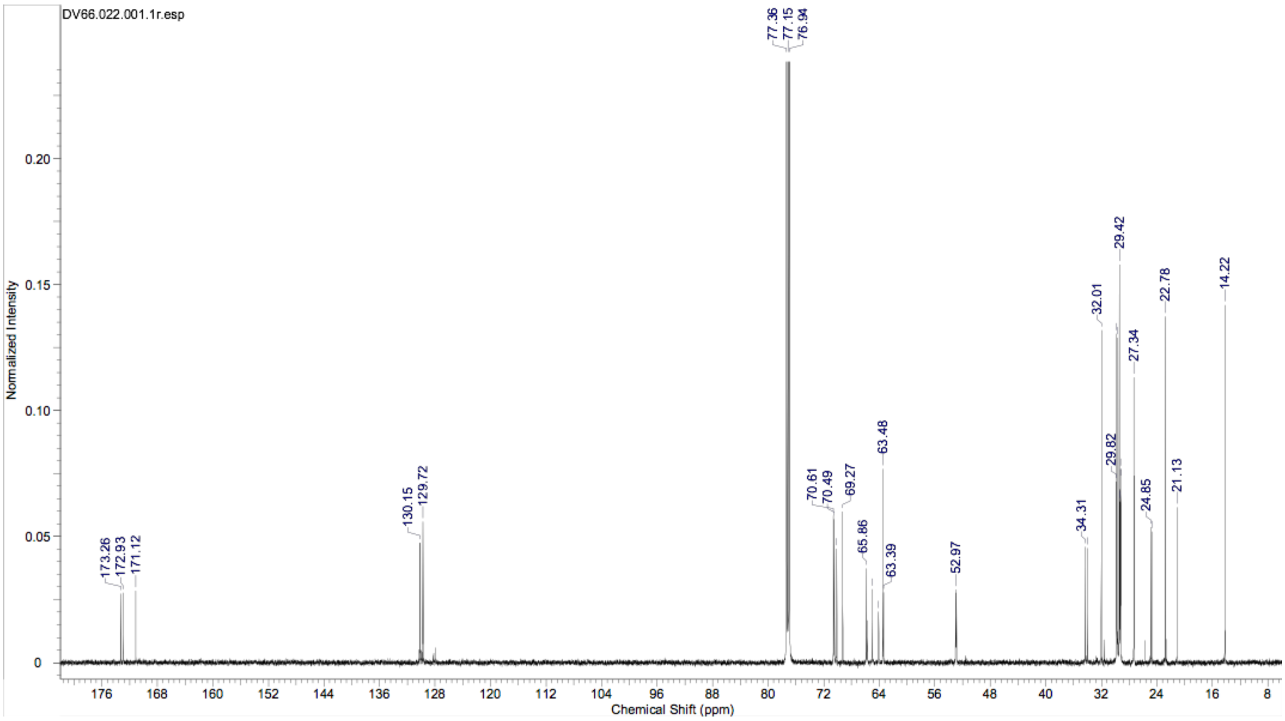

OC-DODEG4

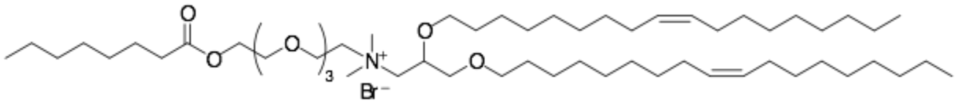

^{1}H NMR spectrum (600 MHz)

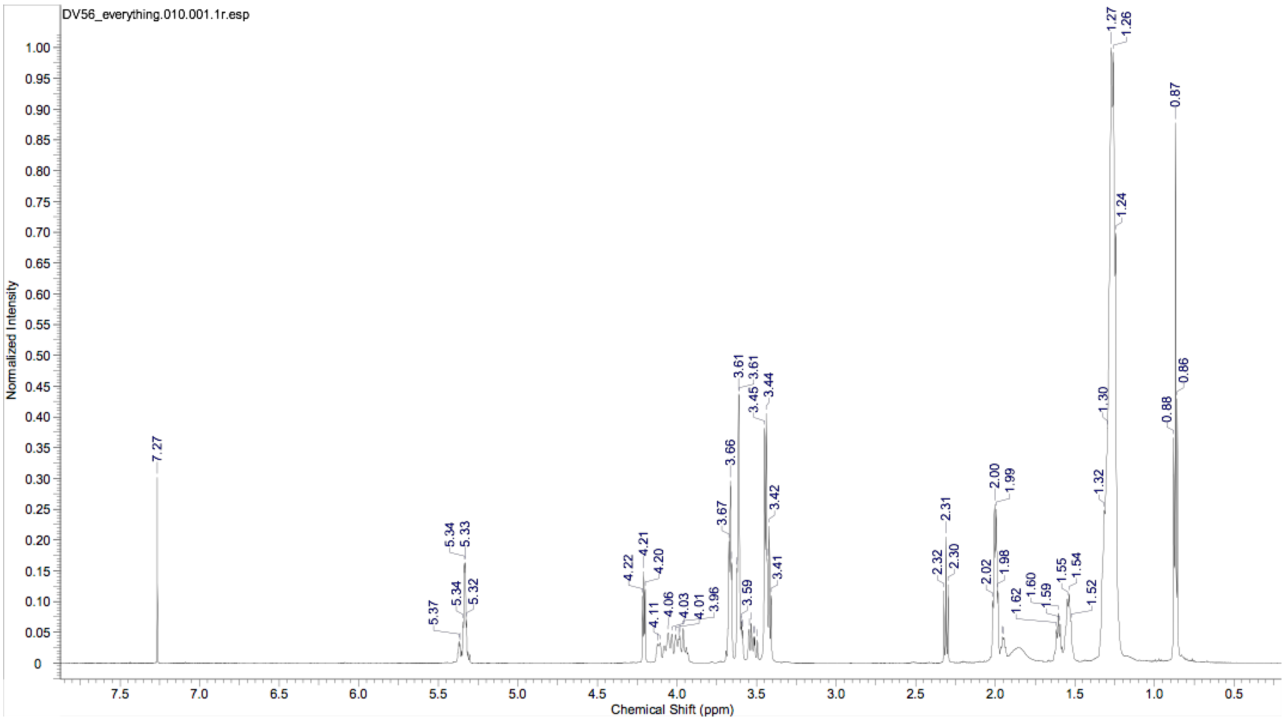

^{13}C NMR spectrum (150 MHz)

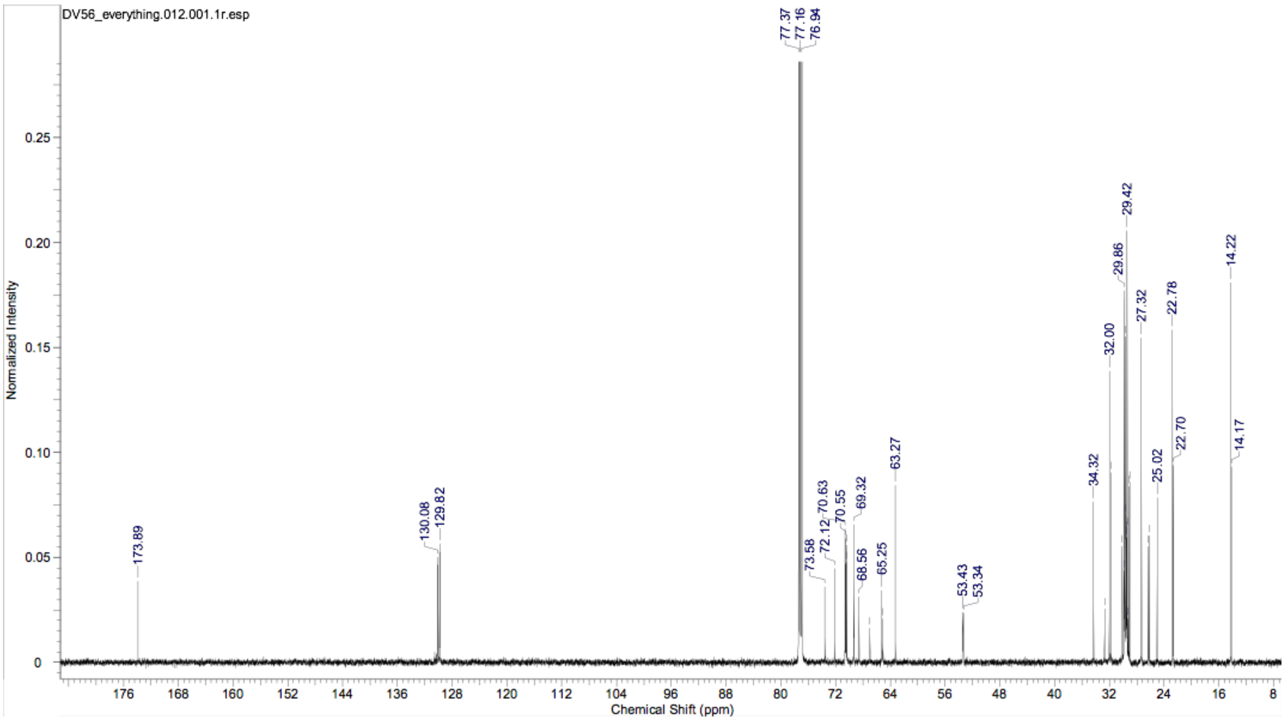

DO-DODEG4

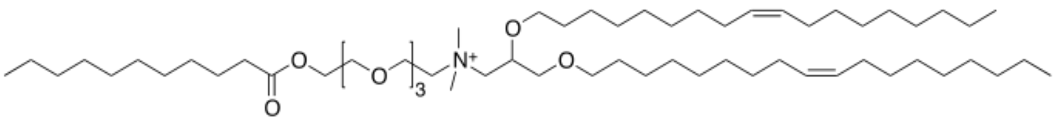

^{1}H NMR spectrum (600 MHz)

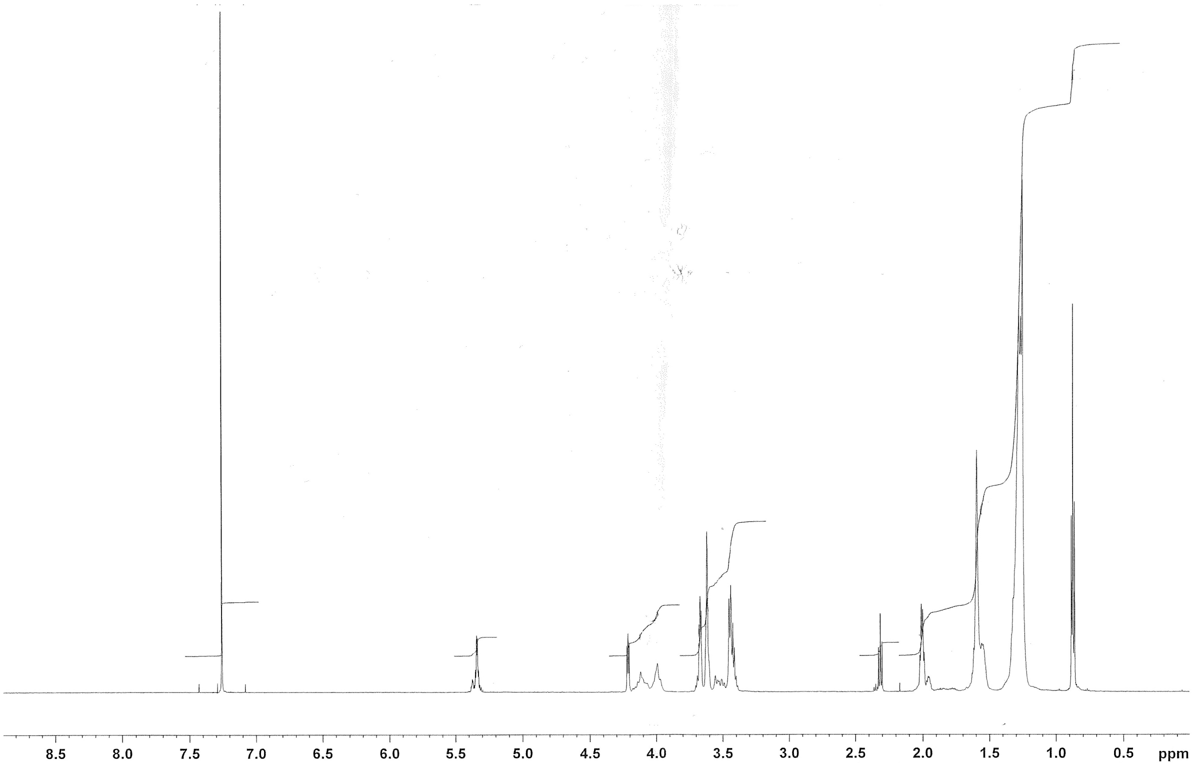

^{13}C NMR spectrum (150 MHz)

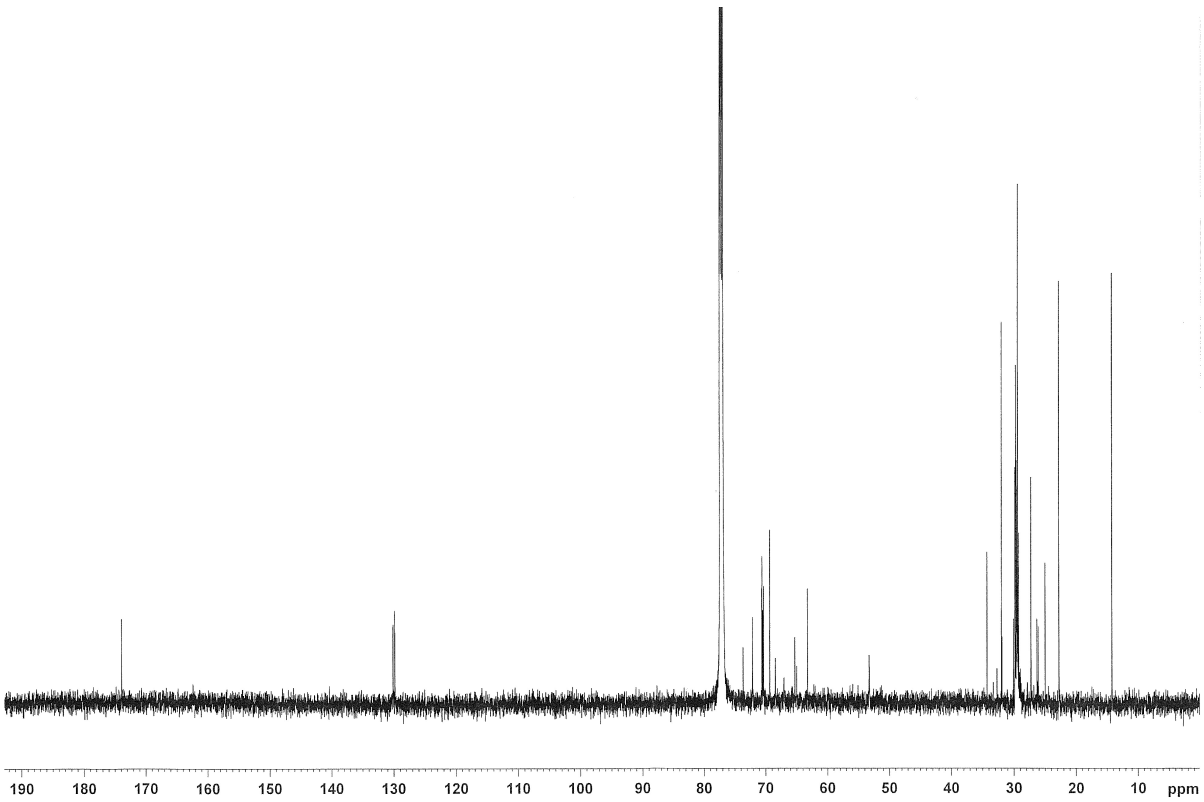

DO-DOesDEG4

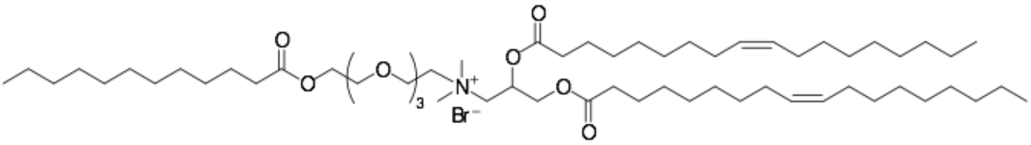

^{1}H NMR spectrum (600 MHz)

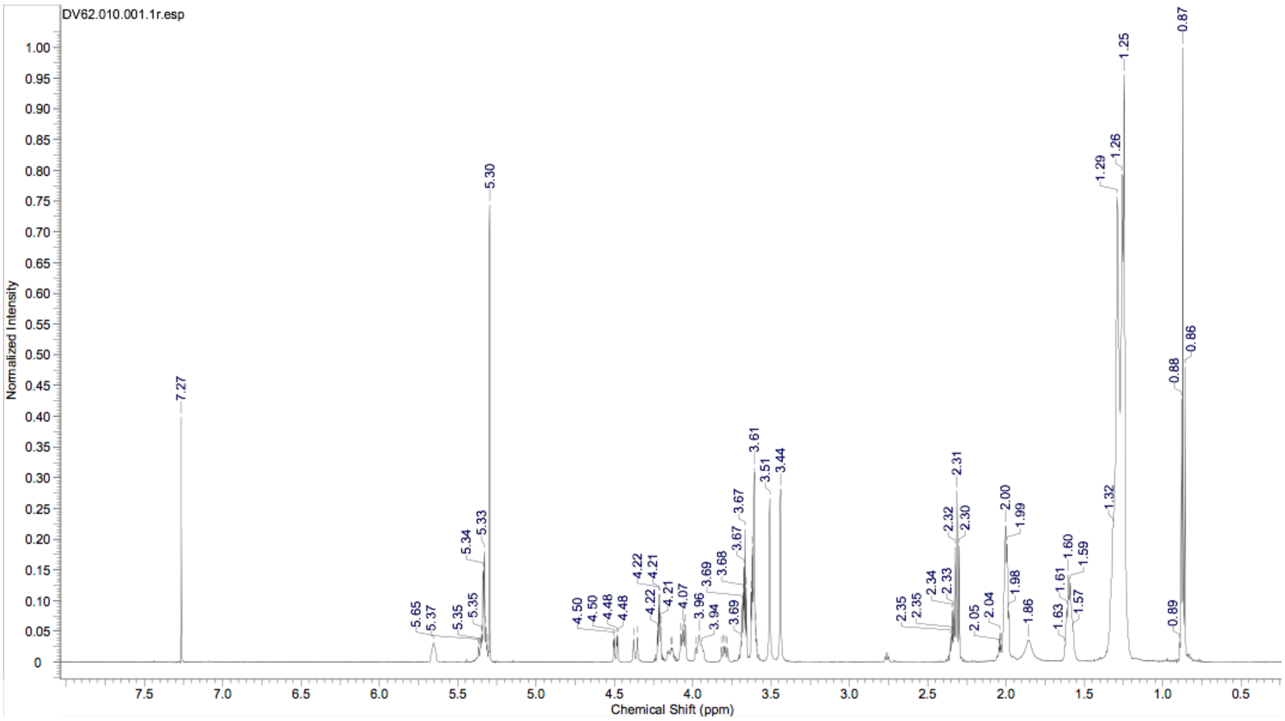

^{13}C NMR spectrum (150 MHz)

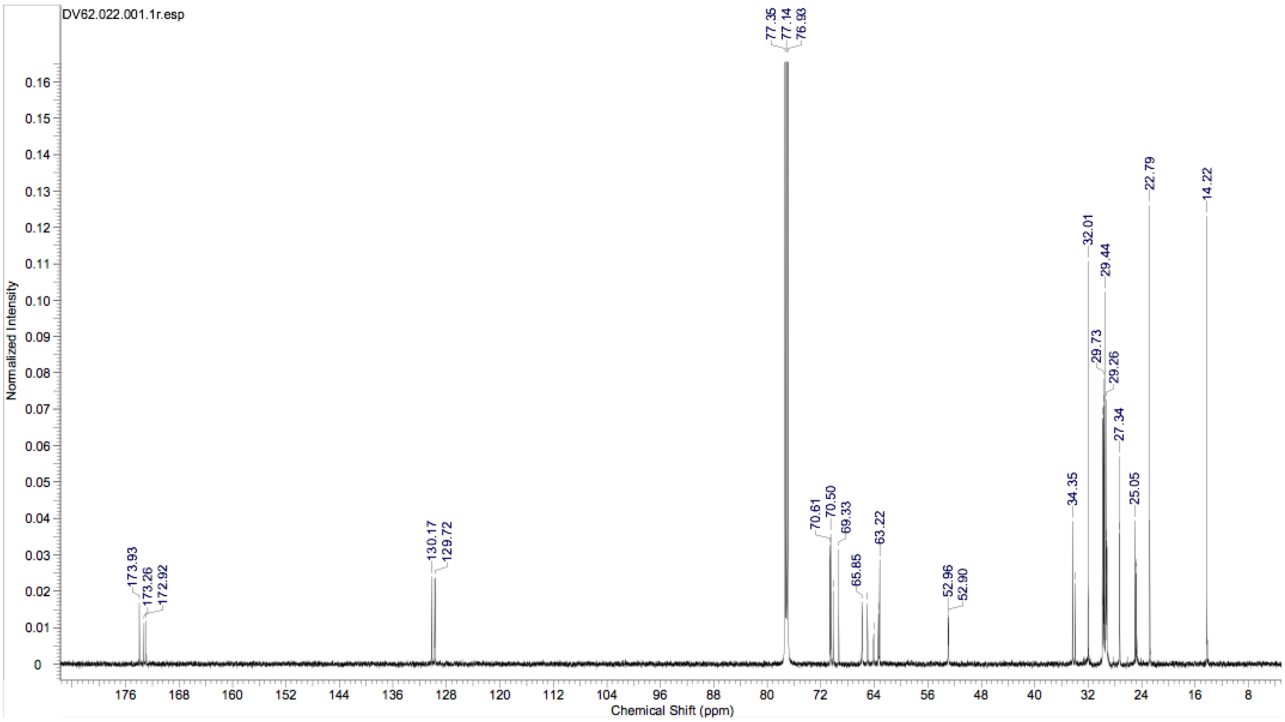

Peptide sc HPLC trace and MS (ES+)

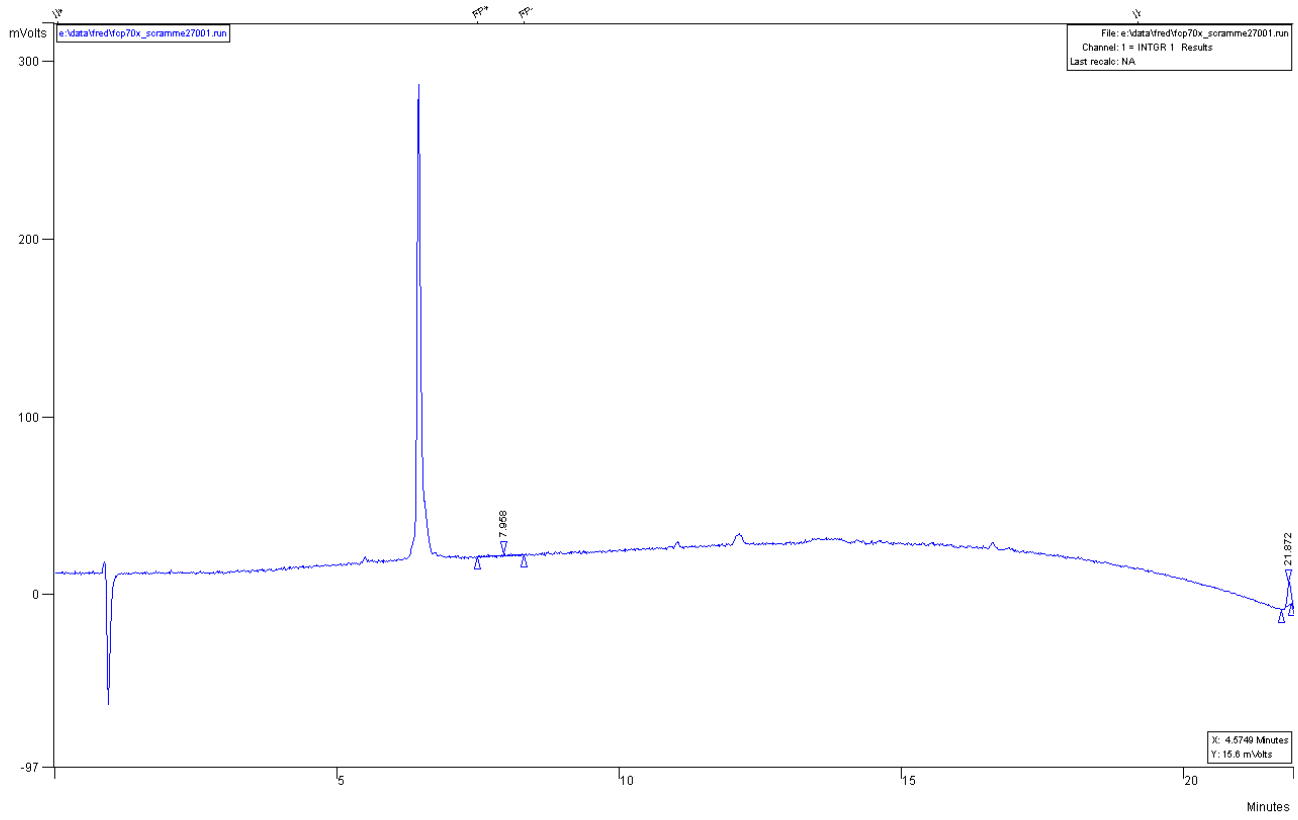

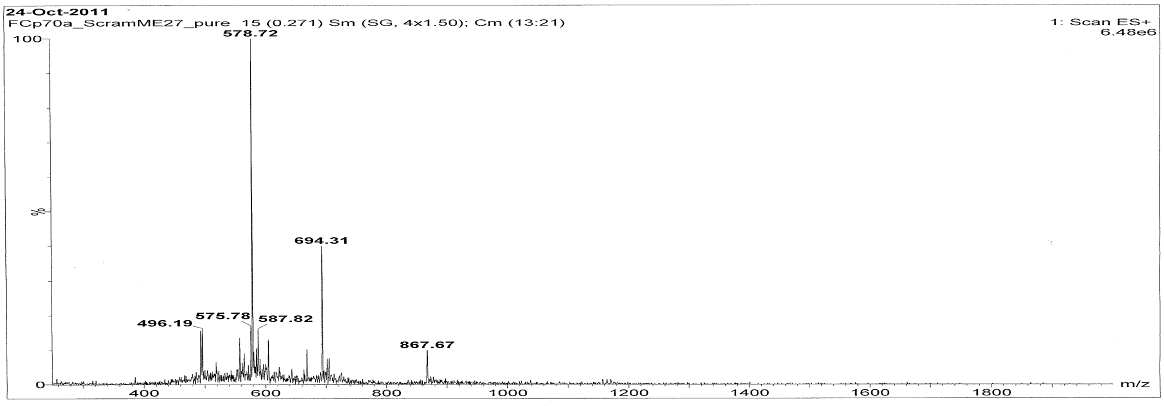

Formulation, transfection and biophysical studies

Lipid transesterification studies

The rate of DODEG4 esterification (dichain to trichain) was measured qualitatively by LC-MS (Waters Acquity LC SQD; *Column*: C18 BEH, 1.9 microns, 50 mm × 2.1 mm). Lipid vesicles, either alone or at a 1:1 molar ratio with DOPE, were prepared as described below using the thin lipid film method (but with bath sonication for 10 min) in HPLC-grade water at pH 7.56 (at 22.3 °C) and pH 4.53 (at 22.1 °C), the latter by careful addition of 1 M HCl to unbuffered HPLC-grade water. Final concentrations of lipid were 1 mg/mL. Solutions were stored at room temperature for the duration of the experiment. For sample analysis, lipid solutions were diluted to a concentration on 0.1 mg/mL in the relevant aqueous solvent.

Single ion monitoring experiments (*Injection volume*: 10 µL; *Gradient*: 5-95% MeCN over 4 min then held at 95% MeCN for 30 s; *Flow rate*: 0.6 mL/min) of 2 and 4 ions were performed respectively for DODEG4 and DODEG4/DOPE solutions. Mass spectra were obtained by summing the scans across the detected peak.

Transfection procedures and biophysical studies

Preparation of vesicles

Cationic di-chain and tri-chain lipids were formulated into vesicles at a 1:1 molar ratio with DOPE (and in some instances lysoPE) and a final cationic lipid concentration of 1 mg/mL (or 2 mg/mL for neutron scattering experiments). The required amount of lipids was weighed into a vial and dissolved in chloroform, and the solvent evaporated *in vacuo* overnight to produce a thin lipid film. The film was then hydrated with the required amount of water (D_2O was used for neutron scattering experiments). The resulting vesicle suspension was then probe sonicated for 5 min using a Lucas-Dawes probe sonicator (model: 7535A) operating at 50% maximum output. The vesicles were then centrifuged at 13,000 rpm (~16,000 g) for 5 min to remove any titanium particles shed from the probe.

Formulation of lipopolyplexes

Unless otherwise stated, lipid:peptide:DNA (LPD) complexes were prepared at 0.25:6.5:1 charge ratios. This was equivalent to approximately 0.75-1:4:1 LPD weight ratios. Previous studies showed that the order of mixing of the three components was crucial for the activity of the complex. The highest transfection efficiency was obtained when peptide was added to the lipid first, at equal volumes to each other, followed by the addition of and an equal volume of DNA to the lipid:peptide

mixture at the intended concentrations.[8] For example, 50 µL of 0.08 mg/mL peptide was added to 50 µL containing 0.75-1 µg of lipid, then 100 µL of 0.1 mg/mL DNA was added to the lipid:peptide mixture.

Dynamic light scattering

1 mL samples of vesicles or PD and LPD complexes, using gWIZ luciferase plasmid (pDNA), were prepared and transferred to a plastic clear-sided cuvette. Size and zeta potential of the complexes were measured by dynamic light scattering using a Zetasizer Nano-ZS series, Malvern Instruments Ltd, UK.

Cell culture

Rat neuroblastoma B104 cells were obtained as a gift from the Institute of Child Health, University College London. The cells were cultured in Dulbecco's modified Eagle's medium (DMEM) containing 4500 mg/L glucose, L-glutamine, sodium pyruvate and sodium hydrogencarbonate and supplemented with 10% v/v fetal bovine serum (FBS) 1% v/v MEM-non-essential amino acids (NEAA) and 1% v/v of 10KU/mL penicillin / 10mg/mL streptomycin solution. Cells were incubated at 37 °C, 5% CO_2 and 90% relative humidity. The cells were passaged every 3-4 days with 0.25% trypsin-EDTA solution when at ~80% confluent.

***In vitro* transfection experiments**

B104 cells were plated into 24-well plates at 7×10^4 cells per well and incubated overnight at 37 °C at 90% relative humidity and 5% CO_2. On the day of the experiment, the DMEM growth media was removed and the cells rinsed with 200 µL of OptiMEM, which was removed and another 200 µL of OptiMEM added. 200 µL of LPD sample was then added to each well in triplicate so that the final concentration of pDNA in each well was 1 µg per 400 µL per well. The samples were incubated at 37 °C, 5% CO_2 and 90% humidity for 4 h after which the well contents were removed and replaced with 1 mL of fresh DMEM growth media. The plates were then incubated for 48 h before measuring luciferase expression.

LPDs were prepared using two different methods. One method involved formulating the LPDs in OptiMEM, therefore the lipid, peptide and pDNA were diluted with OptiMEM first then mixed together at a final pDNA concentration of 1 µg per 200 µL, which was then added to a well containing 200 µL of OptiMEM. The second method involved formulating the LPDs in water first then diluting with OptiMEM. The LPDs were formulated in water at double the concentration so that the final pDNA content was 1 µg per 100 µL, to which 100 µL of OptiMEM was added before transferring into

a well containing 200 µL of OptiMEM. Therefore, the content of water in these samples was 25% v/v and this was shown not to affect cell viability at those experimental conditions. Lipofectamine and Lipofectamine 2000 (L2K) at 5:1 and 4:1 weight ratios with pDNA were used as positive controls. Untreated cells were used as a negative control.

Luciferase activity was measured using a luciferase assay kit (Promega, UK) according to the manufacturer's protocols. The cells were lysed with lysis buffer then 20 µL of the lysate was transferred into a white 96-well plate and the luminescence measured using an MLX Microtiter Plate Luminometer (Dynex Technologies, Chantilly, USA) with an automatic feeding system delivering 100 µL of the reconstituted luciferase assay reagent into each well and measuring for 10 s. Luciferase activity was expressed as Relative Light Units (RLU) per mg of protein (RLU/mg protein).

The amount of protein in each transfection lysate was also measured using a BCA protein assay (Thermo Scientific, UK) according to manufacturer's instructions. Briefly, 20 µL of the cell lysate and 20 µL of bovine serum albumin (BSA) protein standards were transferred into a transparent 96-well plate to which 200 µL of the mix reagent A/B (provided in the kit) was added. The plate was incubated with slight shaking at 37 °C for 30 min, after which time the absorbance was measured at 562 nm using a SpectraMax 190 plate reader (Molecular Device, USA).

All measurements were carried out in triplicate, and the error bars represent the standard deviation calculated from three different measurements carried out in one experiment.

Gel retardation, release and protection assay

The binding, release and protection of pDNA from DNAse I degradation that the LPD complexes afforded was determined using agarose gel electrophoresis. LPD complexes were prepared at a final pDNA concentration of 0.25 µg per 10 µL per well at 0.25:6.5:1 LPD charge ratios. To determine pDNA binding efficiency, 10 µL of the LPD complex without further treatment was loaded into the first well. To measure the degradation of the pDNA in the complex, 0.5 µL of 0.1 M $MgCl_2$ was added to a 10 µL LPD sample followed by the addition of 0.5 µL of either 500 or 1000 U/mL of DNAse I. The samples were incubated at 37 °C for 10 minutes after which the reaction was stopped by the addition of 1.5 µL of 0.5 M EDTA solution. Finally, 0.4 µL of highly anionic poly-aspartic acid (pAsp) at 10 mg/mL was added to release any protected pDNA and visualise it on the gel. As a control to test the extent of pDNA release from the complex in the presence of pAsp, and the absence of DNAse I, 0.4 µL of 10 mg/mL pAsp was added to 10 µL of the LPD sample. Free pDNA and pDNA treated with DNase I were used as controls. To all the samples 2 µL of loading buffer was added before loading into wells in a 0.8% w/v agarose gel containing GelRed in tris-acetate-edetate (TAE) buffer at pH 7.4

and run at 80 mV for one hour. The gel was visualized using a Herolab EASY UV transilluminator. The intensity of the pDNA bands were analysed using GelAnalyzer program.

Transmission electron microscopy

LPD complexes were prepared at 0.25:6.5:1 charge ratios at a final volume of 50 µL and a final pDNA concentration of 0.02 mg/mL. A drop of sample was placed on a Formavar 200 mesh copper grid for a few minutes, after which the sample was dried using a filter paper. The sample was stained by placing the grid sample face down onto a drop of 4% uranyl acetate solution for several minutes. The grid was then washed in ethanol and water and left to dry before visualisation on an FEI Tecnai T12 transmission electron microscope, USA.

Small angle neutron scattering

Small angle neutron scattering (SANS) measurements on the parent lipid dispersions in water and on the LPD complexes were performed using the beam line, LoQ, at the ISIS pulsed neutron source (STFC Rutherford-Appleton Laboratory, Didcot, U.K). All samples were freshly made using deuterium oxide (D_2O). Lipid vesicles were measured at 1 mg/mL of cationic lipid (approximating to 1.6-2 mg/mL of total lipid depending upon the molecular weight of the cationic lipid) whereas LPD complexes were formulated so that the final DNA concentration was generally 0.0625 mg/mL.

The complexes were formulated at 0.25:6.5:1 charge ratio with both peptide **A** and **K16** and calf thymus DNA (ctDNA) instead of pDNA. In addition, several LPDs prepared using either **K16** or peptide **A** were also investigated at a final ctDNA concentration of 0.15 mg/mL. Previous similar SANS studies showed no difference in the complex structure when ctDNA or pDNA were used.[8] Samples were placed in clean, disc-shaped fused silica cells of 2 mm path length and measured at 25 °C. The SANS intensity, I(Q), of the LPDs and vesicles as a function of the scattering vector, $Q = (4\pi/\lambda)\sin(\theta/2)$, where $\theta/2$ is the scattering angle, was determined by normalising the scattering to the appropriate sample transmission after subtraction of the scattering from the relevant solvent also normalised to its corresponding transmission. The fitting of the SANS data always included flat background corrections to allow for any mismatch in the incoherent and inelastic scattering between the sample and the solvent, with the levels of the fitted background being checked to ensure that they were physically reasonable. The SANS data for the DOTMA:DOPE vesicles and LPR complexes dispersed in D_2O were routinely modeled either assuming a mixture of (isolated/single) infinite planar (lamellar) sheets with or without one-dimensional paracrystals (stacks) to account for the presence in the sample of any multilamellar vesicles. When modeling the vesicles and LPR complexes dispersed in D_2O as (single) lamellar sheets, the fits to the SANS data were obtained by the least-squares

refinement of three parameters, namely L, Rσ, and the absolute scale factor (together with the background, as described above), where Rσ is the Lorentz correction factor which provides information about the extent of rigidity/curvature of the lamellar sheets. In this study the polydispersity on the thickness of the bilayer (σ(L)/L) was fixed at 10-6. When stacks were added to the (single) lamellar sheet model, the fit to the SANS data was obtained by least-squares refinement of seven parameters, namely, the mean bilayer thickness (L), the Lorentz factor (Rσ), the number of bilayers in the stack (M), their mean separation or d-spacing (D), the width of the Gaussian distribution in the plane, (σ(D)/D), and the absolute scale factors for the unilamellar and multilamellar vesicles. Where a Bragg peak was clearly observed in the data, it was fitted using bilayers of 5 and 10. In the present study, σ(L)/L was again fixed as 0.1, σ(D)/D at 0.05, and Rσ at 300. In addition, when modeling the SANS data using a mixed population of sheets and stacks, L, σ(L)/L, and Rσ were constrained to be the same for the isolated/single and stacked lamellae, a not unreasonable assumption. If no Bragg peak was seen in the SANS data, it was fitted using a stack with a maximum of 2 bilayers. In such cases the data was fitted using a higher number of bilayers comprising the stacks to ensure that it did not improve the quality of the fit obtained. For all models, the least-squares refinements were performed using the model-fitting routines provided in the FISH[9] software.

Circular dichroism

The circular dichroism (CD) spectra of LPD complexes prepared in D_2O (used because of its better optical transparency than H_2O) was measured using a Chirascan Plus spectrophotometer (Applied Photophysics, UK) at 20 °C, a scan speed of 30 nm/min, bandwidth of 1 nm and time per point of 2 sec. Complexes were prepared using the di-chain lipid DOSEG3 and the equivalent tri-chain lipid TC-DOSEG3 formulated with either the targeting peptide **A** or non-targeted **K16** peptide. All LPDs were prepared at 0.25:6.5:1 charge ratio and a final ctDNA concentration of 0.05 mg/mL. Spectra were obtained at 200-320 nm and 20 ^{o}C using a scan speed of 30 nm/min using a 0.5 cm path length cell. Appropriate D_2O background subtraction was performed for all spectra. The spectra of free ctDNA, lipid vesicles alone, and peptides alone were also obtained as a reference.

Figures: Supplementary information

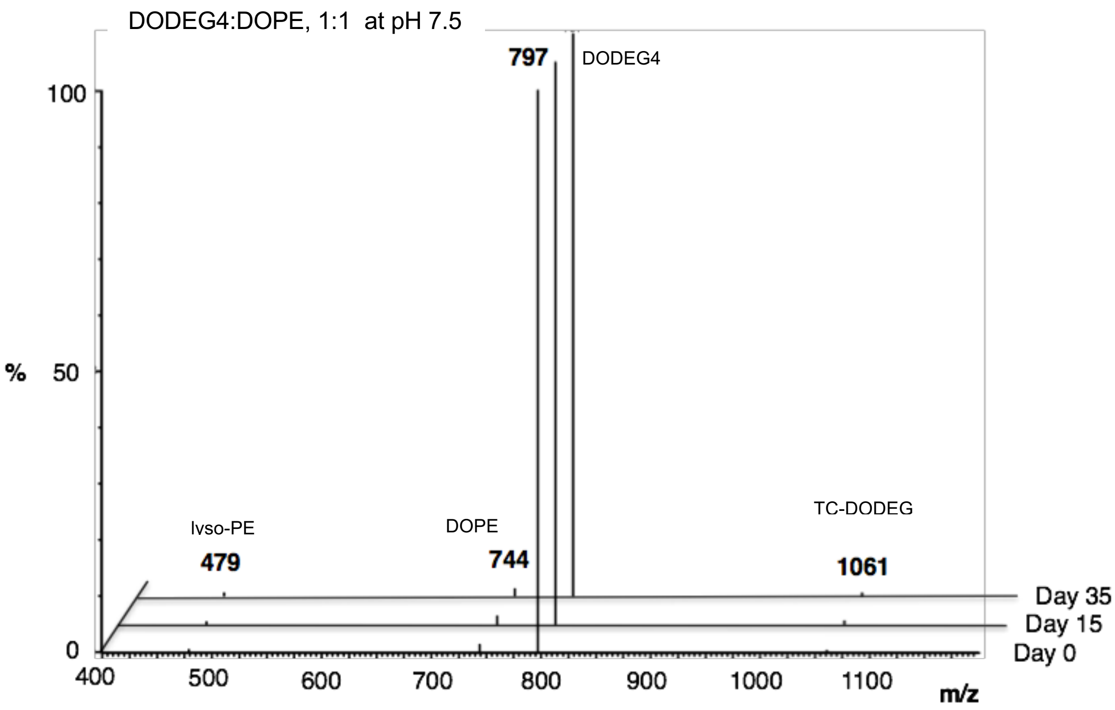

Figure S1. Formation of TC-DODEG4 and lyso-PE (**1** and **2**) from DODEG4 and DOPE at pH 7.5, monitored by LC-MS.

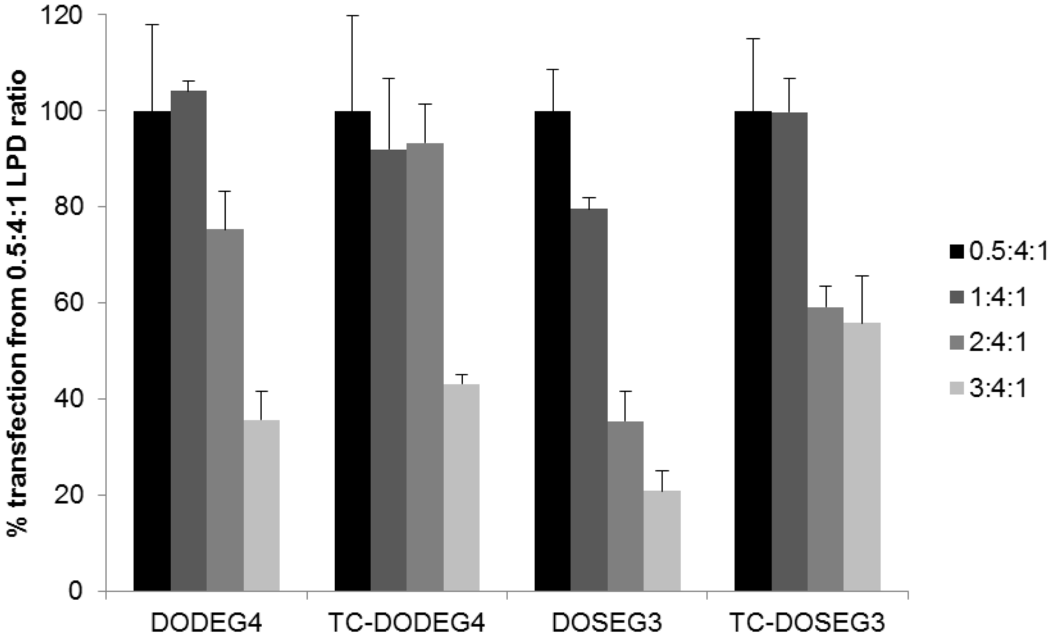

Figure S2. % transfection in B104 cells of LPDs prepared using the lipids DODEG4, TC-DODEG4, DOSEG3 and TC-DOSEG3 with peptide **A** and pDNA at increasing lipid ratios in the LPD complex from 0.5:4:1 to 3:4:1 weight ratios. The 0.25:6.5:1 charge ratio chosen for subsequent experiments is equivalent to 0.7-1:4:1 weight ratio, and therefore corresponds to the range in which highest transfection efficiency was observed. Data is the mean of three measurements ± standard deviation.

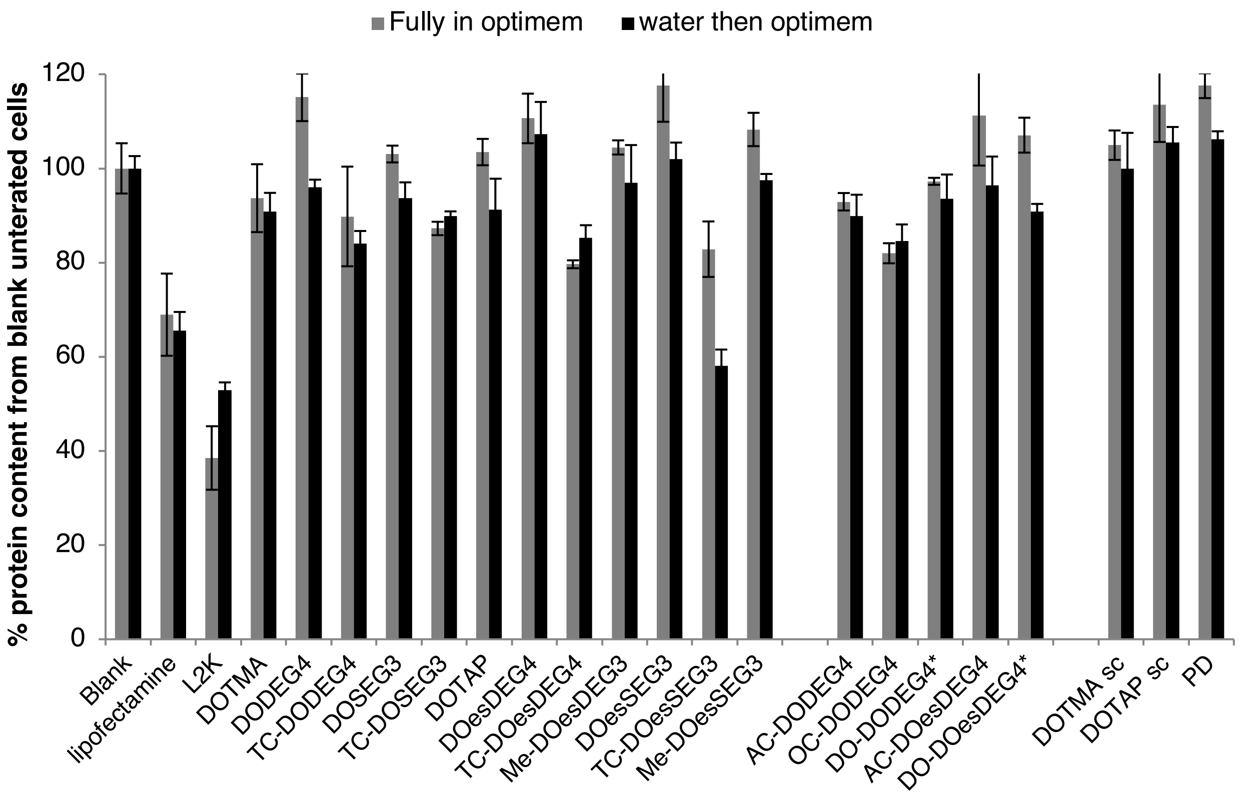

Figure S3. % Protein content compared to blank (untreated cells) of LPD complexes at 0.25:6.5:1 charge ratio prepared either fully in OptiMEM (grey bars) or in 25% v/v water then diluted with OptiMEM (black bars) in B104 cells using the various di- and trichain group 1 and 2 cationic lipids, peptide **A** and gWiz plasmid. DOTMA and DOTAP LPDs were also tested with a scrambled version of peptide **A** (sc). Peptide **A**:pDNA (PD) complexes at 6.5:1 charge ratio in the absence of lipid were also tested together with lipofectamine 5:1 and L2K 4:1 weight ratios as controls. Data is the mean of three measurements ± standard deviation.

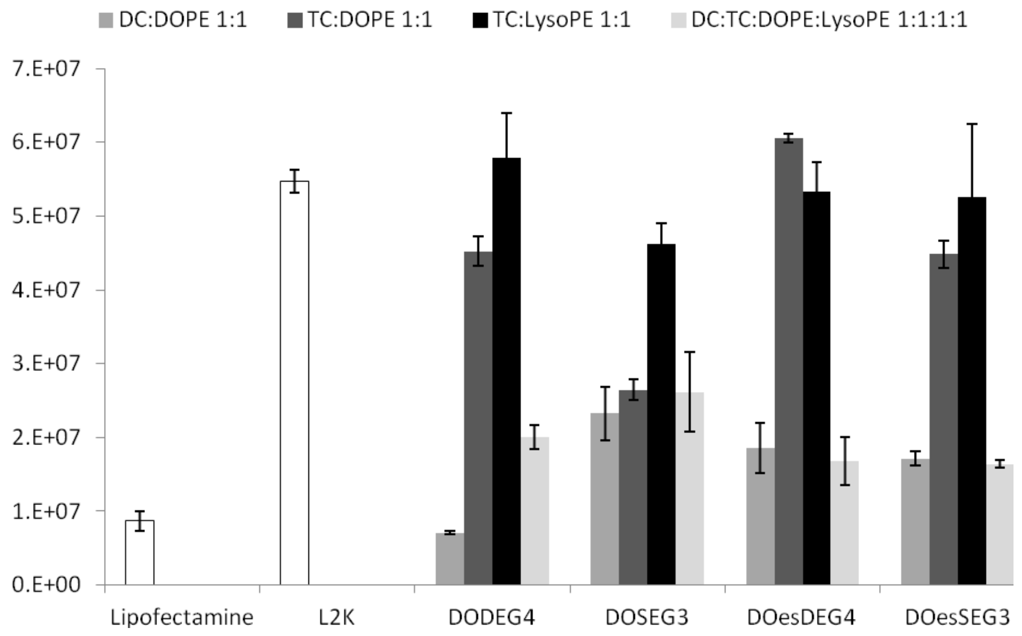

Figure S4 % transfection in B104 cells of LPDs prepared at a charge ratio of 0.25:6.5:1 using the lipids DODEG4, DOSEG3, DOesDEG4 and DOesSEG3 with 50 mol% DOPE, TC-DODEG4, TC-DOSEG3, TC-DOesDEG4 and TC-DOesSEG3 with 50 mol% DOPE, TC-DODEG4, TC-DOSEG3, TC-DOesDEG4 and TC-DOesSEG3 with 50 mol% lysoPE and an equimolar mixture of DODEG4:TC-DODEG4, DOSEG3:TC-DOSEG3, DOesDEG4:TC-DOesDEG4 and DOesSEG3:TC- DOesSEG3 with an equimolar mixture of DOPE:lysoPE. Data is the mean of three measurements ± standard deviation.

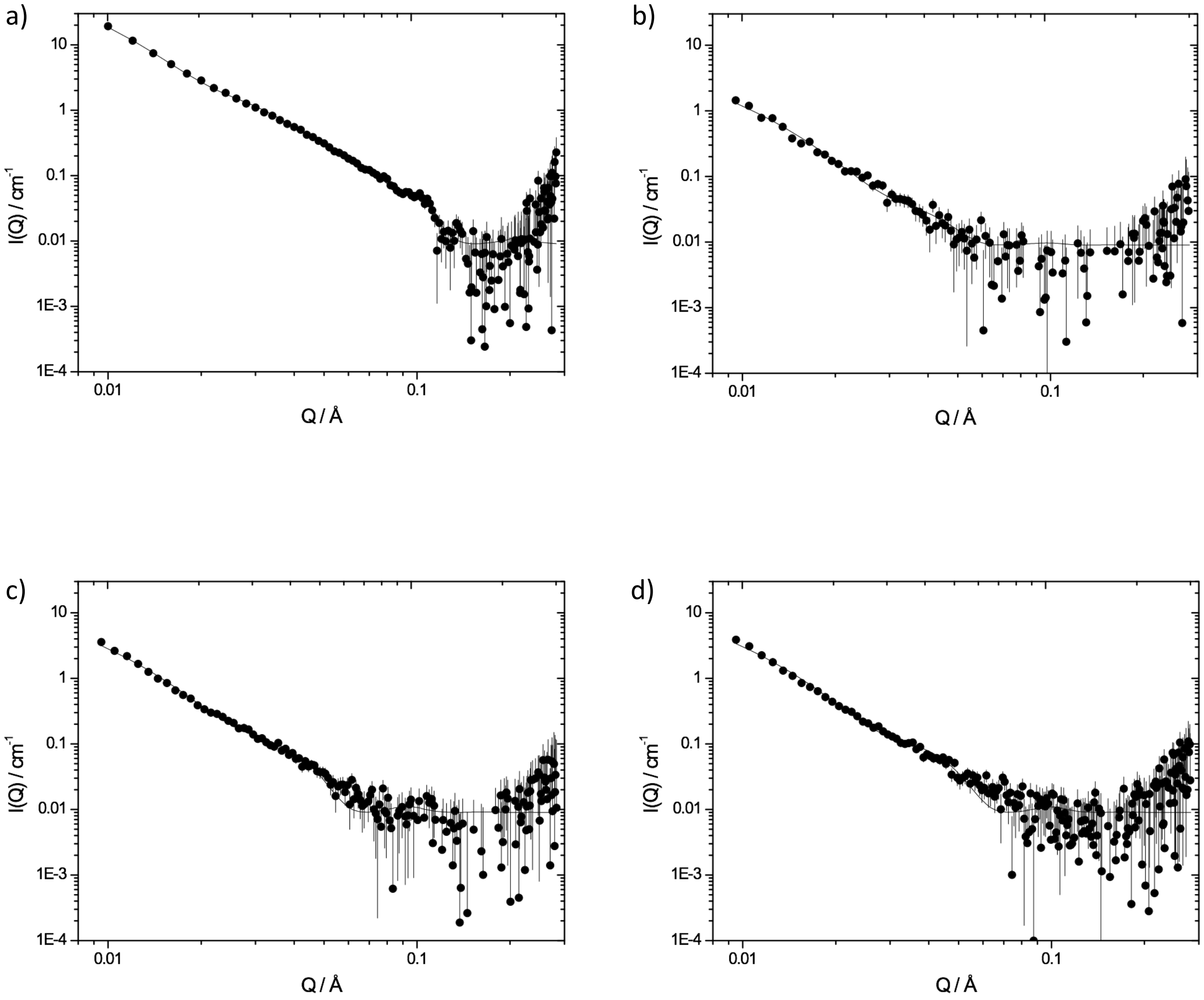

Figure S5. Small angle neutron scattering data (dots) recorded at 298 K and the best fit to the data (solid line) obtained using the mixed sheet and stack model for a) freshly prepared vesicles prepared from DOPE and 1:1 molar ratio of the cationic lipid TC-DODEG4 at a cationic lipid concentration of 1 mg/mL and LPDs prepared from these vesicles containing b) peptide **K16** at a ctDNA concentration of 0.0625 mg/mL, c) peptide **K16** at a ctDNA concentration of 0.15 mg/mL and d) peptide **A** at a ctDNA concentration of 0.15 mg/mL, all at a L:P:D charge ratio of 0.5:6:1.

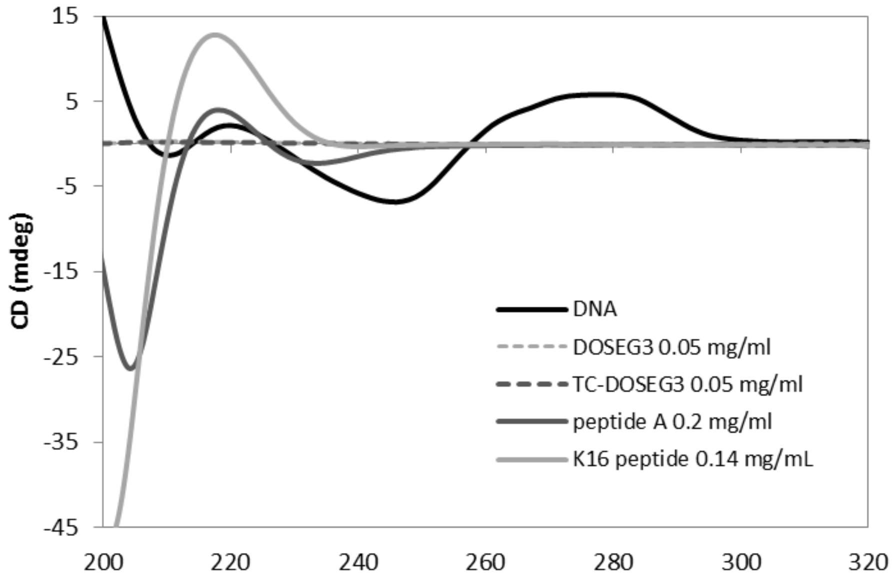

Figure S6. CD spectra of ctDNA, free peptides (peptide **A** and **K16**) and a DC (DOSEG3) and TC (TC-DOSEG3) lipid at the same concentration as used to prepare the LPDs.

References

1 A. B. Pangborn, M. A. Giardello, R. H. Grubbs, R. K. Rosen and F. J. Timmers, *Organometallics,* 1996, **15**, 1518.

2 C. A. Hurley, J. B. Wong, H. C. Hailes and A. B. Tabor, *J. Org. Chem.,* 2004, **69**, 980.

3 A. S. Narang, L. Thoma, D. D. Miller and R. I. Mahato, *Bioconjugate Chem*., 2005, **16**, 156.

4 J. B. Wong, S. Grosse, A. B. Tabor, S. L. Hart and H. C. Hailes, *Mol. BioSyst.,* 2008, **4**, 532.

5 C. A. Hurley, J. B. Wong, J. Ho, M. Writer, S. A. Irvine, M. J. Lawrence, S. L. Hart, A. B. Tabor and H. C. Hailes, *Org. Biomol. Chem.*, 2008, **6**, 2554.

6 M. F. M. Mustapa, S. M. Grosse, L. Kudsiova, M. Elbs, E.-A. Raiber, J. B. Wong, A. P. R. Brain, H. E. J. Armer, A. Warley, M. Keppler, T. Ng, M. J. Lawrence, S. L. Hart, H. C. Hailes and A. B. Tabor, *Bioconjugate Chem*., 2009, **20**, 518.

7 T. W. Brockmann and J. M. Tour, *J. Am. Chem. Soc.*, 1995, **117**, 4437.

8 L. Kudsiova, B. Fridrich, J. Ho, M. F. M. Mustapa, F. Campbell, K. Wesler, M. Keppler, T. Ng, D. Barlow, A. B. Tabor, H. C. Hailes and M. J. Lawrence, *Mol. Pharm.*, 2011, **8***,* 1831.

9. R. K. Heenan, 1989. The "Fish" reference manual. Data fitting program for small-angle 716 neutron scattering. RAL report 89-129 (Revised 2000), Rutherford Appleton Laboratory, UK.
